# Cerium Photocatalyst in Action: Structural Dynamics in the Presence of Substrate Visualized via Time-Resolved X-ray Liquidography

**DOI:** 10.1021/jacs.3c08166

**Published:** 2023-10-19

**Authors:** Yunbeom Lee, Hosung Ki, Donghwan Im, Seunghwan Eom, Jain Gu, Seonggon Lee, Jungmin Kim, Yongjun Cha, Kyung Won Lee, Serhane Zerdane, Matteo Levantino, Hyotcherl Ihee

**Affiliations:** † Center for Advanced Reaction Dynamics, Institute for Basic Science (IBS), Daejeon, 34141, Republic of Korea; ‡ Department of Chemistry and KI for the BioCentury, Korea Advanced Institute of Science and Technology (KAIST), Daejeon, 34141, Republic of Korea; § European Synchrotron Radiation Facility (ESRF), 71 Avenue des Martyrs, 38000 Grenoble, France

## Abstract

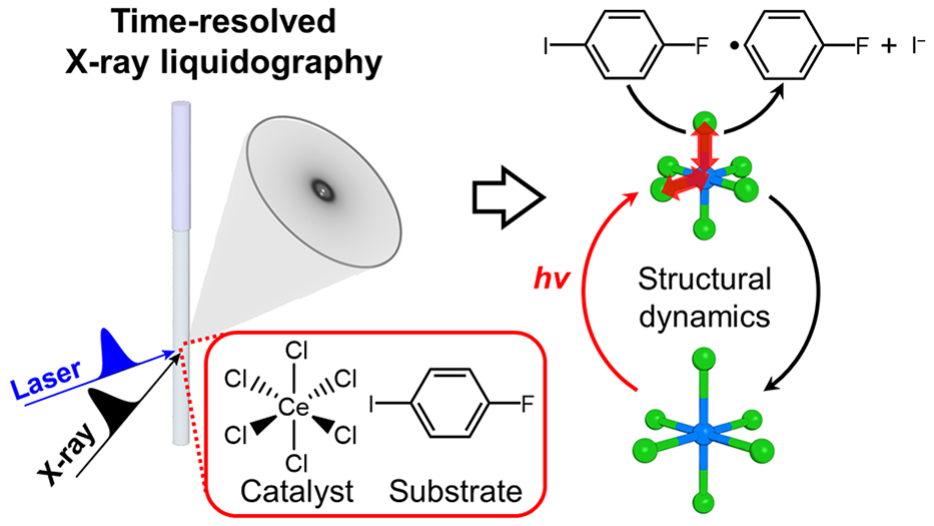

[Ce(III)Cl_6_]^3–^, with its earth-abundant metal element, is a promising photocatalyst facilitating carbon–halogen bond activation. Still, the structure of the reaction intermediate has yet to be explored. Here, we applied time-resolved X-ray liquidography (TRXL), which allows for direct observation of the structural details of reaction intermediates, to investigate the photocatalytic reaction of [Ce(III)Cl_6_]^3–^. Structural analysis of the TRXL data revealed that the excited state of [Ce(III)Cl_6_]^3–^ has Ce–Cl bonds that are shorter than those of the ground state and that the Ce–Cl bond further contracts upon oxidation. In addition, this study represents the first application of TRXL to both photocatalyst-only and photocatalyst-and-substrate samples, providing insights into the substrate’s influence on the photocatalyst’s reaction dynamics. This study demonstrates the capability of TRXL in elucidating the reaction dynamics of photocatalysts under various conditions and highlights the importance of experimental determination of the structures of reaction intermediates to advance our understanding of photocatalytic mechanisms.

## Introduction

The activation of chemical bonds through photoactivation has emerged as a central topic in the field of chemical synthesis.^1−6^ Photocatalysts or photosensitizers, primarily Ir and Ru complexes, have been extensively utilized as a means for photoactivation. These complexes have been employed in various photocatalytic reactions due to their long excited-state lifetimes and the ease of adjusting energy levels between excited and ground states through ligand modifications. A major drawback of these catalysts is their scarcity in the Earth’s crust and their high cost. These disadvantages make it difficult to use rare-earth metals for massive production in the industry. Therefore, many efforts have been made to overcome such limitations. A notable example is substituting these metals with more abundant metals, such as the first-row transition metals like Fe or Co.^7−15^ While some first-row transition-metal-based photocatalysts have been successful, many have been challenging to use due to the short lifetimes of their excited states. Another approach involves using catalysts containing lanthanides such as Ce. In particular, Ce is more abundant in the Earth’s crust than Cu, making it relatively easy to obtain. To utilize the abundance of Ce in the Earth’s crust, many recent studies have focused on Ce-based photocatalysts.^16−27^ Ce-based photocatalytic systems have shown great promise for efficient and cost-effective photocatalysis, enabling the activation of various bonds including C–H,^20,22,24,25,27^ C–X (X = Cl, Br, and I),^16,18,19^ and even C–C bonds.^17,21,23,26^ However, to fully utilize the potential of Ce-based photocatalysts, it is essential to systematically investigate their physical and chemical properties, such as their lifetimes and molecular structures, in their catalytically active forms. This would allow for the rational design of catalysts with the desired properties and the unlocking of the full potential of Ce-based photocatalysts. However, despite the importance of the structural information on the active species in photocatalytic reactions, there is a lack of successful research experimentally elucidating the detailed molecular structure of the excited state. Therefore, clear mechanistic studies have also been limited, which hinder the improvement and regulation of photocatalyst function.

To address these challenges, we employed time-resolved X-ray liquidography (TRXL), a technique that directly provides information about the transient structure of molecules in the liquid solution phase. TRXL is a potent technique that combines the pump–probe scheme with X-ray scattering, enabling extraction of molecular structures of short-lived species in solution during chemical reactions from the change in the X-ray scattering signal over time.^28,29^ Indeed, TRXL has been demonstrated as a powerful tool for investigating the structural dynamics of metal complexes, including metal photocatalysts.^30−45^ However, prior to this work, TRXL has not been applied to a solution containing both a photocatalyst and its substrate; in essence, TRXL had not been used to study a photocatalyst “in action”. To fill this gap, in this study, we aim to exploit the advantages of TRXL to investigate the structural dynamics, including the structures of reaction intermediates, of a Ce-based photocatalyst with its substrate.

**Figure 1:**
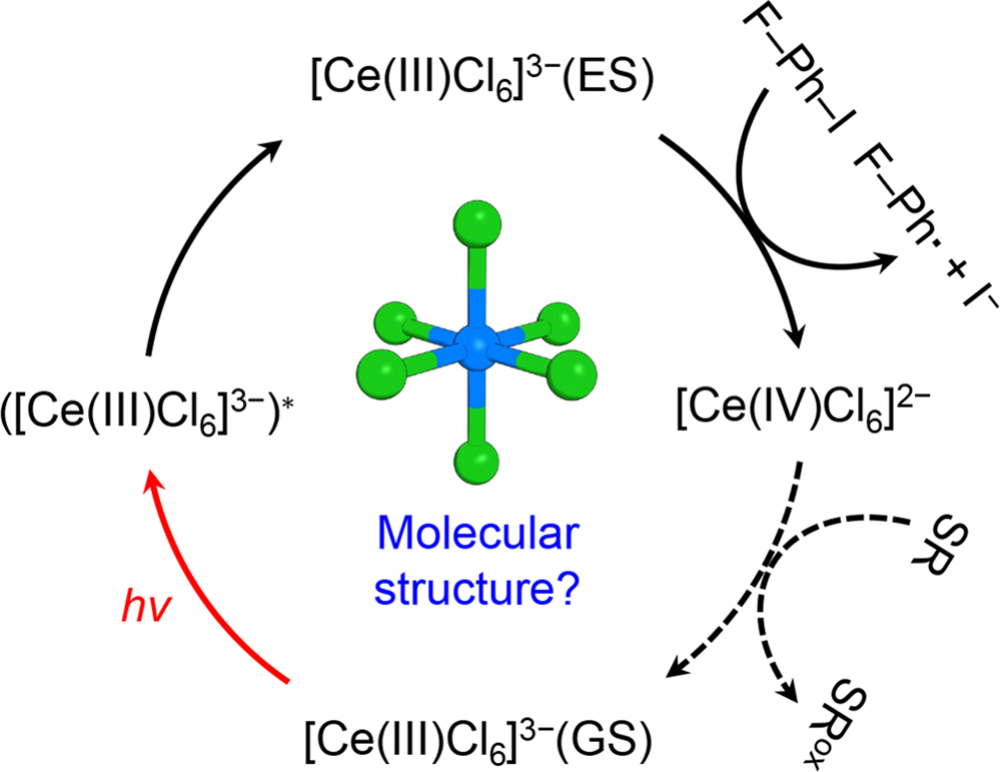
Proposed scheme for the photocatalytic C–I bond activation of a substrate using [Ce(III)Cl_6_]^3–^. Photoexcitation of [Ce(III)Cl_6_]^3–^ in the ground state ([Ce(III)Cl_6_]^3–^(GS)) generates the excited state, [Ce(III)Cl_6_]^3–^(ES), via the Franck–Condon region (([Ce(III)Cl_6_]^3–^)*). [Ce(III)Cl_6_]^3–^(ES) facilitates the C–I bond activation, resulting in the conversion of 1-fluoro-4-iodobenzene (F–Ph–I) to fluorobenzene radical (F–Ph^•^) and iodide ion (I^–^). Concurrently, [Ce(III)Cl_6_]^3–^(ES) is oxidized to [Ce(IV)Cl_6_]^2–^. Subsequently, [Ce(III)Cl_6_]^3–^(GS) can be regenerated from [Ce(IV)Cl_6_]^2–^ via reaction with a sacrificial reagent. SR and SR^ox^ indicate a sacrificial reagent and its oxidized form, respectively. The detailed reaction mechanism, including the molecular structure of the reaction intermediate, remains unresolved.

Here, we studied the structural dynamics of [Ce(III)Cl_6_]^3–^ using TRXL. [Ce(III)Cl_6_]^3–^ is a photocatalyst that can absorb UV light and functionalize C–X bonds (X = Cl, Br, and I). In a previous study, the reaction scheme for the photocatalysis of [Ce(III)Cl_6_]^3–^ was proposed (Figure 1), and the photophysical properties of [Ce(III)Cl_6_]^3–^, such as emission lifetime, have been reported as well.^19^ However, capturing the intermediate formed during the reaction of [Ce(III)Cl_6_]^3–^ with substrates has been a challenging task. The analysis of the TRXL data enabled us to obtain the structural information on the Ce photocatalyst during its role as a photocatalyst: the molecular structures of the excited state of [Ce(III)Cl_6_]^3–^ ([Ce(III)Cl_6_]^3–^(ES)) and the oxidized form ([Ce(IV)Cl_6_]^2–^) as well as the ground-state [Ce(III)Cl_6_]^3–^ ([Ce(III)Cl_6_]^3–^(GS)). In particular, it should be emphasized that the TRXL experiments were conducted on samples with and without the addition of the substrate (1-fluoro-4-iodobenzene) participating in the photoreaction of [Ce(III)Cl_6_]^3–^, which allowed for observing perturbations in the reaction pathway of the photocatalyst by the presence of the substrate.

## Results and Discussion

**Figure 2:**
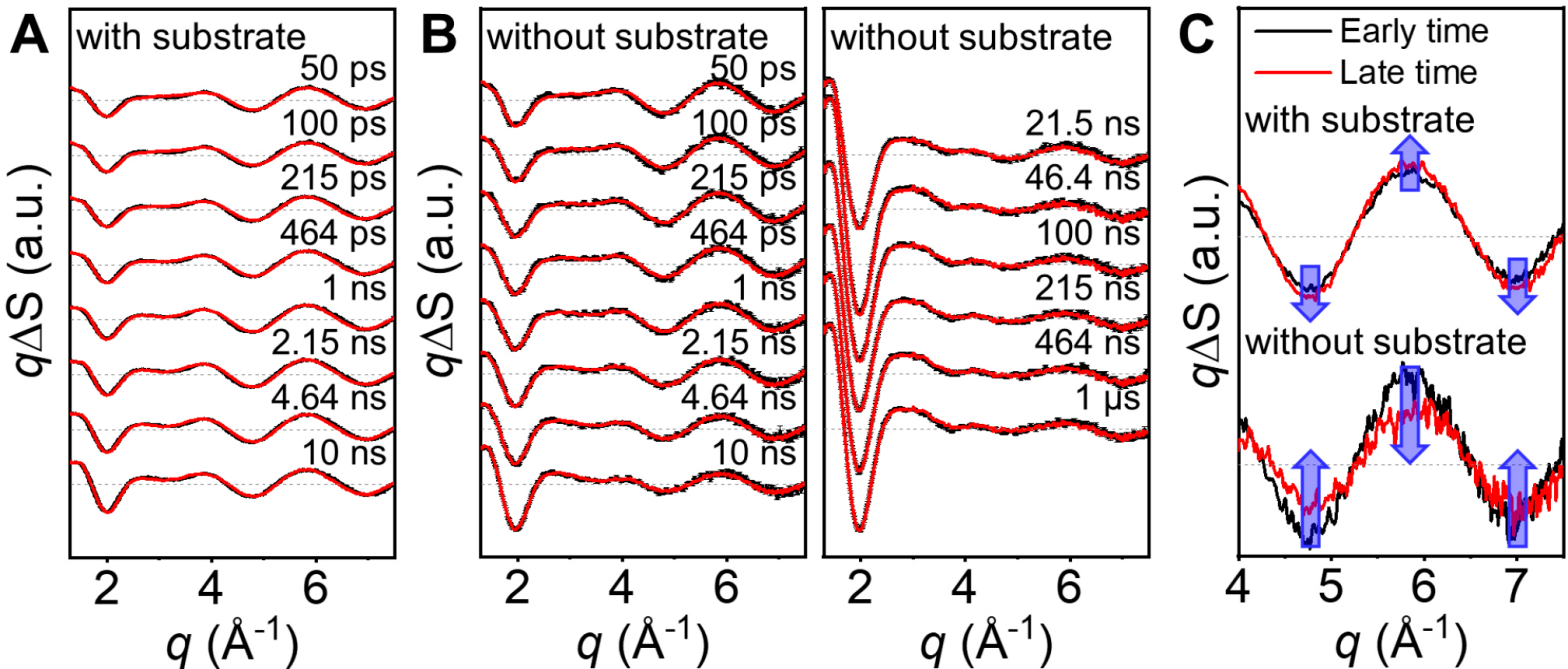
Difference scattering curves of the [Ce(III)Cl_6_]^3–^/substrate and [Ce(III)Cl_6_]^3–^-only samples and the difference scattering curves of two samples at representative early and late time delays. (A, B) The experimental (black) and theoretical (red) difference scattering curves of the [Ce(III)Cl_6_]^3–^/substrate (A) and [Ce(III)Cl_6_]^3–^-only (B) samples. The details of the calculation of the theoretical difference scattering curves are described in the “Calculation of theoretical difference scattering curves” section in the Supporting Information (SI). The data are presented as mean values ± standard errors of the mean (SEM). (C) Comparison of the difference scattering curves at the early (black) and late (red) time delays for the [Ce(III)Cl_6_]^3–^/substrate (top) and [Ce(III)Cl_6_]^3–^-only (bottom) samples. For both samples, the difference scattering curves at 50 ps and 10 ns are used for the representative early and late time delays. Blue arrows indicate changes in the difference scattering curves over time.

The difference scattering curves, ΔS(*q*, *t*), were obtained by subtracting the azimuthally averaged X-ray scattering signals collected at a negative time delay from those collected at positive time delays. The ΔS(*q*, *t*)s are expressed as a function of two variables: (i) the magnitude of the momentum transfer vector (*q*), which is related to the X-ray wavelength and the scattering angle, and (ii) the time delay between the optical laser and X-ray pulses (*t*). To better represent the signal at high *q*, in which the contribution from the structural changes of solute molecules is dominant, the difference curves are multiplied by *q* to give *q*ΔS(*q*, *t*). Figure 2A shows *q*ΔS(*q*, *t*) for the [Ce(III)Cl_6_]^3–^/substrate sample, which contains both the photocatalyst and substrate, while Figure 2B shows the *q*ΔS(*q*, *t*) for the [Ce(III)Cl_6_]^3–^-only sample, which contains only the photocatalyst. As shown in Figure 2A and B, the *q*ΔS(*q*, *t*)s show a prominent signal in the high-*q* region, indicating the structural change in solute molecules. A noticeable oscillatory feature in the high-*q* region of *q*ΔS(*q*, *t*) is evident even at the earliest time delay (50 ps) for both [Ce(III)Cl_6_]^3–^/substrate and [Ce(III)Cl_6_]^3–^-only samples. The presence of an oscillatory feature in the high-*q* region indicates that a structural change in the photocatalyst occurs within 50 ps after photoexcitation. Comparison of the *q*ΔS(*q*, *t*)s obtained from the two samples shows a highly similar shape in *q*-space that is maintained over the entire time range of 50 ps to 1 μs, particularly in the high-*q* region above *q* > 3 Å^–1^ (Figure 2A and B). Nevertheless, a detailed inspection reveals that the signal amplitude exhibits time-dependent changes. Importantly, these changes display distinct trends depending on whether the substrate is present or not. In the [Ce(III)Cl_6_]^3–^/substrate sample, the signal amplitude shows a slight increase over time, while in the [Ce(III)Cl_6_]^3–^-only sample, the signal amplitude significantly decreases over time. To more clearly visualize these trends, we plotted and compared the data at early (50 ps) and late (10 ns) time delays in Figure 2C. The observed differences in the time-dependent changes of signal amplitudes indicate that the presence of the substrate alters the structural dynamics of the photocatalyst upon photoexcitation.

The difference scattering curves are influenced by structural changes in the solute, solvent cage surrounding the solute, and bulk solvent. Among these, the structural changes related to the solute, the structural changes in the solute molecules and solvent cages, are of primary interest. Considering this, the influence of the solvent, such as changes in temperature and density of the bulk solvent, on the kinetics of ΔS(*q*, *t*) was eliminated by applying the projection to extract the perpendicular component (PEPC) method to ΔS(*q*, *t*).^46^ The resulting PEPC-treated difference scattering curves, ΔS(*q*, *t*)^⊥^, only contain the kinetics information on the solute species, the photoactivated [Ce(III)Cl_6_]^3–^, and substrate, if present (Figure S1). The symbol ⊥, denoted as a superscript, signifies the term “PEPC-treated”, which refers to the perpendicular component extracted through the PEPC process. To systematically investigate the time dependence of the difference scattering curves, we performed singular value decomposition (SVD) on the PEPC-treated TRXL data (Figure S2). SVD decomposes experimental data into left singular vectors (LSVs), right singular vectors (RSVs), and singular values. For TRXL data, LSVs represent time-independent difference scattering curves, RSVs show the time profiles of LSVs, and singular values indicate the relative contribution of each singular vector to the data. The SVD analysis indicated that only one component significantly contributed to the data for each sample. To quantitatively analyze the temporal change in the difference scattering curves, we kinetically analyzed the first RSV by fitting it with a sum of a constant and an exponential function.

**Figure 3:**
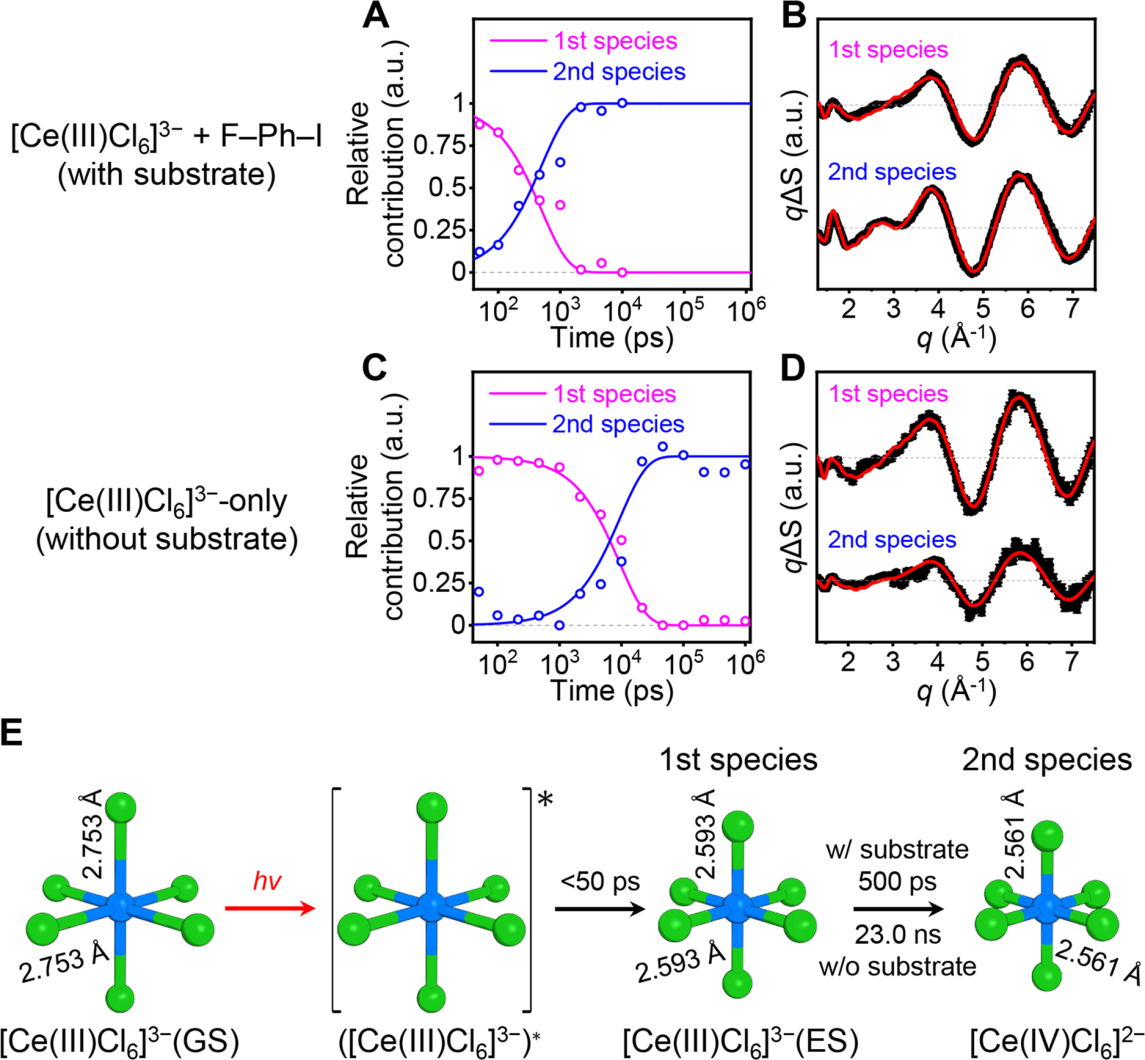
Kinetic analysis and structure refinement for the [Ce(III)Cl_6_]^3–^/substrate and [Ce(III)Cl_6_]^3–^-only samples. (A, C) Time-resolved populations of the first (magenta) and second (blue) species involved in the photoreaction of the [Ce(III)Cl_6_]^3–^/substrate (A) and [Ce(III)Cl_6_]^3–^-only (C) samples. The open circles indicate the optimal contributions of the species obtained by fitting the experimental data using the linear combination of the PEPC-treated SADSs (*q*A(*q*)^⊥^s), and the solid lines indicate the relative contributions of the species obtained from the kinetic models. The first and second species were assigned to the excited and oxidized states, respectively. (B, D) The *q*A(*q*)^⊥^ curves (black) of the first and second species of the [Ce(III)Cl_6_]^3–^/substrate (B) and [Ce(III)Cl_6_]^3–^-only (D) samples and their fits (red) obtained via the structure refinement. The *q*A(*q*)^⊥^s are presented as mean values ± SEM. (B) and (D) are shown in the same scale. (E) Molecular structures of the ground state ([Ce(III)Cl_6_]^3–^(GS)), excited state ([Ce(III)Cl_6_]^3–^(ES)), and oxidized state ([Ce(IV)Cl_6_]^2–^) determined via the structure refinement using *q*A(*q*)^⊥^s. The cerium and chloride atoms are shown in blue and green, respectively. The lengths of the axial and equatorial Ce–Cl bonds are indicated. The associated errors and comparisons with those obtained via X-ray crystallography, EXAFS, and quantum calculations are presented in Table 1. The time constants above and below the arrow correspond to the [Ce(III)Cl_6_]^3–^/substrate and [Ce(III)Cl_6_]^3–^-only samples, respectively. For the [Ce(III)Cl_6_]^3–^-only sample, the time constant for the oxidation is shown instead of the apparent time constant for the decay of [Ce(III)Cl_6_]^3–^(ES), which involves two competing processes of oxidation to [Ce(IV)Cl_6_]^2–^ and recovery to [Ce(III)Cl_6_]^3–^(GS). The recovery to [Ce(III)Cl_6_]^3–^(GS) can occur via either energy transfer from [Ce(III)Cl_6_]^3–^(ES) to oxygen or emission. The bond lengths of the molecular structures are exaggerated for clear visualization.

In the case of the [Ce(III)Cl_6_]^3–^/substrate sample, the fitting of the first RSV resulted in a time constant of 500 ± 20 ps for the exponential function (Figure S2D). The amplitude of the first RSV exhibits a slight increase along with the observed time constant of 500 ps. The observed increase in the signal amplitude suggests that the process with a time constant of 500 ps corresponds to the transition from the first species, which is formed before the time constant, to another species (the second species), rather than to the recovery of the first species to the ground state. The significant oscillatory feature in the high-*q* region of the difference scattering curve at 50 ps indicates that the first species is generated within 50 ps after photoexcitation. This information led us to conclude that the first species generated within 50 ps transforms to the second species with the apparent time constant of ∼500 ps. A previous study has proposed that, upon photoexcitation, [Ce(III)Cl_6_]^3–^ undergoes a reaction with the substrate leading to its oxidation to [Ce(IV)Cl_6_]^2–^.^19^ Following the scheme reported in the literature, we assigned the first and second species to [Ce(III)Cl_6_]^3–^(ES) and [Ce(IV)Cl_6_]^2–^ generated by the reaction of [Ce(III)Cl_6_]^3–^(ES) with the substrate, respectively. Based on this assignment, we constructed a simple sequential kinetic model and obtained the time profiles of the relative contributions of these species (Figures 3A and S3A).

For the [Ce(III)Cl_6_]^3–^-only data, the fitting of the first RSV resulted in a time constant of 9.4 ± 0.3 ns for the exponential function (Figure S2H), which is considerably longer than the time constant observed for the [Ce(III)Cl_6_]^3–^/substrate data (∼500 ps). Furthermore, the amplitude of the first RSV of [Ce(III)Cl_6_]^3–^-only data decreases over time, in contrast to the [Ce(III)Cl_6_]^3–^/substrate data. It is noticeable that the time constant obtained from TRXL is different from the reported lifetime of the excited state (∼22 ns) obtained from time-correlated single photon counting (TCSPC) measured under inert gas conditions.^19^ In addition, the difference scattering curve at 1 μs shows a clear signal due to the structural change of the photocatalyst, suggesting that not all populations of the excited state recover to the ground state through radiative decay. We hypothesized that the inconsistent lifetimes and long-lived non-ground-state species may be attributed to the presence of oxygen. To verify this hypothesis, we measured the excited-state lifetimes under ambient and N_2_-purged conditions using TCSPC and found that the presence of oxygen significantly affected the lifetime, with values of 10.02 ± 0.02 ns under ambient conditions and 25.07 ± 0.04 ns under N_2_-purged conditions (Figure S4B). The latter is similar to the reported lifetime, which was obtained under inert gas conditions, while the former was similar to the time constant obtained from TRXL, which was performed under ambient conditions. The dependence of the excited-state lifetime on the presence of oxygen implies that [Ce(III)Cl_6_]^3–^(ES) can react with oxygen, and the fact that the emission from [Ce(III)Cl_6_]^3–^(ES) is still observed under the presence of oxygen implies that the pathway of reaction of [Ce(III)Cl_6_]^3–^(ES) with oxygen is competing with another reaction pathway where [Ce(III)Cl_6_]^3–^(ES) radiatively decays to [Ce(III)Cl_6_]^3–^(GS). We proposed that the former pathway, the reaction of [Ce(III)Cl_6_]^3–^(ES) with oxygen, can proceed through two competing pathways: (1) oxidation via electron transfer and (2) nonradiative decay to the ground state via energy transfer, because a model that only considers the pathway of oxidation due to oxygen (corresponding to (1)) did not provide a satisfactory fit to our data. This suggests that there are additional pathways or processes involved, which we propose to be energy transfer to oxygen (corresponding to (2)). Both competing pathways proposed for the reaction with oxygen are feasible from an energetic standpoint. According to a previous study, the estimated reduction potential of [Ce(III)Cl_6_]^3–^(ES) is approximately −3.45 V (vs Cp_2_Fe^0/+^, where Cp_2_Fe is ferrocene),^19^ which is larger than the potential required to reduce oxygen (approximately −1.3 V vs Cp_2_Fe^0/+^),^47^ indicating that the oxidation of [Ce(III)Cl_6_]^3–^ by oxygen is possible. Through the oxidation reaction, [Ce(III)Cl_6_]^3–^(ES) and oxygen would become [Ce(IV)Cl_6_]^2–^ and a superoxide anion, respectively. In addition to the oxidation via electron transfer, energy transfer from the photocatalyst to oxygen is feasible, as the energy gap between the first excited state and the ground state of oxygen (∼0.97 eV) is smaller than that of the photocatalyst (∼3.5 eV) obtained from the emission spectrum.^48^ Further details can be found in the “Kinetic analysis” section of the SI.

After considering the competing pathways for the reaction with oxygen, we concluded that only a fraction of [Ce(III)Cl_6_]^3–^(ES) would undergo oxidation to form [Ce(IV)Cl_6_]^2–^. Based on this analysis, we can justify our assignment of the same [Ce(IV)Cl_6_]^2–^ species as the second species for both the [Ce(III)Cl_6_]^3–^-only sample and the [Ce(III)Cl_6_]^3–^/substrate sample, despite the different trends observed in the amplitude in the first RSVs. In the [Ce(III)Cl_6_]^3–^/substrate sample, the oxidation of [Ce(III)Cl_6_]^3–^(ES) to [Ce(IV)Cl_6_]^2–^ is indicated by a slight increase in the amplitude of the TRXL signal. In contrast, in the [Ce(III)Cl_6_]^3–^-only sample, only a small fraction of [Ce(III)Cl_6_]^3–^(ES) undergoes the oxidation, while the remaining fraction returns to [Ce(III)Cl_6_]^3–^(GS). Therefore, although the same oxidation reaction of [Ce(III)Cl_6_]^3–^(ES) is observed in both samples, the amplitude of the TRXL signal decreases in the [Ce(III)Cl_6_]^3–^-only sample due to the limited participation of [Ce(III)Cl_6_]^3–^(ES) in the oxidation reaction. We assigned the first and second species to [Ce(III)Cl_6_]^3–^(ES) and [Ce(IV)Cl_6_]^2–^, respectively, and established a sequential kinetic model in which the first species formed within 50 ps is transformed into the second species with an apparent time constant of 9.4 ns (Figure S3B). Then, the time profiles of the relative contributions of the two species were obtained from the kinetic model (Figure 3C). The contribution of the two slow reaction pathways, the radiative decay of [Ce(III)Cl_6_]^3–^(ES) and the reaction of [Ce(III)Cl_6_]^3–^(ES) with oxygen, on the [Ce(III)Cl_6_]^3–^/substrate sample is expected to be negligible, as the observed time constant (∼500 ps) in the sample is more than 10 times faster than the time constant (∼9.4 ns) observed for the two slow reactions.

**Figure 4:**
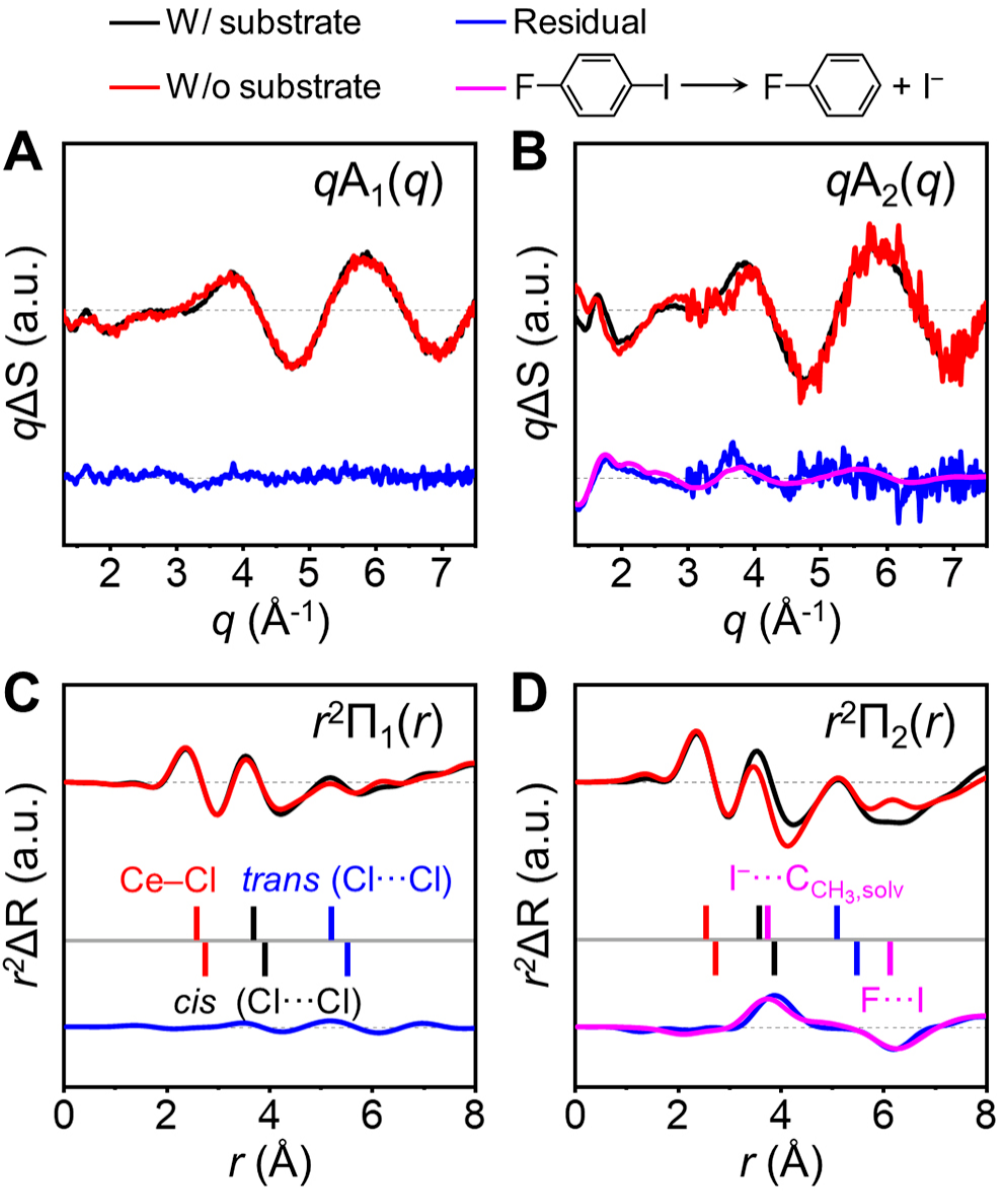
Contribution of the reaction of the substrate, 1-fluoro-4-iodobenzene (F–Ph–I), to the SADSs of the [Ce(III)Cl_6_]^3–^/substrate data. (A) The *q*A_1_(*q*)s, (B) *q*A_2_(*q*)s, (C) *r*^2^Π_1_(*r*)s, and (D) *r*^2^Π_2_(*r*)s of the [Ce(III)Cl_6_]^3–^/substrate and [Ce(III)Cl_6_]^3–^-only data. The *q*A_1_(*q*) in (A) and *q*A_2_(*q*) in (B) were obtained from *q*A_1_(*q*)^⊥^ and *q*A_2_(*q*)^⊥^ after correcting the distortion due to the PEPC treatment. Those without such correction (*q*A_1_(*q*)^⊥^ and *q*A_2_(*q*)^⊥^) are shown in Figures 3B and D and S5. The *r*^2^Π_1_(*r*)s and *r*^2^Π_2_(*r*)s were obtained by performing Fourier sine transforms on *q*A_1_(*q*)s and *q*A_2_(*q*)s, respectively. In all panels, the curve for the [Ce(III)Cl_6_]^3–^/substrate is shown in black, and that of the [Ce(III)Cl_6_]^3–^-only is shown in red. For comparison, the scales of the curves for the [Ce(III)Cl_6_]^3–^-only and [Ce(III)Cl_6_]^3–^/substrate were adjusted. In all panels, the residual obtained by subtracting the curve of the [Ce(III)Cl_6_]^3–^-only from that of the [Ce(III)Cl_6_]^3–^/substrate is shown in blue. The residuals for *q*A_1_(*q*) in (A) and *r*^2^Π_1_(*r*) in (C) are negligible, indicating that the SADSs of the [Ce(III)Cl_6_]^3–^/substrate and [Ce(III)Cl_6_]^3–^-only are identical in both *q*- and *r*-spaces. In contrast, the residuals for *q*A_2_(*q*) in (B) and *r*^2^Π_2_(*r*) in (D) are not negligible and exhibit a distinct feature, indicating that the SADSs of the [Ce(III)Cl_6_]^3–^/substrate in *q*- and *r*-spaces are different from those of the [Ce(III)Cl_6_]^3–^-only. In fact, these residuals can be explained by the theoretical *q*ΔS(*q*) due to the reaction of the substrate (shown in magenta) in (B) and its theoretical difference radial distribution function (shown in magenta) in (D). The theoretical difference scattering curve of the reaction of the substrate was calculated considering the dissociation of F–Ph–I to fluorobenzene (F–Ph) and iodide ion (I^–^). In (C) and (D), the vertical bars indicate the distances between the Ce and Cl atoms (red), those between the Cl atoms in the *cis* position (black), and those between the Cl atoms in the *trans* position (blue) of the photocatalyst. The magenta vertical bars indicate the distances between the atoms related to the reaction of the substrate. F···I indicates the distance between F and I atoms in the substrate, and I^–^···C_CH_3_,solv_ indicates that between I^–^ and the C atom of the methyl group in an acetonitrile molecule. The vertical bars above the gray solid line indicate the distances in the products ([Ce(III)Cl_6_]^3–^(ES), [Ce(IV)Cl_6_]^2–^, F–Ph, and I^–^), whereas those below the gray solid line indicate the distances in the reactants ([Ce(III)Cl_6_]^3–^(GS) and F–Ph–I).

Based on the kinetic model determined for each sample (Figures 3A,C and S3), we extracted the species-associated difference scattering curves (SADSs) from the experimental data. Specifically, we applied kinetics-constrained analysis (KCA) on the PEPC-treated data with the kinetic models determined from the kinetic analysis to extract PEPC-treated SADSs, represented as *q*A(*q*)^⊥^, for [Ce(III)Cl_6_]^3–^(ES) and [Ce(IV)Cl_6_]^2–^.^49−53^ Here, we will use Greek capital letter A to represent the SADS in *q*-space. The symbol is derived from the first letter of the Greek term for reciprocal space, “αμοιβαίος χώρος” (amoivaíos chóros), meaning reciprocal space. This notation will be consistent throughout the article. The first PEPC-treated SADS (*q*A_1_(*q*)^⊥^) and the second PEPC-treated SADS (*q*A_2_(*q*)^⊥^), shown in Figure 3B and D, correspond to the difference scattering curves of [Ce(III)Cl_6_]^3–^(ES) and [Ce(IV)Cl_6_]^2–^, respectively. The SADSs contain information on the detailed molecular structures of not only the reaction intermediates but also the reactants, enabling their use for structural analysis. Before conducting detailed structural analysis using SADSs, we compared the shape of the *q*A_*i*_(*q*)^⊥^s (*i* = 1 and 2) obtained from the two samples, [Ce(III)Cl_6_]^3–^-only and [Ce(III)Cl_6_]^3–^/substrate (Figure S5). The comparison revealed that the shape of *q*A_1_(*q*)^⊥^ obtained from the two samples was almost identical, and the two scaled *q*A_1_(*q*)^⊥^ curves almost completely overlapped, as shown in Figure S5A. This remarkable similarity strongly supports our assignment that the species corresponding to *q*A_1_(*q*)^⊥^ is likely the same in both samples, which is [Ce(III)Cl_6_]^3–^(ES). Meanwhile, the presence of the substrate significantly perturbs the difference scattering curves, as the structural change of the substrate also contributes to the difference scattering curves in addition to the structural change of the photocatalyst (Figures 4B, S5, and S6). This results in a difference in the *q*-space shape of the *q*A_2_(*q*)^⊥^ curves between the [Ce(III)Cl_6_]^3–^/substrate sample and the [Ce(III)Cl_6_]^3–^-only sample (Figure S5B).

To determine the molecular structures of [Ce(III)Cl_6_]^3–^(GS), [Ce(III)Cl_6_]^3–^(ES), and [Ce(IV)Cl_6_]^2–^, structure refinement was performed using *q*A_1_(*q*)^⊥^ and *q*A_2_(*q*)^⊥^ of the [Ce(III)Cl_6_]^3–^/substrate sample. The details of the structure refinement are provided in the “Structure refinement” section of the SI. During the structure refinement process, two Ce–Cl bond lengths were used to describe the structure of each species. One is the Ce–Cl bond length for the four equatorial ligands, and the other is the Ce–Cl bond length for the two axial ligands. Using two Ce–Cl bond lengths instead of a single Ce–Cl bond length is intended to account for the possibility of losing octahedral symmetry. Considering that the substrate undergoes C–I bond dissociation when [Ce(IV)Cl_6_]^2–^ is formed, the contribution due to the C–I bond dissociation of the substrate was also considered in the calculation of theoretical *q*A_2_(*q*)^⊥^. The theoretical *q*A(*q*)^⊥^s obtained through the structure refinement described the experimental *q*A(*q*)^⊥^s well (Figure 3B).

Notably, the C–I bond dissociation was found to contribute significantly to the TRXL data, as indicated by its corresponding theoretical curve (magenta solid line) in Figures 4B, S5B, and S6B, which displays pronounced oscillatory feature with substantial amplitude. To quantitatively estimate the sensitivity of the TRXL signal to the C–I bond dissociation or the contribution of the C–I bond dissociation to the TRXL signal, we generated a sensitivity plot for the reaction investigated in this work (Figure S7).^54^ In terms of interatomic distances, the darkness of the line in the sensitivity plot denotes the degree of contribution of a particular interatomic distance to the TRXL signal, with darker lines indicating more dominant contributions. Meanwhile, in terms of the positions of atoms, the size of the circle in the sensitivity plot denotes the degree of contribution of an atomic position to the TRXL signal, with larger circles indicating more dominant contributions. The sensitivity plot confirms that the C–I bond length and the position of the iodine atom, of which the sensitivity is represented as the gray line and medium-sized circle, respectively, make a significant contribution to the TRXL data in addition to the Ce–CI bond lengths and the positions of Cl atoms, which make dominant contributions. Therefore, the sensitivity plot highlights the considerable contribution of the structural change of the substrate, C–I bond dissociation, to the overall TRXL signal.

The structural parameters for [Ce(III)Cl_6_]^3–^(GS), [Ce(III)Cl_6_]^3–^(ES), and [Ce(IV)Cl_6_]^2–^ determined from the structure refinement are presented in Figure 3E and Table 1. While the axial and equatorial Ce–Cl bond lengths were used as independent parameters, both bond lengths converged to the same value during the structure refinement, indicating that octahedral symmetry is maintained in all states, including [Ce(III)Cl_6_]^3–^(ES). The refinement result revealed that the Ce–Cl distance contracts by approximately 0.16 Å from 2.753 Å in [Ce(III)Cl_6_]^3–^(GS) to 2.593 Å in [Ce(III)Cl_6_]^3–^(ES). For comparison, the structural parameters reported from the static X-ray crystallography studies and those determined by theoretical calculations are also summarized in Table 1. It should be noted that the molecular structure of [Ce(III)Cl_6_]^3–^(ES) is not available via static X-ray crystallography. Successful determination of the structure of [Ce(III)Cl_6_]^3–^(ES) via TRXL demonstrates that TRXL can provide invaluable information about the reaction intermediates, which is typically challenging to elucidate with static X-ray crystallography. One noticeable finding is that the error of the Ce–Cl bond lengths we have determined is remarkably small, with values even reaching as low as 0.001 Å, indicating the exceptionally high resolution of the structural parameters obtained in our study. We note that the error of the Ce–Cl bond lengths indicates random error, not considering systematic error, and thus the small error implies precision rather than accuracy.

**Table 1:** Axial and Equatorial Ce–Cl Bond Lengths of [Ce(III)Cl_6_]^3–^(GS), [Ce(III)Cl_6_]^3–^(ES), and [Ce(IV)Cl_6_]^2–^ Determined via TRXL and Comparison with Those from Other Experimental Methods and Quantum Calculations

	[Ce(III)Cl_6_]^3–^(GS) (ground state)		[Ce(III)Cl_6_]^3–^(ES) (excited state)		[Ce(IV)Cl_6_]^2–^ (oxidized state)	
method	axial Ce–Cl (Å)	equatorial Ce–Cl (Å)	axial Ce–Cl (Å)	equatorial Ce–Cl (Å)	axial Ce–Cl (Å)	equatorial Ce–Cl (Å)
TRXL (this work)	2.753 ± 0.002	2.753 ± 0.001	2.593 ± 0.005	2.593 ± 0.003	2.561 ± 0.003	2.561 ± 0.001
crystallography^a^	2.78 ± 0.02^b^		-		2.61 ± 0.02^c^	
crystallography^d^	2.77 ± 0.02^e^		-		2.599 ± 0.001	
EXAFS (solid)^d^	2.79 ± 0.02		-		2.62 ± 0.02	
CASSCF/CASPT2^f^	2.816		2.757		-	
CASPT2^g^	2.85		-		2.62	
RASPT2^g^	2.86		-		2.64	
B3LYP^g^	2.82		-		2.67	
PBE^g^	2.79		-		2.65	
B3LYP (this work)^h^	2.815 (+0.062)		2.697 (+0.104)	2.776 (+0.183)	2.654 (+0.093)	
PBE0 (this work)^h^	2.783 (+0.030)		2.670 (+0.077)	2.736 (+0.143)	2.621 (+0.060)	
ωB97X (this work)^h^	2.799 (+0.046)		2.684 (+0.091)	2.776 (+0.183)	2.632 (+0.071)	
CAM-B3LYP (this work)^h^	2.799 (+0.046)		2.683 (+0.090)	2.766 (+0.173)	2.629 (+0.068)	

a Yin et al.^19^

b The mean value and standard deviation of six Ce–Cl bonds in the crystal structure. Six Ce–Cl bond lengths are 2.7988 ± 0.0006, 2.7988 ± 0.0006, 2.7790 ± 0.0006, 2.7790 ± 0.0006, 2.7612 ± 0.0006, and 2.7612 ± 0.0006 Å.

c The mean value and standard deviation of six Ce–Cl bonds in the crystal structure. Six Ce–Cl bond lengths are 2.6079 ± 0.0006, 2.6079 ± 0.0006, 2.6275 ± 0.0005, 2.6275 ± 0.0005, 2.5897 ± 0.0006, and 2.5897 ± 0.0006 Å.

d Löble et al.^55^

e The mean value and the standard deviation of six Ce–Cl bonds in the crystal structure. Six Ce–Cl bond lengths are 2.7559 ± 0.0008, 2.7682 ± 0.0008, 2.7986 ± 0.0008, 2.7663 ± 0.0008, 2.7679 ± 0.0008, and 2.7520 ± 0.0008 Å.

f Barandiarán et al.^56^ The calculations used the polarizable continuum model (PCM) to consider the effect of bulk acetonitrile solvent (ε = 38.8).

g Beekmeyer et al.^57^ The calculations used the PCM to consider the effect of bulk water solvent (ε = 78.4).

h Calculations were conducted for each basis set under three different conditions: 1. without considering both solvent and scalar relativistic effects, 2. considering solvent (conductor-like PCM, C-PCM) without considering scalar relativistic effects, and 3. considering both solvent (C-PCM) and scalar relativistic effects (ZORA). The table presents only the results corresponding to condition 3, while the results of all the calculations are presented in Tables S1–S3. The numbers in parentheses indicate the differences between the parameters obtained from the calculations and those determined from the TRXL experiments.

One of the distinct advantages of the TRXL technique is that it provides information on structural changes in real space.^28^ Each of ΔS(*q*, *t*)s and *q*A(*q*)^⊥^s obtained from the experiments display oscillations in *q*-space, which can be transformed into *r*-space using the Fourier sine transform (Figures S5 and S8). In particular, the SADSs in *r*-space, obtained from the Fourier sine transform of *q*A(*q*)s and represented as *r*^2^Π(*r*)s, provide information on the differences of the structures of [Ce(III)Cl_6_]^3–^(ES) and [Ce(IV)Cl_6_]^2–^, compared to [Ce(III)Cl_6_]^3–^(GS). We have introduced the use of the Greek capital letter Π to denote the SADS in *r*-space, recognizing the importance of distinguishing it from the previously defined SADS in *q*-space. The symbol Π is derived from the first letter of the Greek term for real space, “πραγματικó χώρο” (pragmatikó chóros). This notation will be consistently used throughout this article to distinguish between the two distinct SADSs in *q*- and *r*-spaces, respectively. It should be noted that *r*^2^Π(*r*) discussed here represents a SADS that is free from the effects of PEPC. Therefore, unlike the previously mentioned *q*A(*q*)^⊥^, *r*^2^Π(*r*) does not include this symbol. To obtain these *r*^2^Π(*r*)s, we corrected the distorted shapes of *q*A(*q*)^⊥^ in *q*-space caused by the PEPC process to obtain the PEPC-free SADSs in *q*-space, represented as *q*A(*q*) (Figure 4A and B).^46^ Then, the *q*A(*q*)s were Fourier sine transformed to obtain the PEPC-free SADSs in *r*-space, represented as *r*^2^Π(*r*)s. Figure 4C and D show the first PEPC-free SADS in *r*-space, *r*^2^Π_1_(*r*), and the second PEPC-free SADS in *r*-space, *r*^2^Π_2_(*r*), respectively. Further details can be found in the “*R*-space analysis” section of the SI. These *r*^2^Π(*r*)s offer valuable insights into the structural changes that occurred and validate our structures retrieved through the structure refinement.

The *r*-space *r*^2^Π(*r*)s display distinct oscillatory patterns, where positive and negative peaks represent the distances of newly formed and disappeared atom–atom pairs, respectively. For instance, when a particular atom–atom pair distance decreases, the negative peak is observed at the initial distance before the contraction, while the positive peak emerges at the position at the reduced distance after the contraction. Similarly, in the event of bond dissociation, the interatomic distances that disappear due to the bond dissociation are represented by the negative peak without the corresponding positive peaks. Importantly, in Figure 4C and D, both *r*^2^Π_1_(*r*) and *r*^2^Π_2_(*r*) exhibit positive peaks located to the left of their respective negative peaks, providing unequivocal evidence of bond contraction. A comparison of *r*^2^Π_1_(*r*)s for the [Ce(III)Cl_6_]^3–^-only and [Ce(III)Cl_6_]^3–^/substrate samples reveals striking similarities in their shapes, thereby confirming that the presence of the substrate has a negligible effect on the structural change associated with the first species, [Ce(III)Cl_6_]^3–^(ES). In contrast, the comparison of *r*^2^Π_2_(*r*)s for these samples uncovers significant differences, as illustrated in Figure 4D, indicating the influence of the substrate on the structural change associated with the second species, [Ce(IV)Cl_6_]^2–^. We confirmed that this difference arises from the reaction of the substrate and can be interpreted in terms of the structural change of the substrate and the concomitant change in the cage structure surrounding the substrate molecules. Specifically, *r*^2^Π_2_(*r*) for the [Ce(III)Cl_6_]^3–^/substrate sample displays a pronounced negative peak at approximately 6 Å, signifying the vanishing of the F···I atomic pair due to the C–I bond dissociation in the substrate. In addition to this, the newly established distance of around 4 Å, originating from the cage structure surrounding the newly formed I^–^ fragment, is manifested in the *r*^2^Π_2_(*r*). Overall, the *r*-space *r*^2^Π(*r*)s offer a qualitative framework for interpreting the structural changes and corroborate the structures obtained through the structure refinement. Furthermore, the *r*^2^Π(*r*)s underscore the effectiveness of our TRXL data in capturing the structural change in the substrate occurring in conjunction with the structural change of the photocatalyst.

For the analysis of the *q*A(*q*)s of the [Ce(III)Cl_6_]^3–^-only sample, the structural parameters obtained through the structure refinement of the [Ce(III)Cl_6_]^3–^/substrate data were used without modification. The theoretical *q*A(*q*)^⊥^s, calculated by using these structural parameters, demonstrate satisfactory agreement with the experimental *q*A(*q*)^⊥^s, as illustrated in Figure 3D. These results confirm that the structural changes of the photocatalyst are identical for both samples, regardless of the presence or absence of the substrate. However, upon comparison of the relative amplitudes of *q*A_2_(*q*)^⊥^ and *q*A_1_(*q*)^⊥^ for the two samples (Figure 3B and D), it becomes apparent that the relative amplitude of *q*A_2_(*q*)^⊥^ is significantly smaller in the [Ce(III)Cl_6_]^3–^-only sample compared to the [Ce(III)Cl_6_]^3–^/substrate sample. The smaller relative amplitude of SADS indicates that, considering that the amplitude is proportional to the yield of the species, the reaction yield of the oxidation of the photocatalyst ([Ce(III)Cl_6_]^3–^(ES) → [Ce(IV)Cl_6_]^2–^) is significantly lower in the absence of the substrate. In other words, a substantial fraction of [Ce(III)Cl_6_]^3–^(ES) undergoes recovery to [Ce(III)Cl_6_]^3–^(GS) instead of participating in the oxidation reaction in the sample without the substrate. By quantitatively analyzing *q*A_2_(*q*)^⊥^ for the [Ce(III)Cl_6_]^3–^-only sample, we determined the fraction of [Ce(III)Cl_6_]^3–^(ES) that undergoes the oxidation reaction. According to the results, approximately 40.9 ± 0.3% of [Ce(III)Cl_6_]^3–^(ES) was oxidized, and the remaining 59.1 ± 0.3% decayed to [Ce(III)Cl_6_]^3–^(GS). Using the apparent time constant of 9.4 ns and the fractions of [Ce(III)Cl_6_]^3–^(ES) undergoing oxidation and ground-state recovery, we calculated time constants corresponding to oxidation and ground-state recovery as 23.0 ± 0.8 and 15.9 ± 0.5 ns, respectively.

The structure of [Ce(III)Cl_6_]^3–^ has been investigated both experimentally^19,55,58,59^ and theoretically,^19,55−58,60−62^ primarily in the context of its utilization as a dopant in solid materials. A study has focused on quantum calculations using CASSCF/CASPT2 and reported the structures of [Ce(III)Cl_6_]^3–^(GS) and [Ce(III)Cl_6_]^3–^(ES) in acetonitrile, without investigating the structural parameters of [Ce(IV)Cl_6_]^2–^, as it was beyond the scope of the study.^56^ Another study employed various functionals for quantum calculations and provided the structures of [Ce(III)Cl_6_]^3–^(GS) and [Ce(IV)Cl_6_]^2–^ in water, without determining the structure of [Ce(III)Cl_6_]^3–^(ES).^57^ Additionally, one theoretical study only described the character of the electronic transition associated with 330 nm photoexcitation, without providing the corresponding structural parameters of [Ce(III)Cl_6_]^3–^(ES).^19^ To serve as a comprehensive reference for comparison with our experimental results, the three aforementioned theoretical studies have a limitation in that they did not include the structures of all three species captured in our experiments. Therefore, to address this limitation and to make a direct comparison, we performed our own calculations using density functional theory (DFT) and time-dependent DFT (TD-DFT) with multiple functionals. The resulting structural parameters, including those from other theoretical studies, are listed in Table 1 and Tables S1–S3. Our calculation results, as presented in Table 1, demonstrated variations depending on the choice of functionals employed. Despite considering the scalar relativistic effects (with zeroth-order regular approximation, ZORA) and the solvent effect (with a conductor-like polarizable continuum model, C-PCM) during the calculations, the DFT calculations still slightly overestimated the Ce–Cl bond lengths in [Ce(III)Cl_6_]^3–^(GS), [Ce(III)Cl_6_]^3–^(ES), and [Ce(IV)Cl_6_]^2–^ compared with the TRXL results. Specifically, in the case of [Ce(III)Cl_6_]^3–^(GS), the DFT calculations exhibited a slight overestimation ranging from 0.03 to 0.06 Å, depending on the choice of the functional. For [Ce(III)Cl_6_]^3–^(ES) and [Ce(IV)Cl_6_]^2–^, the TD-DFT and DFT calculations exhibit larger overestimations of the Ce–Cl bond lengths ranging from 0.08 to 0.18 Å and from 0.06 to 0.09 Å, respectively. Among the functionals employed, the PBE0 functional yielded results that were closest to the bond lengths determined by TRXL. Following PBE0, the functionals CAM-B3LYP, ωB97X, and B3LYP produced results in descending order of proximity to the TRXL-determined bond lengths. Comparisons provided in Table S1 and Table S3 clearly illustrate the influence of considering the scalar relativistic effects and solvents on the calculated structural parameters. Without considering scalar relativistic effects, the bond lengths are slightly overestimated compared to the case where these effects are considered. Neglecting the solvent effects leads to a significant overestimation of the bond lengths compared to when solvent effects are considered. We note that the observation that calculations considering both scalar relativistic effects and solvent effects provide a better description of the experimental conditions is reasonable. This is attributed to two factors: (1) the consideration of the relativistic effect is necessary due to the presence of a large number of electrons in cerium, and (2) the acetonitrile solvent affects the Ce–Cl bond length by stabilizing the solute through electrostatic interactions with the electron in the singly occupied molecular orbital (SOMO) of the solute.

Regardless of the functionals used, our calculation results consistently indicate that the Ce–Cl bond contracts in [Ce(III)Cl_6_]^3–^(ES) compared to [Ce(III)Cl_6_]^3–^(GS). In a theoretical study, where only the character of the electronic transition associated with the 330 nm photoexcitation was reported without providing structural parameters, it was assigned that the photoexcitation corresponds to an electronic transition from Ce(III) 4f orbitals to a weak Ce–Cl antibonding orbital with predominant Ce(III) t_2g_ orbital character of 5d orbitals.^19^ This assignment to a transition to an antibonding orbital may imply an elongation of Ce–Cl bonds upon the photoexcitation. However, other theoretical studies reported that Ce–Cl bonds contract, rather than elongate, upon the photoexcitation to t_2g_ orbitals.^56,58,60,61^ A conflicting interpretation was presented in a study on Ce^3+^-doped Cs_2_NaYCl_6_, suggesting that the t_2g_ orbitals exhibit bonding characteristics instead of the previously proposed antibonding characteristics with ligands.^60^ Additionally, it was suggested that upon excitation, a vacancy (hole) is formed in the 4f orbitals, resulting in a charge transfer from Cl to Ce. This charge transfer process strengthens the ionic bond character, ultimately leading to a contraction of the Ce–Cl bond. Our TD-DFT calculations confirm that 330 nm excitation has a character of the transition from 4f to 5d (t_2g_) orbitals, consistent with previous results (Figure S9). Furthermore, we find that the Ce–Cl bonds undergo contraction during the geometry optimization of the excited state (^2^T_2g_ state), which aligns with the TRXL results and other theoretical studies.

However, there is a discrepancy between the results of TRXL and our calculation results. In the calculation results, the axial Ce–Cl bonds contract significantly, while the change in the equatorial Ce–Cl bond lengths is relatively minor. In contrast, in the TRXL results, all of the Ce–Cl bonds contract equally, maintaining octahedral symmetry. We propose that the difference in the structural change arises from our basic assumption used for the TD-DFT calculations. In the TD-DFT calculations, it was assumed that an electron is excited to one of the triply degenerate t_2g_ orbitals. However, in reality, the electron would be excited to the triply degenerate t_2g_ orbitals as a group rather than to a specific orbital within the group. Considering the challenges associated with optimizing highly excited states with theoretical calculations, the discrepancy observed in our comparative study of experimental and theoretical results emphasizes the significance of direct experimental determination of the molecular structures. The detailed molecular structural parameters determined using TRXL would serve as a reliable reference for future investigations.

In terms of the experimental efforts to study the structural change of [Ce(III)Cl_6_]^3–^ upon photoexcitation, an experimental study using the pressure-dependent absorption and emission spectra confirmed that Ce–Cl bond lengths decrease upon photoexcitation of Ce^3+^-doped Cs_2_NaLuCl_6_.^59^ Another study analyzing the vibrational progression in the emission spectra of Ce-doped hexachloroelpasolites argued that the Ce–Cl bond changes by ∼0.04 Å upon photoexcitation and assigned this change as contraction based on the *ab initio* calculation results.^58^ However, the detailed structure of [Ce(III)Cl_6_]^3–^(ES) was not experimentally determined in those studies. In this study, by using TRXL, not only was the contraction of the bond length confirmed but also the detailed molecular structure of [Ce(III)Cl_6_]^3–^(ES) was visualized. [Ce(IV)Cl_6_]^2–^ determined from the structure refinement has a Ce–Cl bond length of 2.561 Å, indicating that the Ce–Cl bond lengths contract slightly further (∼0.03 Å) when [Ce(III)Cl_6_]^3–^(ES) is oxidized through reaction with the substrate. The small structural change of the photocatalyst demonstrates that TRXL is a powerful tool for revealing the structure of reaction intermediates, as it can capture minute structural changes of the target molecules.

**Figure 5:**
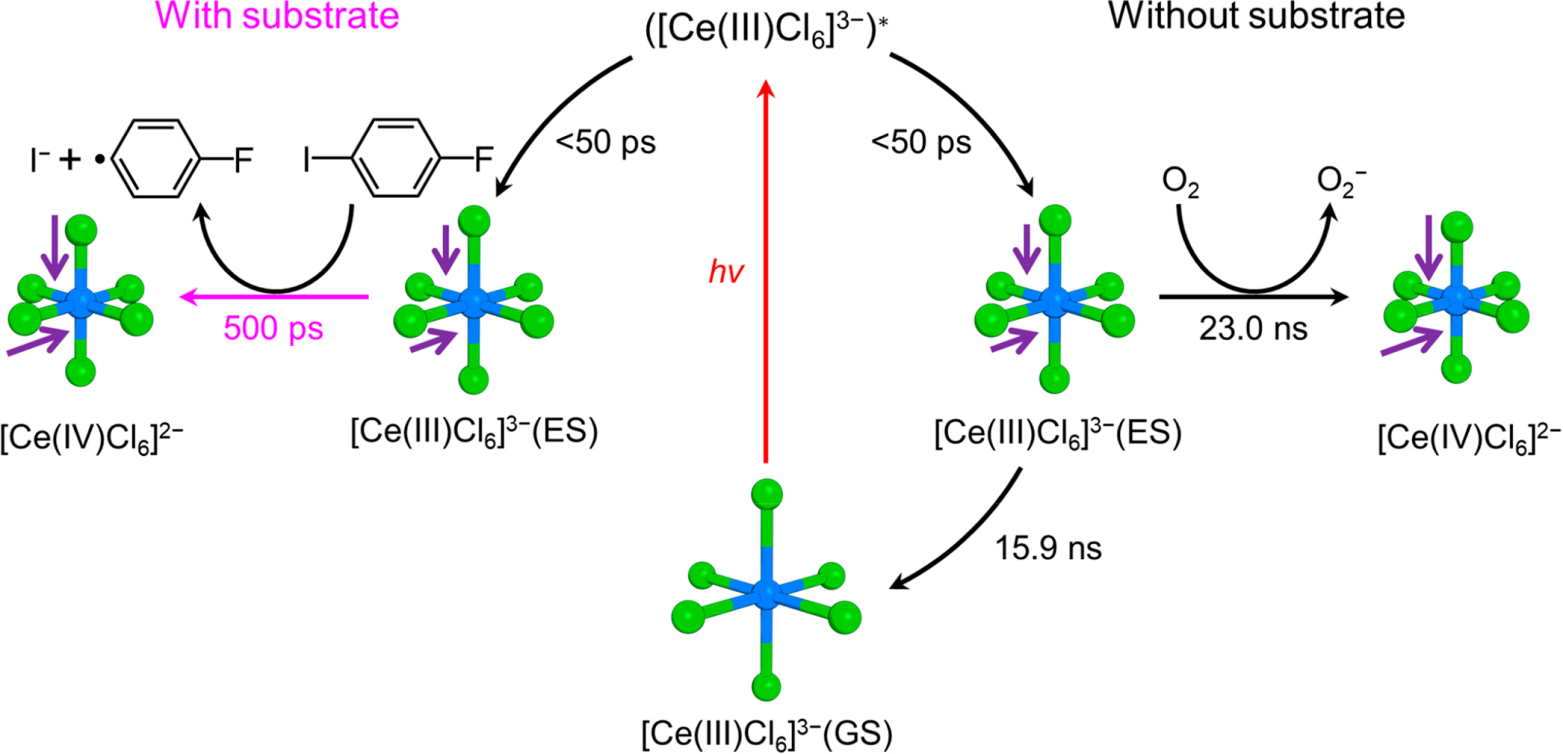
Schematic for the structural dynamics of [Ce(III)Cl_6_]^3–^ upon photoexcitation. Upon photoexcitation, [Ce(III)Cl_6_]^3–^(GS) is excited to the Franck–Condon region (([Ce(III)Cl_6_]^3–^)*), whose structure rapidly transforms into the stable structure of the excited state ([Ce(III)Cl_6_]^3–^(ES)) with a rate faster than our temporal resolution allows for observation. [Ce(III)Cl_6_]^3–^(ES) has Ce–Cl distances shorter than those of the ground state ([Ce(III)Cl_6_]^3–^(GS)). The reaction pathway of [Ce(III)Cl_6_]^3–^(ES) depends on the presence of the substrate (1-fluoro-4-iodobenzene). When the substrate is present, [Ce(III)Cl_6_]^3–^(ES) is oxidized through a C–I bond activation reaction with the substrate, resulting in the oxidized state ([Ce(IV)Cl_6_]^2–^) with further contracted Ce–Cl distances. In the absence of the substrate, a portion of [Ce(III)Cl_6_]^3–^(ES) is oxidized through a reaction with oxygen, similar to the case with the substrate. In the absence of a substrate, there are additional pathways. The remaining [Ce(III)Cl_6_]^3–^(ES) recovers to [Ce(III)Cl_6_]^3–^(GS) through either emission or reaction with oxygen. In the schematic, the cerium and chloride atoms are shown in blue and green, respectively. The lengths of the purple arrows in the molecular structures indicate the degrees of contraction of Ce–Cl bond lengths relative to [Ce(III)Cl_6_]^3–^(GS).

The schematics for the photoreaction of the [Ce(III)Cl_6_]^3–^/substrate and [Ce(III)Cl_6_]^3–^-only samples are shown in Figures 5 and S10. Upon photoexcitation, [Ce(III)Cl_6_]^3–^(GS) forms [Ce(III)Cl_6_]^3–^(ES) with shorter Ce–Cl bonds via transiently formed ([Ce(III)Cl_6_]^3–^)*. When the substrate is present, [Ce(III)Cl_6_]^3–^(ES) transforms into [Ce(IV)Cl_6_]^2–^ with further contraction of the Ce–Cl bonds through a reaction with the substrate. In the absence of the substrate, [Ce(III)Cl_6_]^3–^(ES) decays via two competing processes. Approximately 40% of [Ce(III)Cl_6_]^3–^(ES) undergoes an oxidation process to form [Ce(IV)Cl_6_]^2–^ as in the case when the substrate is present, but even without the presence of the substrate, through a reaction with oxygen. The remaining 60% decays to [Ce(III)Cl_6_]^3–^(GS) through the energy transfer to oxygen or emission.

## Conclusion

In conclusion, this study investigated the structural dynamics of [Ce(III)Cl_6_]^3–^, a Ce-containing photocatalyst, using TRXL and revealed the distinct structural dynamics both in the presence and absence of the substrate. The data analysis shows that the Ce–Cl bonds of [Ce(III)Cl_6_]^3–^ in the solution phase contract while maintaining octahedral symmetry upon photoexcitation. Furthermore, we experimentally determined the detailed molecular structure of the excited state as well as the ground and oxidized states. Considering that the excited state plays an important role as an active species for catalytic activity, it is important to experimentally unveil the molecular structure of the excited state. Additionally, we emphasize that this is the first study to reveal the structural dynamics depending on the presence of the substrate using TRXL. Despite that TRXL is an excellent experimental technique for studying the structural dynamics of photocatalysts, it has not been utilized to investigate the effect of the substrate on the dynamics of the photocatalysts. In this regard, this study should pave the way for employing TRXL to explore the structural dynamics of the photocatalysts.

## Supporting Information Available

Supplemental experimental procedures for TRXL and analysis methods including kinetic analysis, structure refinement, and *r*-space analysis; Figures S1–S15, Tables S1–S6, and supplemental references (PDF)

## ABBREVIATIONS

TRXL: time-resolved X-ray liquidography
PEPC: projection to extract the perpendicular component
SVD: singular value decomposition
LSV: left singular vector
RSV: right singular vector
TCSPC: time-correlated single photon counting
PCM: polarizable continuum model
C-PCM: conductor-like PCM
ZORA: zeroth-order regular approximation
DFT: density functional theory
TD-DFT: time-dependent DFT
SOMO: singly occupied molecular orbital
SADS: species-associated difference scattering curve

## References

[ref1] Mkhalid I. A. I.; Barnard J. H.; Marder T. B.; Murphy J. M.; Hartwig J. F. C–H Activation for the Construction of C–B Bonds. Chem. Rev. 2010, 110 (2), 890–931. 10.1021/cr900206p. 20028025

[ref2] Nagib D. A.; MacMillan D. W. C. Trifluoromethylation of arenes and heteroarenes by means of photoredox catalysis. Nature 2011, 480 (7376), 224–228. 10.1038/nature10647. 22158245 PMC3310175

[ref3] Prier C. K.; Rankic D. A.; MacMillan D. W. C. Visible Light Photoredox Catalysis with Transition Metal Complexes: Applications in Organic Synthesis. Chem. Rev. 2013, 113 (7), 5322–5363. 10.1021/cr300503r. 23509883 PMC4028850

[ref4] Park Y.; Kim Y.; Chang S. Transition Metal-Catalyzed C–H Amination: Scope, Mechanism, and Applications. Chem. Rev. 2017, 117 (13), 9247–9301. 10.1021/acs.chemrev.6b00644. 28051855

[ref5] Koike T.; Akita M. New Horizons of Photocatalytic Fluoromethylative Difunctionalization of Alkenes. Chem. 2018, 4 (3), 409–437. 10.1016/j.chempr.2017.11.004.

[ref6] Gontala A.; Jang G. S.; Woo S. K. Visible-Light Photoredox-Catalyzed α-Allylation of α-Bromocarbonyl Compounds Using Allyltrimethylsilane. Bull. Korean Chem. Soc. 2021, 42 (3), 506–509. 10.1002/bkcs.12219.

[ref7] Feng G.; Wang X.; Jin J. Decarboxylative C–C and C–N Bond Formation by Ligand-Accelerated Iron Photocatalysis. Eur. J. Org. Chem. 2019, 2019 (39), 6728–6732. 10.1002/ejoc.201901381.

[ref8] Xia S.; Hu K.; Lei C.; Jin J. Intramolecular Aromatic C–H Acyloxylation Enabled by Iron Photocatalysis. Org. Lett. 2020, 22 (4), 1385–1389. 10.1021/acs.orglett.0c00002. 31999131

[ref9] Ociepa M.; Wierzba A. J.; Turkowska J.; Gryko D. Polarity-Reversal Strategy for the Functionalization of Electrophilic Strained Molecules via Light-Driven Cobalt Catalysis. J. Am. Chem. Soc. 2020, 142 (11), 5355–5361. 10.1021/jacs.0c00245. 32105464

[ref10] Li Y.; Zhou K.; Wen Z.; Cao S.; Shen X.; Lei M.; Gong L. Copper(II)-Catalyzed Asymmetric Photoredox Reactions: Enantioselective Alkylation of Imines Driven by Visible Light. J. Am. Chem. Soc. 2018, 140 (46), 15850–15858. 10.1021/jacs.8b09251. 30372057

[ref11] Treacy S. M.; Rovis T. Copper Catalyzed C(sp3)–H Bond Alkylation via Photoinduced Ligand-to-Metal Charge Transfer. J. Am. Chem. Soc. 2021, 143 (7), 2729–2735. 10.1021/jacs.1c00687. 33576606 PMC8608032

[ref12] Reichle A.; Sterzel H.; Kreitmeier P.; Fayad R.; Castellano F. N.; Rehbein J.; Reiser O. Copper(ii)-photocatalyzed decarboxylative oxygenation of carboxylic acids. Chem. Commun. 2022, 58 (28), 4456–4459. 10.1039/D2CC00570K. 35201250

[ref13] Awwad N.; Bui A. T.; Danilov E. O.; Castellano F. N. Visible-Light-Initiated Free-Radical Polymerization by Homomolecular Triplet-Triplet Annihilation. Chem. 2020, 6 (11), 3071–3085. 10.1016/j.chempr.2020.08.019.

[ref14] Santos M. S.; Corrêa A. G.; Paixão M. W.; König B. C(sp3)–C(sp3) Cross-Coupling of Alkyl Bromides and Ethers Mediated by Metal and Visible Light Photoredox Catalysis. Adv. Synth. Catal. 2020, 362 (12), 2367–2372. 10.1002/adsc.202000167.

[ref15] Li J.; Huang C.-Y.; Li C.-J. Two-in-one metallaphotoredox cross-couplings enabled by a photoactive ligand. Chem. 2022, 8 (9), 2419–2431. 10.1016/j.chempr.2022.05.011.

[ref16] Yin H.; Carroll P. J.; Anna J. M.; Schelter E. J. Luminescent Ce(III) Complexes as Stoichiometric and Catalytic Photoreductants for Halogen Atom Abstraction Reactions. J. Am. Chem. Soc. 2015, 137 (29), 9234–9237. 10.1021/jacs.5b05411. 26151154

[ref17] Guo J.-J.; Hu A.; Chen Y.; Sun J.; Tang H.; Zuo Z. Photocatalytic C–C Bond Cleavage and Amination of Cycloalkanols by Cerium(III) Chloride Complex. Angew. Chem., Int. Ed. 2016, 55 (49), 15319–15322. 10.1002/anie.201609035. 27862775

[ref18] Yin H.; Carroll P. J.; Manor B. C.; Anna J. M.; Schelter E. J. Cerium Photosensitizers: Structure–Function Relationships and Applications in Photocatalytic Aryl Coupling Reactions. J. Am. Chem. Soc. 2016, 138 (18), 5984–5993. 10.1021/jacs.6b02248. 27058605

[ref19] Yin H.; Jin Y.; Hertzog J. E.; Mullane K. C.; Carroll P. J.; Manor B. C.; Anna J. M.; Schelter E. J. The Hexachlorocerate(III) Anion: A Potent, Benchtop Stable, and Readily Available Ultraviolet A Photosensitizer for Aryl Chlorides. J. Am. Chem. Soc. 2016, 138 (50), 16266–16273. 10.1021/jacs.6b05712. 27936638

[ref20] Hu A.; Guo J.-J.; Pan H.; Zuo Z. Selective functionalization of methane, ethane, and higher alkanes by cerium photocatalysis. Science 2018, 361 (6403), 668–672. 10.1126/science.aat9750. 30049785

[ref21] Schwarz J.; König B. Visible-light mediated C–C bond cleavage of 1,2-diols to carbonyls by cerium-photocatalysis. Chem. Commun. 2019, 55 (4), 486–488. 10.1039/C8CC09208G. 30548043

[ref22] An Q.; Wang Z.; Chen Y.; Wang X.; Zhang K.; Pan H.; Liu W.; Zuo Z. Cerium-Catalyzed C–H Functionalizations of Alkanes Utilizing Alcohols as Hydrogen Atom Transfer Agents. J. Am. Chem. Soc. 2020, 142 (13), 6216–6226. 10.1021/jacs.0c00212. 32181657

[ref23] Chen Y.; Du J.; Zuo Z. Selective C-C Bond Scission of Ketones via Visible-Light-Mediated Cerium Catalysis. Chem. 2020, 6 (1), 266–279. 10.1016/j.chempr.2019.11.009.

[ref24] Xu J.; Cai H.; Shen J.; Shen C.; Wu J.; Zhang P.; Liu X. Photo-Induced Cross-Dehydrogenative Alkylation of Heteroarenes with Alkanes under Aerobic Conditions. J. Org. Chem. 2021, 86 (24), 17816–17832. 10.1021/acs.joc.1c02125. 34875167

[ref25] Yang Q.; Wang Y.-H.; Qiao Y.; Gau M.; Carroll P. J.; Walsh P. J.; Schelter E. J. Photocatalytic C–H activation and the subtle role of chlorine radical complexation in reactivity. Science 2021, 372 (6544), 847–852. 10.1126/science.abd8408. 34016778

[ref26] Li H.-C.; Li G.-N.; Sun K.; Chen X.-L.; Jiang M.-X.; Qu L.-B.; Yu B. Ce(III)/Photoassisted Synthesis of Amides from Carboxylic Acids and Isocyanates. Org. Lett. 2022, 24 (12), 2431–2435. 10.1021/acs.orglett.2c00699. 35311287

[ref27] An Q.; Xing Y.-Y.; Pu R.; Jia M.; Chen Y.; Hu A.; Zhang S.-Q.; Yu N.; Du J.; Zhang Y.; Chen J.; Liu W.; Hong X.; Zuo Z. Identification of Alkoxy Radicals as Hydrogen Atom Transfer Agents in Ce-Catalyzed C–H Functionalization. J. Am. Chem. Soc. 2023, 145 (1), 359–376. 10.1021/jacs.2c10126. 36538367

[ref28] Ihee H. Visualizing Solution-Phase Reaction Dynamics with Time-Resolved X-ray Liquidography. Acc. Chem. Res. 2009, 42 (2), 356–366. 10.1021/ar800168v. 19117426

[ref29] Kim T. K.; Lee J. H.; Wulff M.; Kong Q.; Ihee H. Spatiotemporal Kinetics in Solution Studied by Time-Resolved X-Ray Liquidography (Solution Scattering). ChemPhysChem 2009, 10 (12), 1958–1980. 10.1002/cphc.200900154. 19585639

[ref30] Kim T. K.; Lorenc M.; Lee J. H.; Russo M. L.; Kim J.; Cammarata M.; Kong Q.; Noel S.; Plech A.; Wulff M.; Ihee H. Spatiotemporal reaction kinetics of an ultrafast photoreaction pathway visualized by time-resolved liquid x-ray diffraction. Proc. Natl. Acad. Sci. U.S.A. 2006, 103 (25), 9410–9415. 10.1073/pnas.0601958103. 16772380 PMC1478163

[ref31] Christensen M.; Haldrup K.; Bechgaard K.; Feidenhans’l R.; Kong Q.; Cammarata M.; Russo M. L.; Wulff M.; Harrit N.; Nielsen M. M. Time-Resolved X-ray Scattering of an Electronically Excited State in Solution. Structure of the 3A2u State of Tetrakis-μ-pyrophosphitodiplatinate(II). J. Am. Chem. Soc. 2009, 131 (2), 502–508. 10.1021/ja804485d. 19140790

[ref32] Kim K. H.; Kim J. G.; Nozawa S.; Sato T.; Oang K. Y.; Kim T. W.; Ki H.; Jo J.; Park S.; Song C.; Sato T.; Ogawa K.; Togashi T.; Tono K.; Yabashi M.; Ishikawa T.; Kim J.; Ryoo R.; Kim J.; Ihee H.; Adachi S.-i. Direct observation of bond formation in solution with femtosecond X-ray scattering. Nature 2015, 518 (7539), 385–389. 10.1038/nature14163. 25693570

[ref33] Canton S. E.; Kjær K. S.; Vankó G.; van Driel T. B.; Adachi S.-i.; Bordage A.; Bressler C.; Chabera P.; Christensen M.; Dohn A. O.; Galler A.; Gawelda W.; Gosztola D.; Haldrup K.; Harlang T.; Liu Y.; Møller K. B.; Németh Z.; Nozawa S.; Pápai M.; Sato T.; Sato T.; Suarez-Alcantara K.; Togashi T.; Tono K.; Uhlig J.; Vithanage D. A.; Wärnmark K.; Yabashi M.; Zhang J.; Sundström V.; Nielsen M. M. Visualizing the non-equilibrium dynamics of photoinduced intramolecular electron transfer with femtosecond X-ray pulses. Nat. Commun. 2015, 6 (1), 6359 10.1038/ncomms7359. 25727920 PMC4366532

[ref34] Biasin E.; van Driel T. B.; Kjær K. S.; Dohn A. O.; Christensen M.; Harlang T.; Vester P.; Chabera P.; Liu Y.; Uhlig J.; Pápai M.; Németh Z.; Hartsock R.; Liang W.; Zhang J.; Alonso-Mori R.; Chollet M.; Glownia J. M.; Nelson S.; Sokaras D.; Assefa T. A.; Britz A.; Galler A.; Gawelda W.; Bressler C.; Gaffney K. J.; Lemke H. T.; Møller K. B.; Nielsen M. M.; Sundström V.; Vankó G.; Wärnmark K.; Canton S. E.; Haldrup K. Femtosecond X-Ray Scattering Study of Ultrafast Photoinduced Structural Dynamics in Solvated [Co(terpy)_2_]^2+^. Phys. Rev. Lett. 2016, 117 (1), 013002 10.1103/PhysRevLett.117.013002. 27419566

[ref35] van Driel T. B.; Kjær K. S.; Hartsock R. W.; Dohn A. O.; Harlang T.; Chollet M.; Christensen M.; Gawelda W.; Henriksen N. E.; Kim J. G.; Haldrup K.; Kim K. H.; Ihee H.; Kim J.; Lemke H.; Sun Z.; Sundström V.; Zhang W.; Zhu D.; Møller K. B.; Nielsen M. M.; Gaffney K. J. Atomistic characterization of the active-site solvation dynamics of a model photocatalyst. Nat. Commun. 2016, 7 (1), 13678 10.1038/ncomms13678. 27892472 PMC5133712

[ref36] Choi E. H.; Ahn D.-S.; Park S.; Kim C.; Ahn C. W.; Kim S.; Choi M.; Yang C.; Kim T. W.; Ki H.; Choi J.; Pedersen M. N.; Wulff M.; Kim J.; Ihee H. Structural Dynamics of Bismuth Triiodide in Solution Triggered by Photoinduced Ligand-to-Metal Charge Transfer. J. Phys. Chem. Lett. 2019, 10 (6), 1279–1285. 10.1021/acs.jpclett.9b00365. 30835478

[ref37] Haldrup K.; Levi G.; Biasin E.; Vester P.; Laursen M. G.; Beyer F.; Kjær K. S.; Brandt van Driel T.; Harlang T.; Dohn A. O.; Hartsock R. J.; Nelson S.; Glownia J. M.; Lemke H. T.; Christensen M.; Gaffney K. J.; Henriksen N. E.; Møller K. B.; Nielsen M. M. Ultrafast X-Ray Scattering Measurements of Coherent Structural Dynamics on the Ground-State Potential Energy Surface of a Diplatinum Molecule. Phys. Rev. Lett. 2019, 122 (6), 063001 10.1103/PhysRevLett.122.063001. 30822093

[ref38] Kjær K. S.; Van Driel T. B.; Harlang T. C. B.; Kunnus K.; Biasin E.; Ledbetter K.; Hartsock R. W.; Reinhard M. E.; Koroidov S.; Li L.; Laursen M. G.; Hansen F. B.; Vester P.; Christensen M.; Haldrup K.; Nielsen M. M.; Dohn A. O.; Pápai M. I.; Møller K. B.; Chabera P.; Liu Y.; Tatsuno H.; Timm C.; Jarenmark M.; Uhlig J.; Sundstöm V.; Wärnmark K.; Persson P.; Németh Z.; Szemes D. S.; Bajnóczi É.; Vankó G.; Alonso-Mori R.; Glownia J. M.; Nelson S.; Sikorski M.; Sokaras D.; Canton S. E.; Lemke H. T.; Gaffney K. J. Finding intersections between electronic excited state potential energy surfaces with simultaneous ultrafast X-ray scattering and spectroscopy. Chem. Sci. 2019, 10 (22), 5749–5760. 10.1039/C8SC04023K. 31293761 PMC6568243

[ref39] Kong Q. Y.; Laursen M. G.; Haldrup K.; Kjær K. S.; Khakhulin D.; Biasin E.; van Driel T. B.; Wulff M.; Kabanova V.; Vuilleumier R.; Bratos S.; Nielsen M. M.; Gaffney K. J.; Weng T. C.; Koch M. H. J. Initial metal–metal bond breakage detected by fs X-ray scattering in the photolysis of Ru_3_(CO)_12_ in cyclohexane at 400 nm. Photochem. Photobiol. Sci. 2019, 18 (2), 319–327. 10.1039/c8pp00420j. 30628601

[ref40] Leshchev D.; Khakhulin D.; Newby G.; Ki H.; Ihee H.; Wulff M. Sub-nanosecond secondary geminate recombination in mercury halides HgX2 (X = I, Br) investigated by time-resolved x-ray scattering. J. Chem. Phys. 2019, 151 (5), 054310 10.1063/1.5096422.

[ref41] Kim J. G.; Nozawa S.; Kim H.; Choi E. H.; Sato T.; Kim T. W.; Kim K. H.; Ki H.; Kim J.; Choi M.; Lee Y.; Heo J.; Oang K. Y.; Ichiyanagi K.; Fukaya R.; Lee J. H.; Park J.; Eom I.; Chun S. H.; Kim S.; Kim M.; Katayama T.; Togashi T.; Owada S.; Yabashi M.; Lee S. J.; Lee S.; Ahn C. W.; Ahn D.-S.; Moon J.; Choi S.; Kim J.; Joo T.; Kim J.; Adachi S.-i.; Ihee H. Mapping the emergence of molecular vibrations mediating bond formation. Nature 2020, 582 (7813), 520–524. 10.1038/s41586-020-2417-3. 32581378

[ref42] Biasin E.; Fox Z. W.; Andersen A.; Ledbetter K.; Kjær K. S.; Alonso-Mori R.; Carlstad J. M.; Chollet M.; Gaynor J. D.; Glownia J. M.; Hong K.; Kroll T.; Lee J. H.; Liekhus-Schmaltz C.; Reinhard M.; Sokaras D.; Zhang Y.; Doumy G.; March A. M.; Southworth S. H.; Mukamel S.; Gaffney K. J.; Schoenlein R. W.; Govind N.; Cordones A. A.; Khalil M. Direct observation of coherent femtosecond solvent reorganization coupled to intramolecular electron transfer. Nat. Chem. 2021, 13 (4), 343–349. 10.1038/s41557-020-00629-3. 33589787

[ref43] Choi E. H.; Kim J. G.; Kim J.; Ki H.; Lee Y.; Lee S.; Yoon K.; Kim J.; Kim J.; Ihee H. Filming ultrafast roaming-mediated isomerization of bismuth triiodide in solution. Nat. Commun. 2021, 12 (1), 4732 10.1038/s41467-021-25070-z. 34354075 PMC8342516

[ref44] Ki H.; Kim T. W.; Moon J.; Kim J.; Lee Y.; Heo J.; Kim K. H.; Kong Q.; Khakhulin D.; Newby G.; Kim J.; Kim J.; Wulff M.; Ihee H. Photoactivation of triosmium dodecacarbonyl at 400 nm probed with time-resolved X-ray liquidography. Chem. Commun. 2022, 58 (53), 7380–7383. 10.1039/D2CC02438A. 35695475

[ref45] Leshchev D.; J. S. Valentine A.; Kim P.; Mills A. W.; Roy S.; Chakraborty A.; Biasin E.; Haldrup K.; Hsu D. J.; Kirschner M. S.; Rimmerman D.; Chollet M.; Glownia J. M.; van Driel T. B.; Castellano F. N.; Li X.; Chen L. X. Revealing Excited-State Trajectories on Potential Energy Surfaces with Atomic Resolution in Real Time. Angew. Chem., Int. Ed. 2023, 62 (28), e202304615 10.1002/anie.202304615. 37114904

[ref46] Ki H.; Gu J.; Cha Y.; Lee K. W.; Ihee H. Projection to extract the perpendicular component (PEPC) method for extracting kinetics from time-resolved data. Struct. Dyn. 2023, 10 (3), 034103 10.1063/4.0000189. 37388296 PMC10306411

[ref47] Vasudevan D.; Wendt H. Electroreduction of oxygen in aprotic media. J. Electroanal. Chem. 1995, 392 (1), 69–74. 10.1016/0022-0728(95)04044-O.

[ref48] Wilkinson F.; Abdel-Shafi A. A. Mechanism of Quenching of Triplet States by Oxygen: Biphenyl Derivatives in Acetonitrile. J. Phys. Chem. A 1997, 101 (30), 5509–5516. 10.1021/jp970706o.

[ref49] Andersson M.; Malmerberg E.; Westenhoff S.; Katona G.; Cammarata M.; Wöhri A. B.; Johansson L. C.; Ewald F.; Eklund M.; Wulff M.; Davidsson J.; Neutze R. Structural Dynamics of Light-Driven Proton Pumps. Structure 2009, 17 (9), 1265–1275. 10.1016/j.str.2009.07.007. 19748347

[ref50] Kim K. H.; Muniyappan S.; Oang K. Y.; Kim J. G.; Nozawa S.; Sato T.; Koshihara S.-y.; Henning R.; Kosheleva I.; Ki H.; Kim Y.; Kim T. W.; Kim J.; Adachi S.-i.; Ihee H. Direct Observation of Cooperative Protein Structural Dynamics of Homodimeric Hemoglobin from 100 ps to 10 ms with Pump–Probe X-ray Solution Scattering. J. Am. Chem. Soc. 2012, 134 (16), 7001–7008. 10.1021/ja210856v. 22494177 PMC3337689

[ref51] Oang K. Y.; Kim J. G.; Yang C.; Kim T. W.; Kim Y.; Kim K. H.; Kim J.; Ihee H. Conformational Substates of Myoglobin Intermediate Resolved by Picosecond X-ray Solution Scattering. J. Phys. Chem. Lett. 2014, 5 (5), 804–808. 10.1021/jz4027425. 24761190 PMC3985870

[ref52] Rimmerman D.; Leshchev D.; Hsu D. J.; Hong J.; Kosheleva I.; Chen L. X. Direct Observation of Insulin Association Dynamics with Time-Resolved X-ray Scattering. J. Phys. Chem. Lett. 2017, 8 (18), 4413–4418. 10.1021/acs.jpclett.7b01720. 28853898 PMC5804350

[ref53] Lee Y.; Kim J. G.; Lee S. J.; Muniyappan S.; Kim T. W.; Ki H.; Kim H.; Jo J.; Yun S. R.; Lee H.; Lee K. W.; Kim S. O.; Cammarata M.; Ihee H. Ultrafast coherent motion and helix rearrangement of homodimeric hemoglobin visualized with femtosecond X-ray solution scattering. Nat. Commun. 2021, 12 (1), 3677 10.1038/s41467-021-23947-7. 34135339 PMC8209046

[ref54] Jeong H.; Ki H.; Kim J. G.; Kim J.; Lee Y.; Ihee H. Sensitivity of time-resolved diffraction data to changes in internuclear distances and atomic positions. Bull. Korean Chem. Soc. 2022, 43 (3), 376–390. 10.1002/bkcs.12494.

[ref55] Löble M. W.; Keith J. M.; Altman A. B.; Stieber S. C. E.; Batista E. R.; Boland K. S.; Conradson S. D.; Clark D. L.; Lezama Pacheco J.; Kozimor S. A.; Martin R. L.; Minasian S. G.; Olson A. C.; Scott B. L.; Shuh D. K.; Tyliszczak T.; Wilkerson M. P.; Zehnder R. A. Covalency in Lanthanides. An X-ray Absorption Spectroscopy and Density Functional Theory Study of LnCl6^x–^ (x = 3, 2). J. Am. Chem. Soc. 2015, 137 (7), 2506–2523. 10.1021/ja510067v. 25689484

[ref56] Barandiarán Z.; Edelstein N. M.; Ordejón B.; Ruipérez F.; Seijo L. Bond lengths of 4f^1^ and 5d^1^ states of Ce^3+^ hexahalides. J. Solid State Chem. 2005, 178 (2), 464–469. 10.1016/j.jssc.2004.09.020.

[ref57] Beekmeyer R.; Kerridge A. Assessing Covalency in Cerium and Uranium Hexachlorides: A Correlated Wavefunction and Density Functional Theory Study. Inorganics 2015, 3, 482–499. [Online] 10.3390/inorganics3040482.

[ref58] Tanner P. A.; Mak C. S. K.; Edelstein N. M.; Murdoch K. M.; Liu G.; Huang J.; Seijo L.; Barandiarán Z. Absorption and Emission Spectra of Ce^3+^ in Elpasolite Lattices. J. Am. Chem. Soc. 2003, 125 (43), 13225–13233. 10.1021/ja036659x. 14570498

[ref59] Valiente R.; Rodríguez F.; González J.; Güdel H. U.; Martín-Rodríguez R.; Nataf L.; Sanz-Ortiz M. N.; Krämer K. High pressure optical spectroscopy of Ce^3+^-doped Cs_2_NaLuCl_6_. Chem. Phys. Lett. 2009, 481 (1), 149–151. 10.1016/j.cplett.2009.09.059.

[ref60] Barandiarán Z.; Seijo L. Quantum chemical analysis of the bond lengths in f^n^ and f^n–1^d^1^ states of Ce^3+^, Pr^3+^, Pa^4+^, and U^4+^ defects in chloride hosts. J. Chem. Phys. 2003, 119 (7), 3785–3790. 10.1063/1.1590952.

[ref61] Ruipérez F.; Seijo L.; Barandiarán Z. Prediction of pressure-induced redshift of f^1^→d(t_2g_)^1^ excitations in Cs_2_NaYCl_6_:Ce^3+^ and its connection with bond-length shortening. J. Chem. Phys. 2005, 122 (23), 234507 10.1063/1.1935512. 16008462

[ref62] Aravena D.; Atanasov M.; Neese F. Periodic Trends in Lanthanide Compounds through the Eyes of Multireference ab Initio Theory. Inorg. Chem. 2016, 55 (9), 4457–4469. 10.1021/acs.inorgchem.6b00244. 27054547

