# Supplementary material for: Cerium Photocatalyst in Action: Structural Dynamics in the Presence of Substrate Visualized via Time-Resolved X-ray Liquidography
Source: J Am Chem Soc. 2023 Oct 19;145(43):23715–26. doi: 10.1021/jacs.3c08166 (PMC10623567; doi:10.1021/jacs.3c08166)
Supplement: Supplementary file 1 — ja3c08166_si_001.pdf [file ja3c08166_si_001.pdf]

Supporting Information for

Cerium Photocatalyst in Action: Structural Dynamics in the Presence of Substrate Visualized via Time-Resolved X-ray Liquidography

Yunbeom Lee,[†,‡] Hosung Ki,[†,‡] Donghwan Im,[†,‡] Seunghwan Eom,[†,‡] Jain Gu,[†,‡] Seonggon Lee,[†,‡] Jungmin Kim,[†,‡] Yongjun Cha,[†,‡] Kyung Won Lee,[†,‡] Serhane Zerdane,[§] Matteo Levantino,[§] and Hyotcherl Ihee[*,†,‡]

[†]Center for Advanced Reaction Dynamics, Institute for Basic Science (IBS), Daejeon, 34141, Republic of Korea

[‡]Department of Chemistry and KI for the BioCentury, Korea Advanced Institute of Science and Technology (KAIST), Daejeon, 34141, Republic of Korea

[§]European Synchrotron Radiation Facility (ESRF), 71 Avenue des Martyrs, 38000 Grenoble, France

*Corresponding author. E-mail: hyotcherl.ihee@kaist.ac.kr

The PDF file includes:

Materials and Methods

Figures S1 to S15

Tables S1 to S6

References

Materials and Methods

General procedure

All the reagents including the solvent were purchased from commercial sources. The reagents were used for the experiments without further purification.

Spectroscopic characterization

The UV-visible absorption spectrum of $[Ce(III)Cl_6]^{3-}$ was measured using UV-visible spectrometers (Agilent Cary5000 and Shimadzu UV-2600) on a solution of $[(C_2H_5)_4N]_3[Ce(III)Cl_6]$ and $[(C_2H_5)_4N]Cl$ dissolved in acetonitrile with concentrations of 0.25 mM and 150 mM, respectively. For the absorption measurement on this sample, a quartz cell with a thickness of 1 cm was used. UV-visible absorption spectrum of the sample with the substrate, 1-fluoro-4-iodobezene, was measured using an acetonitrile solution with concentrations of 2 mM, 150 mM, and 150 mM for $[(C_2H_5)_4N]_3[Ce(III)Cl_6]$, $[(C_2H_5)_4N]Cl$, and 1-fluoro-4-iodobenzene (F–Ph–I), respectively. The measurement of this sample was conducted in a quartz cell with a 1 mm path length. The emission of $[Ce(III)Cl_6]^{3-}$ was measured with the excitation wavelength of 340 nm using a fluorometer (HORIBA FluoroMax PLUS) on a solution with the same condition used for UV-visible absorbance measurement without substrate and LED irradiation (0.25 mM for $[Ce(III)Cl_6]^{3-}$ and 150 mM for $[(C_2H_5)_4N]Cl$). For the emission measurement, a quartz cell with a thickness of 1 cm was used. The lifetimes of the emission of $[Ce(III)Cl_6]^{3-}$ were measured using time-correlated single photon counting (TCSPC) technique (Hamamatsu Photonics K.K., Quantaurus-Tau). For TCSPC measurement, the same sample condition used for the emission measurement was used. In addition, we performed TCSPC measured for other samples in which the substrate was added at concentrations of 15, 30, 45, and 60 mM, while the concentrations of other compounds remained unchanged (0.25 mM for $[Ce(III)Cl_6]^{3-}$ and 150 mM for $[(C_2H_5)_4N]Cl$), to estimate the emission lifetime of the sample with the substrate concentration of 150 mM, which was the concentration used for the TRXL experiment. An optical pulse from a pulsed LED with a wavelength of 340 nm was used for the excitation of the sample. The emission at 390 nm was measured to determine the emission lifetime. The results of the spectroscopic characterization are shown in Figure S4.

Time-resolved X-ray liquidography

The TRXL experiments were performed at the ID09 beamline of the European Synchrotron Radiation Facility (ESRF). A typical experimental setup for the pump-probe scheme was used for the experiment. A laser pulse with a wavelength of ~330 nm, which was converted from the fundamental laser pulse of a Ti:sapphire laser (800 nm) via an optical parametric amplifier (OPA), was used to initiate the photoreaction. After a time delay, the X-ray pulse with the center energy of ~18 keV and energy bandwidth ($\Delta E/E$) of ~2.5% interrogated the structural change of molecules during the photoreaction. At the sample position, the laser and X-ray pulses were focused to the size of ~100 × 100 μm^2 and ~25 × 25 μm^2 (FWHM), respectively, and the laser fluence at the sample position was ~0.63 mJ/mm^2. The X-ray scattering signal from the sample was collected using an area detector (Rayonix MX170-HS) at a negative time delay (-3 ns) for reference and multiple positive time delays (50 ps, 100 ps, 215 ps, 464 ps, 1 ns, 2.15 ns, 4.64 ns, 10 ns, 21.5 ns, 46.4 ns, 100 ns, 215 ns, 464 ns, and 1 μs). The sample-to-detector distance was 49.8 mm. The X-ray scattering images were azimuthally integrated as a function of the magnitude of the momentum transfer vector (q) to generate 1D X-ray scattering curves. The X-ray scattering curves were

normalized, and the reference X-ray scattering curves were subtracted from the X-ray scattering curves collected at positive time delays to generate the difference X-ray scattering curves. The q range used for normalization was 4–7.5 Å^{-1}. The temperature of the experimental hutch was ~23 °C. To investigate the effect of the substrate on the structural dynamics of the catalyst, the TRXL experiments were performed on two sample solutions. One was the solution with the catalyst but without the substrate ($[Ce(III)Cl_6]^{3-}$-only sample), and the other was the solution with both the catalyst and substrate ($[Ce(III)Cl_6]^{3-}$/substrate sample). In the $[Ce(III)Cl_6]^{3-}$-only sample, $[(C_2H_5)_4N]_3[Ce(III)Cl_6]$ and $[(C_2H_5)_4N]Cl$ with the concentrations of 15 mM and 150 mM, respectively, were dissolved in acetonitrile. In the $[Ce(III)Cl_6]^{3-}$/substrate sample, which contained both the catalyst and substrate, $[(C_2H_5)_4N]_3[Ce(III)Cl_6]$, $[(C_2H_5)_4N]Cl$, 1-fluoro-4-iodobenzene (FC_6H_4I), and toluene ($C_6H_5CH_3$) were dissolved in acetonitrile with concentrations of 15 mM, 150 mM, 150 mM, and 150 mM, respectively.

The TRXL data show that not all photoexcited solute molecules returned to their ground state within our covered time delay range (up to 10 ns for the $[Ce(III)Cl_6]^{3-}$/substrate and 1 μs $[Ce(III)Cl_6]^{3-}$-only samples. See Figure 2). The oscillatory feature in the high-q region is distinctly noticeable, persisting even at the latest time delays, which indicates that some intermediates or products exist even at the latest measured time delay. This observation raises concerns about the possibility of data contamination if a solution containing these long-lived species recirculates and experiences additional excitation by optical laser pulses. Such a scenario could potentially introduce contaminated signals arising from the additional, undesirable reactions of these long-lived species. To mitigate this concern, we continuously monitored the shape of the difference scattering curve throughout the experiment. If such contamination were to occur, it would gradually alter the shape of the difference scattering curve over time when compared to that of a fresh sample. We confirmed that the shape of the difference scattering curve remained consistent during the measurement and did not exhibit any significant changes over the measurements. This consistency underscores the integrity of our sample and removes potential contamination concerns in the collected TRXL data.

The TRXL signals corresponding to the change of the bulk solvent upon heating, $(\partial S/\partial T)_\rho$ and $(\partial S/\partial \rho)_T$, were obtained from a separate experiment. For the measurement of the solvent heating signal, the acetonitrile solution of 4-bromo-4′-(*N*,*N*-diethylamino)-azobenzene, which is a typical dye used for the solvent heating experiment, with a concentration of 2.1 mM was used. The dye solution was excited by a laser pulse of the same wavelength as used for the excitation of the sample solutions, ~330 nm. After photoexcitation of the dye, the excited dye molecules dissipate energy to the solvent in the form of heat, leading to changes in solvent temperature and density. The TRXL signal due to such changes was measured. The contribution of structural changes in the photoexcited dye molecules themselves to the TRXL signal is known to be negligible.

The total sample volume used for the TRXL experiment was either 200 ml or 500 ml, and samples were periodically replaced (within no more than 7 hours for 200 ml samples and 16 hours for 500 ml samples, respectively). Considering the laser pulse intensity and wavelength used in the experiment, each laser pulse contained approximately 2.76×10^{-11} moles of photons. The absorbance of the sample was approximately 0.6, which indicates that approximately 2.08×10^{-11} moles of photocatalyst were excited by a laser pulse. According to our analysis, the quantum yield of $[Ce(III)Cl_6]^{3-}$/substrate samples is around 27%. Therefore, roughly 5.7×10^{-12} moles undergo the oxidation reaction per laser pulse. Taking into account a laser repetition rate of approximately 1000 Hz, through measurements conducted over 7 hours (for 200 ml samples) or 16 hours (for 500 ml samples), the fraction of the photocatalyst undergoing the reaction is estimated to be less than 5% of the total amount. The

sacrificial reagent is consumed through its reaction with the oxidized form of the photocatalyst ($[Ce(IV)Cl_6]^{2-}$). However, considering that the amount of the oxidized form generated in the experiment is small and the photoexcitation of the oxidation is required for the reaction between the oxidized form and sacrificial reagent, the consumption of the sacrificial reagent would be minor. These estimations suggest that the samples remained nearly intact throughout the experiment.

Kinetic analysis

The TRXL signals are composed of three components: (i) the signal due to the structural change of the solute itself (solute term), (ii) the signal due to the change in the arrangement of solvent molecules surrounding the solute molecules (cage term), and (iii) the signal due to the bulk solvent upon heating (solvent term). Typically, the kinetics of the first two terms (solute and cage terms) directly reflect the kinetics of the solute molecules, whereas the last term (solvent term) can have different kinetics from those of the solute molecules. In this regard, to analyze the kinetics of the solute molecules, which are of interest, it would be convenient to remove the contribution of the solvent term from the TRXL signals. For such a purpose, the projection to extract the perpendicular component (PEPC) method, which was recently developed by our group, was used.[1] In this procedure, the heating signals ($(\partial S/\partial T)_\rho$ and $(\partial S/\partial \rho)_T$ terms) from dye solution and an artifact component due to high laser fluence were used as the known components for PEPC and the kinetics related to the known components were removed from the experimental data. Afterward, singular value decomposition (SVD) and kinetics-constrained analysis (KCA) were performed on the PEPC-treated data to analyze the kinetics of the solute molecules. Using SVD, the PEPC-treated data was decomposed into left singular vectors (LSVs), right singular vectors (RSVs), and singular values. The LSVs show the time-independent difference scattering signals, the RSVs represent the time profile of the LSVs, and the singular values indicate the contribution of the singular vectors to the data. Figure S2 shows the LSVs, RSVs, singular values, and autocorrelation values obtained from the SVD analysis on the PEPC-treated TRXL data for the $[Ce(III)Cl_6]^{3-}$/substrate and $[Ce(III)Cl_6]^{3-}$-only samples, respectively. The results show that only one signal component contributes significantly to the data for both samples. To extract the time constant, the 1st RSV was fitted with a sum of a constant and an exponential function, yielding a time constant of 500 ± 20 ps for the $[Ce(III)Cl_6]^{3-}$/substrate sample and a time constant of 9.4 ± 0.3 ns for the $[Ce(III)Cl_6]^{3-}$-only sample. The time constants for the $[Ce(III)Cl_6]^{3-}$/substrate and $[Ce(III)Cl_6]^{3-}$-only samples obtained by TRXL agree with those measured and estimated by TCSPC. In the absence of substrate, an emission lifetime of 10.02 ns was observed under the ambient conditions with oxygen, closely resembling the time constant of the $[Ce(III)Cl_6]^{3-}$-only data. Meanwhile, in a sample with a substrate concentration of 150 mM was estimated from the emission lifetimes of multiple samples with different substrate concentrations (Figure S4C). From the emission lifetimes of these samples, the pseudo-first order rate constant for each sample was obtained using the following equation.

$$\frac{1}{\tau_{\mathrm{obs}}} = \frac{1}{\tau_{\mathrm{nosub}}} + k_{\mathrm{sub}} \tag{S1}$$

Here, τ_{obs} is the observed time constant under the presence of the substrate, τ_{nosub} is the time constant of the photocatalyst without substrate under the ambient conditions (10.02 ns), and k_{sub} is the pseudo-first order rate constant for the reaction between the photocatalyst and substrate. Then, the bimolecular rate constant (k_r) was

estimated by fitting the k_{sub}s with a linear function of the substrate concentration, as k_{sub} can be expressed using k_r as follows (Figure S4D).

$$k_{sub} = k_r[FPhI] \tag{S2}$$

Here, [FPhI] is the concentration of the substrate (1-fluoro-4-iodobenzene). Finally, the time constant (τ_{obs}) under the substrate concentration of 150 mM, which was the concentration used for the TRXL experiment, was estimated using k_r. The estimated time constant was ~700 ps. This time constant is comparable to 500 ps, the time constant from the TRXL data of the $[Ce(III)Cl_6]^{3-}$/substrate sample. These agreements indicate that $[Ce(III)Cl_6]^{3-}$(ES) can decay through reactions with 1-fluoro-4-iodobenzene and oxygen.

It is worth noting that even at the latest time delays (10 ns for the $[Ce(III)Cl_6]^{3-}$/substrate sample and 1 μs for the $[Ce(III)Cl_6]^{3-}$-only sample), the oscillatory feature in the high q region remains visible in both samples. Given that these time delays are significantly longer than the observed time constants of 500 ps and 9.4 ns, it is more likely that the processes corresponding to the observed time constants are not simple recovery to the ground state but involve the transition from one species to another new species. In principle, there should be two significant signal components in the result of SVD analysis if there are two distinct species. In reality, only one significant component was observed, indicating that although the amplitude of the difference scattering curves corresponding to the 1st and 2nd species may differ, their shapes are very similar. Even so, if the signal-to-noise ratio (SNR) of the measured data was much higher, two components would have been obtained. In other words, we attribute the absence of the second component to the insufficient SNR of the data.

Based on the result of the kinetic analysis of the 1st RSV, we extracted the PEPC-treated species-associated difference scattering curve (SADS) in q-space, $qA(q)^{\perp}$, for each species from the PEPC-treated data via KCA. In the first step, we used the obtained time constants to construct a simple sequential kinetic model consisting of two species and a time constant (See Figure S3). In the model, the first species is formed within the earliest measured time delay of 50 ps, and subsequently transforms to the second species with the observed time constant, 500 ps in the model for the $[Ce(III)Cl_6]^{3-}$/substrate sample and 9.4 ns in the model for the $[Ce(III)Cl_6]^{3-}$-only sample. The second species persists up to the latest measured time delays of 10 ns and 1 μs for the $[Ce(III)Cl_6]^{3-}$/substrate and $[Ce(III)Cl_6]^{3-}$-only samples, respectively.

In the model, the rate equations for the 1st and 2nd species of the $[Ce(III)Cl_6]^{3-}$/substrate and $[Ce(III)Cl_6]^{3-}$-only samples can be mathematically represented as follows:

$$\frac{d[SP_1]}{dt} = -\frac{[SP_1]}{\tau}dt \tag{S3}$$

$$\frac{d[SP_2]}{dt} = \frac{[SP_1]}{\tau}dt \tag{S4}$$

, where $[SP_1]$ and $[SP_2]$ indicate the relative concentrations of the 1st and 2nd species, and τ refers to the time constant obtained from the SVD analysis.

As mentioned in the main text, we assigned that the 1st species, the excited state ($[Ce(III)Cl_6]^{3-}$(ES)), oxidizes to form $[Ce(IV)Cl_6]^{2-}$ in both the $[Ce(III)Cl_6]^{3-}$/substrate and $[Ce(III)Cl_6]^{3-}$-only samples. One intriguing aspect is that this oxidation reaction occurs even in the $[Ce(III)Cl_6]^{3-}$-only sample, in the absence of the substrate. To support this assignment, we performed additional experiments using an LED light source. Specifically, we prepared the $[Ce(III)Cl_6]^{3-}$-only sample and irradiated the sample using the LED with a peak wavelength ~340

nm under ambient conditions. We measured the UV-Visible absorption spectra of the sample after irradiating the sample for 1 hour, confirming the growth of an absorption peak corresponding to $[Ce(IV)Cl_6]^{2-}$ at around 375 nm (Figure S4A).[2-3] This observation indicates that $[Ce(IV)Cl_6]^{2-}$ is formed upon photoexcitation under air even without the presence of the substrate. This observation can be supported simply by comparing the reduction potentials of $[Ce(III)Cl_6]^{3-}$(ES) and oxygen. The reduction potentials of $[Ce(III)Cl_6]^{3-}$(ES) and oxygen is -3.45 V and -1.3 V (vs. $Cp_2Fe^{0/+}$, where Cp_2Fe is ferrocene), respectively, as mentioned in the main text.[3-4] The oxidation potential of $[Ce(III)Cl_6]^{3-}$(ES) is more negative than oxygen, indicating that the oxidation of the photocatalyst by oxygen is natural.

For the reaction observed in $[Ce(III)Cl_6]^{3-}$-only sample, the oxidation of a fraction of $[Ce(III)Cl_6]^{3-}$(ES) to $[Ce(IV)Cl_6]^{2-}$ with a time constant of 9.4 ns, we investigated more detailed mechanistic insight into this process. The structural analysis of the 2nd species for the $[Ce(III)Cl_6]^{3-}$-only sample reveals that only a fraction (about 41%) of the 1st species undergoes the oxidation reaction. This observation indicates that the remaining fraction of the 1st species undergoes recovery to the ground state ($[Ce(III)Cl_6]^{3-}$(GS)). We suggest that the recovery process, where $[Ce(III)Cl_6]^{3-}$(ES) returns to $[Ce(III)Cl_6]^{3-}$(GS), occurs through two competitive pathways: emissive decay to $[Ce(III)Cl_6]^{3-}$(GS) and energy transfer to oxygen. The basis for considering these intricate reaction pathways is not solely derived from our TRXL measurement but is primarily supported by other experimental measurements, particularly the TCSPC measurements conducted under both inert atmosphere and air conditions (Figure S4B).

The initial motivation of the TCSPC measurement was to determine whether the process, recovery of $[Ce(III)Cl_6]^{3-}$(ES) to $[Ce(III)Cl_6]^{3-}$(GS) is emissive or not. We measured TCSPC and obtained clear decay profiles, confirming that there is a contribution of the emissive pathway. One noteworthy observation is that the lifetime of $[Ce(III)Cl_6]^{3-}$(ES) measured under ambient conditions (10.02 ± 0.02 ns) differs considerably from that measured under inert conditions (25.07 ± 0.04 ns), indicating the presence of a pathway that competes with radiative decay in the presence of oxygen. Initially, we speculated that this discrepancy could be attributed to an oxidation reaction induced by oxygen. Consequently, we established the corresponding kinetic model and conducted data analysis, but unfortunately, the model failed to satisfactorily fit our TRXL data (not shown here).

The main reason for the model's inadequacy, and the unsatisfactory fitting of the TRXL data based on the model, is the significantly higher estimated yield of $[Ce(IV)Cl_6]^{2-}$ formation (about 63%) compared to the actual yield obtained from the analysis of the TRXL data (about 41%). The underlying cause of this discrepancy can be explained as follows: Assuming two possible pathways, oxidation and radiative decay, and fixing the radiative decay rate at 25.07 ns, which was obtained from TCSPC under inert conditions, we adjusted the rate of the oxidation pathway to achieve an apparent decay time constant matching the observed value of 9.4 ns in the TRXL data. The time constant for the oxidation pathway, about 15.04 ns, was determined through this adjustment. Given the competition between the oxidation pathway with a time constant of 15.04 ns and the radiative decay with a time constant of 25.07 ns, the branching ratio of these two reactions is inversely proportional to their respective time constants, leading to a ratio of 1/15.04:1/25.07 = 63:37. However, this predicted oxidation yield of 63% deviates significantly from the experimentally observed yield of 41%.

As a result, it becomes evident that the model considering the two pathways, oxidation and radiative decay, is insufficient to fully explain the experimental data. Upon observing this discrepancy, we formulated a hypothesis suggesting the existence of additional pathways for the recovery of $[Ce(III)Cl_6]^{3-}$(ES) to

$[Ce(III)Cl_6]^{3-}$(GS), beyond the process of radiative decay. We considered energy transfer to oxygen as a promising candidate for such an additional pathway. The reason for choosing this pathway as the candidate is based on energetic considerations, as mentioned in the main text. The energy transfer from the photocatalyst to oxygen is feasible as the energy gap between the first excited state and the ground state of oxygen (~0.97 eV) is smaller than that of the photocatalyst (~3.5 eV) obtained from the emission spectrum.[5] With the incorporation of all three pathways, namely oxidation by oxygen, radiative decay to $[Ce(III)Cl_6]^{3-}$(GS), and energy transfer to oxygen, into the model, we were finally able to satisfactorily fit our experimental data.

We note that there is a discrepancy between the time constant obtained from TRXL for the ground-state recovery (15.9 ns, Figure 5) and the constant obtained from TCSPC (10.02 ns, Figure S4B). Regarding the origin of the discrepancy, it is important to clarify that these time constants do not correspond to the same process. The emission intensity of the excited state would decrease if the population of the excited state decreases, regardless of the specific process the excited state undergoes. For instance, the decrease in emission intensity would occur both when the excited state transforms into the oxidized state and when the excited state recovers to the ground state. If there are two or more competing processes that decay the population of the excited state, the apparent rate of emission decay observed in the TCSPC would not represent the specific rate of either process but rather represent the sum of the rates of the competing processes. Accordingly, the rate observed in TCSPC is faster than that of each individual process. This is the reason why the time constant observed in TCSPC (10.02 ns) is faster than the time constant observed in TRXL (15.9 ns). In TRXL, the observed time constant is for a specific process, which is the recovery of $[Ce(III)Cl_6]^{3-}$(ES) to $[Ce(III)Cl_6]^{3-}$(GS). On the other hand, TCSPC observes the sum of the rate of two different processes, the recovery of $[Ce(III)Cl_6]^{3-}$(ES) to $[Ce(III)Cl_6]^{3-}$(GS) and the "oxidation" of $[Ce(III)Cl_6]^{3-}$(ES) to $[Ce(IV)Cl_6]^{2-}$. Figure S10 provides further insights into this point. The apparent time constant of 15.9 ns observed in TRXL corresponds to the sum of the time constants for the two pathways, energy transfer to oxygen (43.5 ns) and emissive decay to $[Ce(III)Cl_6]^{3-}$(GS) (25.1 ns), for a specific process, the recovery of $[Ce(III)Cl_6]^{3-}$(ES) to $[Ce(III)Cl_6]^{3-}$(GS). On the other hand, the observed TCSPC time constant of 10.02 ns includes the contribution from the oxidation process by oxygen (23.0 ns) in addition to the energy transfer to oxygen and emissive decay to $[Ce(III)Cl_6]^{3-}$(GS). These combined processes result in an apparent time constant of approximately 9.4 ns, which is similar to the 10.02 ns observed in TCSPC.

We used the constructed kinetic model to determine the time-dependent relative contributions of each species. Based on these calculated contributions, we performed KCA to extract $q\mathrm{A}(q)^{\perp}$s from the PEPC-treated data. The underlying principle for KCA can be explained using mathematical equations as follows. The PEPC-treated data can be expressed as a combination of each species' $q\mathrm{A}(q)^{\perp}$ and their corresponding relative contributions, as follows:

$$\Delta\mathrm{S}(q,\ t)^{\perp} = \sum_i \mathrm{A}_i(q)^{\perp} \cdot c_i(t) \tag{S5}$$

, where $\Delta\mathrm{S}(q, t)^{\perp}$ is the PEPC-treated data, $\mathrm{A}_i(q)^{\perp}$ is the PEPC-treated SADS in q-space for the ith species, and $c_i(t)$ is the relative contribution of the ith species at the time delay t. For a more detailed explanation, we can express this equation in matrix form as follows:

$$\begin{aligned}\Delta S(q,\ t)^{\perp} &= [\Delta S(q,\ t_1)^{\perp}, \Delta S(q,\ t_2)^{\perp}, \cdots, \Delta S(q,\ t_{n_t})^{\perp}] \\ &= [\sum_i A_i(q)^{\perp} \cdot c_i(t_1), \sum_i A_i(q)^{\perp} \cdot c_i(t_2), \cdots, \sum_i A_i(q)^{\perp} \cdot c_i(t_{n_t})] \\ &= [A_1(q)^{\perp}, A_2(q)^{\perp}, \cdots, A_{n_s}(q)^{\perp}][c_1(t), c_2(t), \cdots, c_{n_s}(t)]^{T} \\ &= M(q)C(t)^{T}\end{aligned} \tag{S6}$$

, where n_s is the number of species (in this case, two), $M(q)$ is a matrix of the $A(q)^{\perp}$s appearing on the third line of Equation S6, and $C(t)$ is a matrix of the time profiles of the relative contribution shown on the same line. The symbol X^T denotes the transpose of matrix X. Transposing Equation S6 results in the following equation in the well-known form of AX = B.

$$C(t)M(q)^{T} = (\Delta S(q,\ t)^{\perp})^{T} \tag{S7}$$

The $qA(q)^{\perp}$s, which are obtained via multiplying $A(q)^{\perp}$s by q, can be obtained by solving Equation S7 for $M(q)$. Since the other two terms, $C(t)$ and $\Delta S(q,\ t)^{\perp}$, are already known, the method for solving Equation S7 is straightforward and is explained in detail as follows. To calculate $M(q)$, we calculated the Moore-Penrose inverse, in other words, pseudoinverse, of the matrix $C(t)$ and multiplied its transpose to $\Delta S(q,\ t)^{\perp}$. The pseudoinverse is mathematically expressed as follows.

$$C(t)^{+} = (C(t)^{T} C(t))^{-1} C(t)^{T} \tag{S8}$$

With the pseudoinverse of $C(t)$, $qA(q)^{\perp}$s can be obtained by using the following equation.

$$\begin{aligned}[A_1(q)^{\perp}, A_2(q)^{\perp}, \cdots, A_{n_s}(q)^{\perp}] = M(q) &= \{C(t)^{+}(\Delta S(q,\ t)^{\perp})^{T}\}^{T} \\ &= \Delta S(q,\ t)^{\perp}(C(t)^{+})^{T}\end{aligned} \tag{S9}$$

By analyzing the $qA(q)^{\perp}$s obtained through the KCA, we extracted the detailed structural information for each species. The details of the structural analysis of the obtained $qA(q)^{\perp}$s are described in the following "Structure refinement" section.

The validity of the kinetic model and its corresponding $qA(q)^{\perp}$s in describing the experimental data can be assessed employing linear combination fitting (LCF). First, we fit the $q\Delta S_{exp}(q,\ t)^{\perp}$ for each time delay as a linear combination of the extracted $qA(q)^{\perp}$s. The validity of the $qA(q)^{\perp}$s and the kinetic model can be determined by assessing two criteria: 1) how well the linear combination of $qA(q)^{\perp}$s fits the $q\Delta S_{exp}(q,\ t)^{\perp}$s, and 2) how well the optimal weights of the $qA(q)^{\perp}$s obtained from the LCF align with the time-dependent concentration of each species calculated using our kinetic model. Upon analysis, we found that the linear combination of the $qA(q)^{\perp}$s shows excellent match to the experimental data, albeit not depicted here. Moreover, the weights of the $qA(q)^{\perp}$s derived from the LCF, represented by $C'(t)$ and displayed as empty circles in Figure 3, correspond well with the theoretically calculated time-dependent concentration from our kinetic model, indicated by $C(t)$ and presented as solid lines in Figure 3. These good agreements indicate that the experimental data can be well described using our kinetic model and its corresponding $qA(q)^{\perp}$s.

Structure refinement

Typically, the structure refinement using the TRXL data is achieved by fitting the experimental difference scattering curves to their theoretical counterparts.[6-7] The experimental difference scattering curves

contain not only solute-related terms but also contributions from the solvent's hydrodynamics. Consequently, the theoretical difference scattering curve is represented by the sum of three contributing terms: the solute-only term, the cage term, and the solvent term. Still, when the $q\mathrm{A}(q)$ is extracted from the experimental difference scattering curves and subsequently analyzed, the corresponding theoretical curve for $q\mathrm{A}(q)$ is calculated using a different approach from when analyzing the experimental difference scattering curves. Here, the term $q\mathrm{A}(q)$ is used to denote the SADS in q-space that is free from distortion caused by PEPC. Specifically, $q\mathrm{A}(q)$ is obtained by correcting $q\mathrm{A}(q)^{\perp}$ to compensate for the distortion in q-space caused by the PEPC-treatment, and thus represents a distortion-free SADS in q-space. The method for correcting the distortion of $q\mathrm{A}(q)^{\perp}$ is described in the "R-space analysis" section. The distinction arises between the analysis of the $q\Delta\mathrm{S}(q, t)$ and $q\mathrm{A}(q)$ because $q\mathrm{A}(q)$ solely includes contributions from solute-related terms, specifically the solute term and the cage term. In other words, theoretical $\mathrm{A}(q)$ ($\mathrm{A}_{\mathrm{theo}}(q)$) can be expressed by the following equation:

$$\mathrm{A}_{\mathrm{theo}}(q) = \Delta\mathrm{S}(q)_{\mathrm{solute}} + \Delta\mathrm{S}(q)_{\mathrm{cage}} \tag{S10}$$

, where $\Delta\mathrm{S}(q)_{\mathrm{solute}}$ represents the solute term, arising from the changes in the molecular structures of the solutes, and $\Delta\mathrm{S}(q)_{\mathrm{cage}}$ denotes the cage term, originating from the changes in the interatomic distances between the solute molecule and its surrounding solvent molecules. In Equation S10, $\Delta\mathrm{S}(q)_{\mathrm{solute}}$ and $\Delta\mathrm{S}(q)_{\mathrm{cage}}$ can be further elaborated and described in terms of the difference between the static terms, $\mathrm{S}(q)_{\mathrm{solute}}$ and $\mathrm{S}(q)_{\mathrm{cage}}$, for the products and reactants. For example, the theoretical curve for $\mathrm{A}_1(q)^{\perp}$ of the $[\mathrm{Ce(III)Cl_6}]^{3-}$/substrate sample, which corresponds to the formation of the excited state and does not involve the structural change of the substrate, as well as those for $\mathrm{A}_1(q)$ and $\mathrm{A}_2(q)$ of the $[\mathrm{Ce(III)Cl_6}]^{3-}$-only sample can be described by the following equation:

$$\mathrm{A}_{i,\mathrm{theo}}(q) = \mathrm{A}_{i,\mathrm{theo}}(q)_{\mathrm{catalyst}} = \frac{f_i}{R}\{(\mathrm{S}^{\mathrm{product}}(q)_{\mathrm{solute}} - \mathrm{S}^{\mathrm{reactant}}(q)_{\mathrm{solute}}) + (\mathrm{S}^{\mathrm{product}}(q)_{\mathrm{cage}} - \mathrm{S}^{\mathrm{reactant}}(q)_{\mathrm{cage}})\} \tag{S11}$$

, where $\mathrm{A}_{i,theo}(q)_{\mathrm{catalyst}}$ is the ith SADS in q-space corresponding to the structural change of the photocatalyst, f_i is the fractional concentration of the molecules in the products, R is the number ratio of the solvent molecules to the solute molecules, $\mathrm{S}^{\mathrm{X}}(q)_{\mathrm{solute}}$ represents the theoretical scattering curve of the photocatalyst in the X state (X = product or reactant), and $\mathrm{S}^{\mathrm{X}}(q)_{\mathrm{cage}}$ is the theoretical scattering curve of the solvent cage nearby the photocatalyst in the X state (X = product or reactant). For the theoretical curve for the $\mathrm{A}_2(q)^{\perp}$ of the $[\mathrm{Ce(III)Cl_6}]^{3-}$/substrate sample, which involves the structural change of the substrate, the equation for the theoretical SADS can be modified by adding the contribution from the substrate's structural change, as follows:

$$\begin{aligned}\mathrm{A}_{i,\mathrm{theo}}(q) = {}& \mathrm{A}_{i,\mathrm{theo}}(q)_{\mathrm{catalyst}} \\ & + \frac{f_i}{R}\{(\mathrm{S}^{\mathrm{FPh}}(q)_{\mathrm{solute}} + \mathrm{S}^{\mathrm{I}}(q)_{\mathrm{solute}} - \mathrm{S}^{\mathrm{FPhI}}(q)_{\mathrm{solute}}) + (\mathrm{S}^{\mathrm{FPh}}(q)_{\mathrm{cage}} + \mathrm{S}^{\mathrm{I}}(q)_{\mathrm{cage}} - \mathrm{S}^{\mathrm{FPhI}}(q)_{\mathrm{cage}})\}\end{aligned} \tag{S12}$$

, where $\mathrm{S}^{\mathrm{X}}(q)_{\mathrm{solute}}$ represents the theoretical scattering curve of X and $\mathrm{S}^{\mathrm{X}}(q)_{\mathrm{cage}}$ is the theoretical scattering curve of the solvent cage nearby X. FPh, I, and FPhI in the superscript denote fluorobenzene, iodide ion, and 1-fluoro-4-iodobenzene, respectively.

For the analysis of $\mathrm{A}_i(q)$s, we select model structures corresponding to each species, including that for $[\mathrm{Ce(III)Cl_6}]^{3-}$(GS), and optimize the structural parameters of the model structures. The optimization process involves varying the parameters and calculating the $\mathrm{A}_{i,\mathrm{theo}}(q)$s corresponding to the modified parameters. This

iterative process aims to find the optimal parameters that minimize the discrepancy between the experimental SADSs and theoretical SADSs. Hereafter, we will exclusively use the notation $A_{i,\text{exp}}(q)$ to refer to the experimental SADS, ensuring clear distinction from the theoretical SADS, $A_{i,\text{theo}}(q)$. To quantitatively measure the discrepancy between the experimental and theoretical SADSs, we employ the χ_i^2 criteria for each species as follows:

$$\chi_i^2 = \sum_q \left(\frac{A_{i,\text{exp}}(q) - A_{i,\text{theo}}(q)}{\sigma_i(q)}\right)^2 \tag{S13}$$

, where $\sigma_i(q)$ is the standard error of the mean of the SADS of the ith species.

In this work, we considered two additional factors when calculating the theoretical SADS, in addition to the standard Debye equation for the solute term and the cage term calculation aided by molecular dynamics simulations. Firstly, we addressed the distortion of the shape of SADS in q-space caused by the PEPC treatment. Although the PEPC treatment facilitates the kinetic analysis by eliminating the contribution of solvent term kinetics from the experimental data, it is known to alter the shape of the SADS in q-space, necessitating a modified approach. To account for this distortion, we applied the PEPC treatment to the $A_{i,\text{theo}}(q)$ calculated by using Equations S11 or S12, introducing the distortion in the same manner. Subsequently, we performed the comparison between the PEPC-treated theoretical SADS of the ith species, denoted as $A_{i,\text{theo}}(q)^{\perp}$, and the PEPC-treated experimental SADS of the ith species, $A_{i,\text{exp}}(q)^{\perp}$. Secondly, we observed a baseline artifact in the $A_{i,\text{exp}}(q)^{\perp}$s that could not be accounted for by the sum of the Debye curve and the cage term. We considered the contribution of this baseline artifact in the $A_{i,\text{theo}}(q)^{\perp}$s as well. This artifact appears linear on $qA_{i,\text{exp}}(q)^{\perp}$, $A_{i,\text{exp}}(q)^{\perp}$ multiplied by q, as shown in Figure S11. Assuming that the baseline on the $qA_{i,\text{exp}}(q)^{\perp}$s can be expressed in the form of $a_iq + b_i$, we applied a correction of $a_i + b_i/q$ to the $A_{i,\text{theo}}(q)^{\perp}$s. Taking into account these additional factors, the corrected theoretical SADS, $A_{i,\text{theo}}(q)^{\perp\prime}$, which includes the linear baseline correction and the shape distortion in q-space due to the PEPC treatment, can be expressed as follows:

$$A_{i,\text{theo}}(q)^{\perp}{}' = A_{i,\text{theo}}(q)^{\perp} + \Delta S_{i,\text{baseline}}(q) = A_{i,\text{theo}}(q)^{\perp} + a_i + \frac{b_i}{q} \tag{S14}$$

, where $A_{i,\text{theo}}(q)^{\perp}$ is the PEPC-treated theoretical SADS, prepared by applying the PEPC treatment on the theoretical SADS generated using the standard approach for calculating the solute term and cage term, and $\Delta S_{i,\text{baseline}}(q)$ is the baseline artifact for the ith experimental SADS.

For the structural analysis of $A_{i,\text{exp}}(q)^{\perp}$s, we performed a global fitting of both $A_{1,\text{exp}}(q)^{\perp}$ and $A_{2,\text{exp}}(q)^{\perp}$ instead of fitting them individually using Equation S13. This global fitting approach was adopted to share a common, optimized ground-state structure of the photocatalyst during the structural analysis of the two $A_{i,\text{exp}}(q)^{\perp}$s. The global fitting process can be expressed mathematically as minimizing the χ^2 of the following equation.

$$\chi^2 = \sum_i \sum_q \left(\frac{A_{i,\text{exp}}(q)^{\perp} - A_{i,\text{theo}}(q)^{\perp}{}'}{\sigma_i(q)}\right)^2 \tag{S15}$$

We note that $A_{i,\text{exp}}(q)^{\perp}$ can be extracted from the PEPC-treated experimental data, $\Delta S(q, t)^{\perp}$, and is equivalent to $A_i(q)^{\perp}$ in Equations S5 and S9.

For the structure refinement, we employed the DFT-optimized structures of $[Ce(III)Cl_6]^{3-}$(GS), $[Ce(III)Cl_6]^{3-}$(ES), and $[Ce(IV)Cl_6]^{2-}$ as the starting model structures. Since $[Ce(III)Cl_6]^{3-}$ has an octahedral symmetry, the structure can be described by one Ce–Cl bond length. However, to consider cases where octahedral

symmetry breaks down, we performed the structure refinement by selecting the two bond lengths as fitting parameters: the bond length between the equatorial Cl ligand and Ce and that between the axial Cl ligand and Ce. $A_{i,\text{theo}}(q)^{\perp}$ was calculated by considering not only the structural changes of the photocatalyst but also the structural changes of the substrate. During the photoreaction, 1-fluoro-4-iodobenzene reacts with the photocatalyst to form fluorobenzene radical and iodide ion. In addition, the fluorobenzene radical reacts with a solvent molecule to form fluorobenzene and the hydrogen-abstracted solvent molecule. Since the concentration of the solvent molecule is high, the reaction between the fluorobenzene radical and solvent molecule is expected to occur rapidly after the generation of the fluorobenzene radical. Considering these points, we included the contribution of the reaction where 1-fluoro-4-iodobenzene is converted to fluorobenzene and iodide ion in the calculation of $A_{i,\text{theo}}(q)$, as shown in Equation S12. The effect of the hydrogen abstraction of a solvent molecule on the scattering curve was neglected when calculating $A_{i,\text{theo}}(q)$ because the difference scattering signal originating from the abstraction of the hydrogen atom is significantly small.

As mentioned earlier, the two $A_{i,\text{exp}}(q)^{\perp}$s obtained from KCA of one dataset were globally fitted. As can be seen in Equation S11, for both cases that involve the formation $[Ce(III)Cl_6]^{3-}$(ES) or $[Ce(IV)Cl_6]^{2-}$, the theoretical scattering curve of $[Ce(III)Cl_6]^{3-}$(GS) is required for the calculation of $A_{i,\text{theo}}(q)_{\text{catalyst}}$. In other words, the structure of $[Ce(III)Cl_6]^{3-}$(GS) is required for the calculation of both $A_{1,\text{theo}}(q)_{\text{catalyst}}$ and $A_{2,\text{theo}}(q)_{\text{catalyst}}$. Taking this into consideration, we used the same structure of $[Ce(III)Cl_6]^{3-}$(GS) to generate both $A_{1,\text{theo}}(q)_{\text{catalyst}}$ and $A_{2,\text{theo}}(q)_{\text{catalyst}}$. By using $A_{i,\text{theo}}(q)_{\text{catalyst}}$s, we calculated $A_{1,\text{theo}}(q)$ and $A_{2,\text{theo}}(q)$ via Equations S11 or S12. We applied PEPC to the $A_{i,\text{theo}}(q)$s to yield $A_{i,\text{theo}}(q)^{\perp}$s and then corrected for the baseline. The resulting $A_{1,\text{theo}}(q)^{\perp\prime}$ and $A_{2,\text{theo}}(q)^{\perp\prime}$ were globally fitted to $A_{1,\text{exp}}(q)^{\perp}$ and $A_{2,\text{exp}}(q)^{\perp}$ by minimizing χ^2 in Equation S15. Through this process, we found the structure of $[Ce(III)Cl_6]^{3-}$(GS) that can explain both $A_{1,\text{exp}}(q)^{\perp}$ and $A_{2,\text{exp}}(q)^{\perp}$.

We estimated the error for each structural parameter by locating the value (p_1) at which the χ^2 value is one unit larger than the optimized parameter (p_o) and calculating $|p_1\text{-}p_o|$. The χ^2 value was derived using the formula in Equation S15. Specifically, we indirectly calculated the $|p_1\text{-}p_o|$ by employing the HESSE function of MINUIT (FMINUIT) instead of directly locating p_1 to estimate the error.[8] In the HESSE function, the hessian matrix (H) of χ^2 is calculated and the errors are estimated from the H by calculating the diagonal elements of $(2 \times H^{-1})$. It should be noted that the errors accounted for here only represent random errors, not systematic errors. The seemingly small errors merely reflect the precision of the measurements, rather than their accuracy.

Due to the symmetric structure of $[Ce(III)Cl_6]^{3-}$, the variation of Ce–Cl distance does not have a significant impact on the shape of the difference scattering curve. As a result, a correlation arises between the photoexcited population and the magnitude of Ce–Cl distance change. In order to address these issues, the structure refinement was initially performed for the data of the $[Ce(III)Cl_6]^{3-}$/substrate sample. For this sample, where the substrate is also involved in the reaction alongside the photocatalyst, the correlation between the photoexcited population and the change in Ce–Cl distance decreases in TRXL data. Thus, by conducting the structure refinement on the $[Ce(III)Cl_6]^{3-}$/substrate data, we obtained information regarding the structures of $[Ce(III)Cl_6]^{3-}$(GS), $[Ce(III)Cl_6]^{3-}$(ES), and $[Ce(IV)Cl_6]^{2-}$. In the refinement of the structure for the $[Ce(III)Cl_6]^{3-}$-only sample, the structures obtained from the analysis of the $[Ce(III)Cl_6]^{3-}$/substrate sample were fixed, which allowed obtaining information related to f_i in Equation S11, such as the population of the excited state.

In addition to the structure refinement using PEPC-treated data, we performed global fitting analysis (GFA) on the experimental data without the PEPC treatment for comparison. The structural parameters obtained

from two methods exhibited consistency, thereby supporting the validity of the structure refinement method using PEPC-treated data. Further details can be found in the "Comparison of the results of structural analysis using PEPC and global fitting analysis" section.

Estimation of systematic errors in the structure refinement

The accuracy of the results of structural refinement can be compromised due to the presence of systematic errors. Notably, utilizing inaccurate parameters in data processing and analysis can affect the difference scattering curves as well as the results of structural analysis. Particular attention must be given to the accuracy of experimental parameters, including the sample-to-detector distance, X-ray energy, and orientation of the detector, specifically the angle between the X-ray propagation direction and the normal vector of the detector plane. To minimize the potential impact of such systematic errors, it is common to measure experimental parameters before commencing TRXL experiments. For instance, the distribution of X-ray energy can be precisely determined using a monochromator, and the sample-to-detector distance and detector geometry can be calibrated by collecting and analyzing the diffraction pattern from a reference powder sample. These meticulous measures are typically implemented to restrict errors associated with experimental parameters, thereby ensuring the integrity of acquired data and the accuracy of subsequent structural analyses. For example, the ID09 beamline of ESRF has an error of approximately 0.1% for the X-ray energy and less than 0.1° for the angle between the detector plane and the X-ray beam direction. While establishing a precise error boundary for the sample-to-detector distance remains challenging, it is reasonable to assume that the error likely falls within a range of hundreds of micrometers, which corresponds to 1% of a typical sample-to-detector distance.

To briefly assess the potential impact of the errors in determining the experimental parameters on the result of structural refinement, we conducted a series of simulations. In these simulations, we intentionally introduced distortions into the experimental SADSs to simulate scenarios where we erroneously determined the experimental parameters, specifically the sample-to-detector distance and X-ray energy. For the simulations, we assumed that the detector distance and the X-ray energy could be erroneously determined by up to 1 mm and 1%, respectively, which are beyond the typical error ranges of the parameters. We then used the resulting distorted SADSs for structure refinement and compared the derived structural parameters with those obtained from the pristine "reference" SADS counterparts. The resulting structural parameters are compared in Table S4.

As shown in the comparison provided in Table S4, the errors in the sample-to-detector distance and X-ray energy have a relatively modest impact on the results of the structure refinement. The systematic errors introduced by these errors in the experimental parameters are found to be less than 2%. It is worth noting that the symmetry of the resulting structures, specifically, whether the equatorial and axial bond distances are equal or not, does not depend on the experimental parameters. Moreover, it consistently emerged that, regardless of the systematic errors, the structural parameters optimized using the PBE0 functionals in DFT calculations exhibited the highest level of overall consistency with the parameters obtained from the structural refinement (Table 1).

In addition to errors in the sample-to-detector distance and X-ray energy, it is worth considering the potential impact of errors in detector geometry on the result of structural refinement. In the case of detector geometry, the measurement using a reference powder is effective in minimizing errors in the angle between the normal vector of the detector plane and the propagation direction of the X-ray beam, typically reducing it to less than approximately 0.1°. The tilt of the detector would produce an effect similar to spreading the sample-to-

detector distance within a range determined by the tilt angle and size of the detector. Compared to a situation where the sample-to-detector distance changes by a specific value (for example, 150 μm), spreading the sample-to-detector distance within the same value (from 0 to 150 μm) would lead to significantly less distortion in the various scattering curves. Considering the detector used in the experiment (Rayonix MX170-HS), the tilt of the detector by 0.1° would result in spreading in the detector distance within the range of -150 μm to 150 μm. Therefore, it is expected that such minor errors in detector geometry are likely to result in a relatively negligible systematic error when compared to the error associated with the 0.5 mm variation in the sample-to-detector distance. As a result, our estimation indicates that errors in detector geometry may introduce structural parameter errors less than 1%.

Effect of polychromatic correction on the result of structural refinement

In this work, we applied a "pink beam", which is a polychromatic beam that has not been subjected to monochromators or multilayer optics, as a probe. Because of its polychromatic nature, the pink beam comprises photons with diverse wavelengths. As a result, the scattering pattern produced by this beam is broader in comparison to that of a monochromatic beam. Thus, when analyzing the scattering patterns generated by a pink beam, it is typically essential to account for this broadening effect by applying a "polychromatic correction" to the theoretical curve, which is then compared with the experimental data broadened by the polychromatic beam.[9]

However, in our specific case, it was observed that the polychromatic beam-induced broadening effect had a relatively minor impact on our experimental data. This is primarily attributed to the relatively narrow bandwidth of the pink beam we employed. Since the upgrade of ESRF (ESRF-Extremely Brilliant Source, ESRF-EBS) in 2019, the X-ray energy spectrum has become significantly narrower, and even the pink beam has a substantially reduced low-energy tail in the energy spectrum. The energy spectra of the pink beam of ESRF after (ESRF-EBS, red line) and before (ESRF-OLD, blue line) the upgrade are depicted in Figure S12A (red and blue lines). Consequently, the effect of polychromatic correction was negligible in our case. To demonstrate this point, we generated a theoretical difference scattering curve for the formation of $[Ce(III)Cl_6]^{3-}$(ES), assuming perfectly monochromatic X-ray beam (Figure S12B, black line). For comparison, we generated other difference scattering curves for the same species under the pink beam conditions of ESRF-EBS (Figure S12B, red line) and ESRF-OLD (Figure S12B, blue line). These latter curves were obtained by applying a polychromatic correction to the difference scattering curve for the monochromatic beam. Comparison of these curves in Figure S12B reveals that the disparity between the difference scattering curves for the monochromatic and polychromatic beams of ESRF-EBS is negligible. Conversely, a pronounced contrast emerges when comparing the difference scattering curves between the monochromatic and polychromatic beams of ESRF-OLD, revealing a substantial disparity.

To provide more direct and quantitative support, we conducted structural refinement with the application of the polychromatic correction and compared the results with those obtained without applying the polychromatic correction. While the reaction yield for the region interrogated by the X-ray pulse was marginally changed from 0.105 to 0.111, the application of the polychromatic correction had minimal impact on the derived structural parameters as shown in Table S5. Specifically, differences of less than 0.3% are observed for the structural parameters. Consequently, we conclude that the polychromatic correction is not necessary for our structural refinement.

Comparison of the results of structural analysis using PEPC and global fitting analysis

We extracted detailed structural parameters by analyzing the $q\mathrm{A}(q)^{\perp}$s, the SADSs extracted from the PEPC-treated experimental data, $q\Delta\mathrm{S}_{\mathrm{exp}}(q, t)^{\perp}$s. Nevertheless, we acknowledge that due to the distortion of the shape of the experimental data in q-space, introduced by the PEPC treatment, some might question the precision or accuracy of our structural analysis based on the PEPC-treated data. Bearing this in mind, we also employed the widely-used GFA technique to analyze the same data (without the PEPC treatment) and compared the resultant structural parameters with those obtained from the analysis of PEPC-treated data.

In this GFA analysis, we focused on optimizing the molecular structural parameters. Kinetic parameters, such as rate constants, were fixed during the fitting process. In addition to the structural parameters, $\Delta\mathrm{T}(t)$ and $\Delta\rho(t)$ at individual time delays were also optimized as fitting parameters. The resulting structural parameters, together with those from the structural analysis of $q\mathrm{A}(q)^{\perp}$s, are listed in Table S6. Upon comparison of the structural parameters obtained from this GFA with those derived from the analysis of $q\mathrm{A}(q)^{\perp}$s, we observe a remarkable consistency between the two sets of parameters. It is noteworthy that these sets almost perfectly align, supporting the reliability of our structural analysis employing the PEPC-treated curves.

Sensitivity of TRXL data to the different types of structural changes in $[\mathrm{Ce(III)Cl_6}]^{3-}$

The TRXL data exhibits remarkable sensitivity to structural changes in $[\mathrm{Ce(III)Cl_6}]^{3-}$, enabling the discrimination of various types of structural changes, including symmetric or asymmetric contraction of Ce–Cl bonds. To demonstrate this sensitivity, we conducted simulations focusing on three distinct types of structural changes: 1) symmetric contraction of Ce–Cl bonds, 2) asymmetric contraction of Ce–Cl bonds with a larger contraction in axial Ce–Cl bonds, and 3) asymmetric contraction of Ce–Cl bonds with a larger contraction in equatorial Ce–Cl bonds. We calculated theoretical difference scattering curves corresponding to each of the three different types of structural changes. Specifically, in the first case, both axial and equatorial Ce–Cl bond lengths were symmetrically decreased by 0.1 Å. In the second case, the axial Ce–Cl bond lengths were decreased by 0.15 Å, while the equatorial Ce–Cl bond lengths were decreased by 0.05 Å. In the third case, the axial Ce–Cl bond lengths were decreased by 0.05 Å, and the equatorial Ce–Cl bond lengths were decreased by 0.15 Å. The corresponding theoretical difference curves are shown in Figure S13. As shown in Figure S13, each curve exhibits unique amplitudes and shapes, enabling the discrimination of distinct structural changes by analyzing the curve. The simulation results demonstrate that, with a sufficient SNR in the TRXL data, it is possible to retrieve detailed structural changes beyond the mere contraction of Ce–Cl bond lengths. For example, it becomes feasible to distinguish between the maintaining or breaking of the octahedral symmetry with the Ce–Cl bond contraction.

Calculation of theoretical difference scattering curves

The theoretical difference scattering curves ($q\Delta\mathrm{S}_{\mathrm{theo}}(q, t)$), which correspond to the experimental data ($q\Delta\mathrm{S}_{\mathrm{exp}}(q, t)$) and are shown in Figures 2A and 2B, were calculated based on the kinetic framework and the structure of each species determined from the kinetic analysis and structure refinement. Another crucial factor considered to generate $q\Delta\mathrm{S}_{\mathrm{theo}}(q, t)$ is the contribution of the solvent term and the experimental artifact, which is not accounted for in our kinetic analysis thanks to the implementation of the PEPC treatment. By employing PEPC, the contributions of solvent heating and artifacts arising from high laser power were eliminated from the data. Mathematically, it can be expressed as follows.

$$\Delta S_{\exp}(q,t)^{\perp} = \Delta S_{\exp}(q,t) - d_{\mathrm{T}}(t)(\frac{\partial S}{\partial \mathrm{T}})_{\rho} - d_{\rho}(t)(\frac{\partial S}{\partial \rho})_{\mathrm{T}} - d_{\tau}(t)\tau(q) \qquad \text{(S16)}$$

, where $(\partial S/\partial T)_{\rho}$ and $(\partial S/\partial \rho)_T$ represent the solvent scattering change per unit temperature and density change, respectively. The term $\tau(q)$ refers to the artifact resulting from high laser power, which we named by taking the first letter from τεχνούργημα, the Greek word for "artifact". The coefficients $d_T(t)$, $d_{\rho}(t)$, and $d_{\tau}(t)$ correspond to the contributions of $(\partial S/\partial T)_{\rho}$, $(\partial S/\partial \rho)_T$, and $\tau(q)$ at the time delay t. Using Equations S5 and S16, $\Delta S_{exp}(q, t)$ can be expressed as follows.

$$\begin{aligned}\Delta S_{\exp}(q,t) &= \Delta S_{\exp}(q,t)^{\perp} + d_{\mathrm{T}}(t)(\frac{\partial S}{\partial \mathrm{T}})_{\rho} + d_{\rho}(t)(\frac{\partial S}{\partial \rho})_{\mathrm{T}} + d_{\tau}(t)\tau(q) \\ &= \sum_i \mathrm{A}_{i,\exp}(q)^{\perp} \cdot c_i(t) + d_{\mathrm{T}}(t)(\frac{\partial S}{\partial \mathrm{T}})_{\rho} + d_{\rho}(t)(\frac{\partial S}{\partial \rho})_{\mathrm{T}} + d_{\tau}(t)\tau(q)\end{aligned} \qquad \text{(S17)}$$

The mathematical relation between $\Delta S_{theo}(q, t)$ and $A_{i,theo}(q)^{\perp}$'s is analogous to the relation between $\Delta S_{exp}(q, t)$ and $A_{i,exp}(q)^{\perp}$s shown in Equation S17. Based on the relation, $\Delta S_{theo}(q, t)$ can be obtained from $A_{i,theo}(q)^{\perp\prime}$s, which are determined from the structure refinement. Specifically, $\Delta S(q, t)_{theo}$ can be expressed as follows.

$$\Delta S_{\mathrm{theo}}(q,t) = \sum_i \mathrm{A}_{i,\mathrm{theo}}(q)^{\perp\prime} \cdot c_i(t) + d_{\mathrm{T}}(t)(\frac{\partial S}{\partial \mathrm{T}})_{\rho} + d_{\rho}(t)(\frac{\partial S}{\partial \rho})_{\mathrm{T}} + d_{\tau}(t)\tau(q) \qquad \text{(S18)}$$

***R*-space analysis**

As reported in our publication on the PEPC method, the SADS obtained from the PEPC-treated data, denoted as $A_{i,exp}(q)^{\perp}$, is distorted in q-space.[1] However, the distortion-free $A_{i,exp}(q)$ can be obtained through a successful structure refinement process applied to $A_{i,exp}(q)^{\perp}$. The following describes the process and underlying principle in detail. In this study, the kinetic contributions of three components ($(\partial S/\partial T)_{\rho}$, $(\partial S/\partial \rho)_T$, and an artifact arising from the high laser power) were removed through the PEPC process, as mentioned in the "Kinetic analysis" section. Under this condition, the mathematical relationship between $A_{i,exp}(q)$ and $A_{i,exp}(q)^{\perp}$ can be expressed as follows.

$$\mathrm{A}_{i,\exp}(q)^{\perp} = \mathrm{A}_{i,\exp}(q) - d_{i,\mathrm{T}}(\frac{\partial S}{\partial \mathrm{T}})_{\rho} - d_{i,\rho}(\frac{\partial S}{\partial \rho})_{\mathrm{T}} - d_{i,\tau}\tau(q) \qquad \text{(S19)}$$

, where the coefficients $d_{i,T}$, $d_{i,\rho}$, and $d_{i,\tau}$ correspond to the contributions of $(\partial S/\partial T)_{\rho}$, $(\partial S/\partial \rho)_T$, and $\tau(q)$ in $A_{i,exp}(q)$ which were excessively removed through the PEPC process and caused distortion in q-space in the $A_{i,exp}(q)^{\perp}$. A successful structure refinement minimizing χ^2 in Equation S15 yields $A_{i,theo}(q)^{\perp\prime}$ that is close to $A_{i,exp}(q)^{\perp}$. The relation between the $A_{i,theo}(q)^{\perp\prime}$ and $A_{i,exp}(q)^{\perp}$ resulting from the successful structure refinement can be expressed as follows.

$$\begin{aligned}\mathrm{A}_{i,\exp}(q)^{\perp} \simeq \mathrm{A}_{i,\mathrm{theo}}(q)^{\perp\prime} &= \mathrm{A}_{i,\mathrm{theo}}(q)^{\perp} + a_i + \frac{b_i}{q} \\ &= \mathrm{A}_{i,\mathrm{theo}}(q) - \gamma_{i,\mathrm{T}}(\frac{\partial S}{\partial \mathrm{T}})_{\rho} - \gamma_{i,\rho}(\frac{\partial S}{\partial \rho})_{\mathrm{T}} - \gamma_{i,\tau}\tau(q) + a_i + \frac{b_i}{q}\end{aligned} \qquad \text{(S20)}$$

, where $\gamma_{i,T}$, $\gamma_{i,\rho}$, and $\gamma_{i,\tau}$ are the contributions of $(\partial S/\partial T)_{\rho}$, $(\partial S/\partial \rho)_T$, and $\tau(q)$ in $A_{i,theo}(q)$, which were excessively removed through the PEPC process. It is worth noting that after the successful structure refinement, the optimal

structures of reactants and products can be determined along with the optimal constants for the baseline, a_i and b_i. Once the optimal structures are obtained, $\mathrm{A}_{i,\mathrm{theo}}(q)$ can be calculated straightforwardly from them. Then, $\gamma_{i,\mathrm{T}}$, $\gamma_{i,\rho}$, and $\gamma_{i,\tau}$ can be determined from the $\mathrm{A}_{i,\mathrm{theo}}(q)$. The structure refinement is based on the following assumption: if $\mathrm{A}_{i,\mathrm{exp}}(q)^{\perp} \simeq \mathrm{A}_{i,\mathrm{theo}}(q)^{\perp\prime}$, then $\mathrm{A}_{i,\mathrm{exp}}(q) \simeq \mathrm{A}_{i,\mathrm{theo}}(q)$. Based on this assumption, Equation S20 can be expressed as follows:

$$\begin{aligned}\mathrm{A}_{i,\mathrm{exp}}(q)^{\perp} &\simeq \mathrm{A}_{i,\mathrm{theo}}(q) - \gamma_{i,\mathrm{T}}\left(\frac{\partial S}{\partial \mathrm{T}}\right)_{\rho} - \gamma_{i,\rho}\left(\frac{\partial S}{\partial \rho}\right)_{\mathrm{T}} - \gamma_{i,\tau}\tau(q) + a_i + \frac{b_i}{q} \\ &\simeq \mathrm{A}_{i,\mathrm{exp}}(q) - \gamma_{i,\mathrm{T}}\left(\frac{\partial S}{\partial \mathrm{T}}\right)_{\rho} - \gamma_{i,\rho}\left(\frac{\partial S}{\partial \rho}\right)_{\mathrm{T}} - \gamma_{i,\tau}\tau(q) + a_i + \frac{b_i}{q}\end{aligned} \quad \text{(S21)}$$

Using Equation S21, $\mathrm{A}_{i,\mathrm{exp}}(q)$ can be obtained as follows.

$$\mathrm{A}_{i,\mathrm{exp}}(q) \simeq \mathrm{A}_{i,\mathrm{exp}}(q)^{\perp} + \gamma_{i,\mathrm{T}}\left(\frac{\partial S}{\partial \mathrm{T}}\right)_{\rho} + \gamma_{i,\rho}\left(\frac{\partial S}{\partial \rho}\right)_{\mathrm{T}} + \gamma_{i,\tau}\tau(q) - a_i - \frac{b_i}{q} \quad \text{(S22)}$$

The resulting $\mathrm{A}_{i,\mathrm{exp}}(q)$s are displayed in Figures 4A and 4B.

Once $\mathrm{A}_{i,\mathrm{exp}}(q)$s are determined, their corresponding $\Pi_i(r)$s, which can be considered as the species-associated difference radial distribution functions or the SADSs in r-space, can be calculated via Fourier sine transform. The mathematical relation between $\mathrm{A}_{i,\mathrm{exp}}(q)$ and $\Pi_{i,\mathrm{exp}}(r)$ can be expressed as follows:

$$r^2\Pi_{i,\mathrm{exp}}(r) = \frac{r}{2\pi^2}\int q\mathrm{A}_{i,\mathrm{exp}}(q)\sin(qr)\exp(-q^2\alpha)dq \quad \text{(S23)}$$

, where α denotes a damping constant to account for the finite q range covered by the experiment. We used α = 0.03 Å^2 in our analysis. The resulting $r^2\Pi_{i,\mathrm{exp}}(r)$s, $r^2\Pi_{1,\mathrm{exp}}(r)$ and $r^2\Pi_{2,\mathrm{exp}}(r)$, are depicted in Figures 4C and 4D. Meanwhile, we attempted Fourier sine transform on the uncorrected $\mathrm{A}_{i,\mathrm{exp}}(q)^{\perp}$ as a test. As a result, we obtained $\Pi_{i,\mathrm{exp}}(r)^{\perp}$ as shown in the following equation:

$$r^2\Pi_{i,\mathrm{exp}}(r)^{\perp} = \frac{r}{2\pi^2}\int q\mathrm{A}_{i,\mathrm{exp}}(q)^{\perp}\sin(qr)\exp(-q^2\alpha)dq \quad \text{(S24)}$$

The resulting $r^2\Pi_{i,\mathrm{exp}}(r)^{\perp}$s, are depicted in Figures S5D and S5E.

Upon comparison of $r^2\Pi_{i,\mathrm{exp}}(r)$ and $r^2\Pi_{i,\mathrm{exp}}(r)^{\perp}$, we observed that their general trends, such as the approximate locations of positive and negative peaks, were remarkably similar. However, significant differences in the detailed features, such as the relative peak heights, were also observed. This discrepancy was attributed to the distortion present in $q\mathrm{A}_1(q)^{\perp}$ and $q\mathrm{A}_2(q)^{\perp}$ due to the PEPC treatment, which led to less accurate $r^2\Pi_{1,\mathrm{exp}}(r)^{\perp}$ and $r^2\Pi_{2,\mathrm{exp}}(r)^{\perp}$ compared to the results presented in Figure 4. To address this issue, we corrected the distortion in $q\mathrm{A}_1(q)^{\perp}$ and $q\mathrm{A}_2(q)^{\perp}$ to obtain the distortion-free $q\mathrm{A}_1(q)$ and $q\mathrm{A}_2(q)$, and subsequently used them to obtain $r^2\Pi_{1,\mathrm{exp}}(r)$ and $r^2\Pi_{2,\mathrm{exp}}(r)$ by performing Fourier sine transform.

In order to aid the interpretation of the obtained $r^2\Pi_{i,\mathrm{exp}}(r)$s, we plotted bar charts in Figures 4C, 4D, S5D, and S5E which illustrate the interatomic pair distances that disappear and emerge in the products. The interatomic pair distances that originally exist in the reactants (for example, $[\mathrm{Ce(III)Cl_6}]^{3-}$(GS)) but disappear in the products (for example, the excited and oxidized states of the photocatalyst) contribute as negative peaks in the corresponding $r^2\Pi_{i,\mathrm{exp}}(r)$, while those that emerge in the products contribute as positive peaks. By comparing the positions of these negative (downward) and positive (upward) bars with the locations of the negative and positive peaks in the $r^2\Pi_{i,\mathrm{exp}}(r)$, we can identify which changes in the pair distances contribute to the difference radial

distribution functions ($r^2\Delta R(r)$s). In the products, the Ce–Cl distance is shorter than in $[Ce(III)Cl_6]^{3-}$(GS), resulting in a negative red bar at a longer Ce–Cl distance and a positive red bar at a shorter distance (Figures 4C and S5D). This contraction also affects the Cl–Cl distances, indicated by the black (adjacent Cl···Cl, *cis*) and blue (opposite Cl···Cl, *trans*) positive and negative bars.

By comparing the positions of the positive and negative peaks in the $r^2\Pi_{i,\mathrm{exp}}(r)$s with the highest amplitudes (at $r = 2 - 3$ Å) to the locations of the bars in the bar chart, it can be assigned that the dominant factor contributing to the overall structural change is indeed the Ce–Cl distance contraction (indicated by red positive and negative bars). In addition to this, we can see that the contraction of *cis* Cl···Cl distances (at $r \sim 4$ Å) also contributes significantly. The degree of contribution from *cis* Cl···Cl is considerably larger than those of the *trans* Cl···Cl (at $r = 5 - 6$ Å). This is because out of the total 15 Cl···Cl pair distances, only three correspond to *trans*, while the remaining 12 Cl···Cl distances correspond to *cis*. Accordingly, *cis* Cl···Cl contributes four times more significantly than *trans* Cl···Cl.

Upon comparison of $r^2\Pi_{1,\mathrm{exp}}(r)$ for the $[Ce(III)Cl_6]^{3-}$-only and $[Ce(III)Cl_6]^{3-}$/substrate samples, we observe a close resemblance in their shape, as shown in Figure 4C (red and black curves). This confirms that the presence of the substrate has a minimal effect on the structural change corresponding to the 1st species, $[Ce(III)Cl_6]^{3-}$(ES). However, the same comparison for $r^2\Pi_{2,\mathrm{exp}}(r)$ reveals a significant difference between the red and black curves in Figure 4D, which is clearly due to the presence or absence of the substrate. To qualitatively analyze this difference, we plotted the corresponding bar graphs depicting the substrate's structural changes. We note that the contribution of the structural change in the photocatalyst to $r^2\Pi_{1,\mathrm{exp}}(r)$ can be intuitively analyzed due to the dominant contributions of the three pair distances, the Ce–Cl, (*trans*) Cl···Cl, and (*cis*) Cl···Cl distances. Accordingly, the $r^2\Pi_{1,\mathrm{exp}}(r)$ can be analyzed by simply comparing the positions of the distances of the three pairs with the locations of the peaks or valleys in the $r^2\Pi_{1,\mathrm{exp}}(r)$. However, in the case of $r^2\Pi_{2,\mathrm{exp}}(r)$, the structural change in the substrate as well as that in the photocatalyst contributes to $r^2\Pi_{2,\mathrm{exp}}(r)$. For the substrate, numerous pair distances including those from the cage structure significantly contribute to $r^2\Delta R(r)$ without any one of them being particularly dominant. The substantial contributions of multiple pair types in $r^2\Delta R(r)$, combined with their peak and valley positions overlapping and offsetting each other, make the analysis of $r^2\Delta R(r)$ for the substrate (the difference between $r^2\Pi_2(r)$s for the $[Ce(III)Cl_6]^{3-}$/substrate and $[Ce(III)Cl_6]^{3-}$-only samples, blue curve in Figure 4D) more complex compared to that corresponding to the photocatalyst. In short, it is challenging to ascertain the contributions of each individual pair by solely comparing the atomic pair distances in the bar chart to the peaks and valleys in $r^2\Delta R(r)$ for the substrate. Nonetheless, despite this complexity, the contributions of certain pairs that had distinct manifestations in $r^2\Delta R(r)$ for the substrate can still be intuitively discerned. These pairs were characterized by their unique positions, which did not overlap with those of other pairs, allowing for a more straightforward interpretation of their contributions in $r^2\Delta R(r)$ for the substrate. A prominent example is the contribution of the F···I atomic pair. The dissociation of the C–I bond in the substrate causes the F···I atomic pair to disappear, which is reflected by the negative magenta bar in Figures 4D and S5E. In addition, we observed that the changes in specific atomic pair distances associated with the cage structure are clearly evident in the $r^2\Delta R(r)$ for the substrate. To investigate the change in cage structure, we conducted molecular dynamics simulations as described in the "Molecular Dynamics (MD) Simulations" section of the Supporting Information (SI). Our simulations reveal that the cage of the newly created iodide ion (I^-) produces a characteristic pair distance of

approximately 4 Å between I^- and the solvent's methyl group. The pair distance is represented by the positive magenta bar in Figures 4D and S5E. The positions of the positive and negative magenta bars determined in this way match well with the peak and valley positions of the residual curve (Figure 4D, blue) between the two $r^2\Pi_{2,\mathrm{exp}}(r)$s.

To further confirm that the difference between the two $r^2\Pi_{2,\mathrm{exp}}(r)$s is due to the substrate's reaction, we calculated the theoretical $r^2\Delta R(r)$ corresponding to the substrate's structural change. This was calculated as follows. First, we calculated the theoretical difference scattering curve corresponding to the reaction of the substrate as follows.

$$\Delta S_{\mathrm{substrate,theo}}(q) = \frac{f_i}{R}\{(S^{\mathrm{FPh}}(q)_{\mathrm{solute}} + S^{\mathrm{I}}(q)_{\mathrm{solute}} - S^{\mathrm{FPhI}}(q)_{\mathrm{solute}}) + (S^{\mathrm{FPh}}(q)_{\mathrm{cage}} + S^{\mathrm{I}}(q)_{\mathrm{cage}} - S^{\mathrm{FPhI}}(q)_{\mathrm{cage}})\} \tag{S25}$$

By Fourier sine transforming the resulting $\Delta S_{\mathrm{substrate,theo}}(q)$, we obtained $r^2\Delta R_{\mathrm{substrate,theo}}(r)$ corresponding to the reaction of the substrate. The resulting $r^2\Delta R_{\mathrm{substrate,theo}}(r)$ is depicted in Figure 4D (magenta curve). The calculated $r^2\Delta R_{\mathrm{substrate,theo}}(r)$ matches precisely with the difference between the two $r^2\Pi_{2,\mathrm{exp}}(r)$s. Therefore, we can confirm that the difference between these two $r^2\Pi_{2,\mathrm{exp}}(r)$s is caused by the substrate's structural change and the accompanying change in cage structure.

Solvent response

In a previous publication, we detailed the method for extracting solvent response following structural analysis of the PEPC-treated data.[1] The approach is straightforward: As the structural analysis determines the solute-related term, we can obtain the solvent term by subtracting the solute-related term from $\Delta S_{\mathrm{exp}}(q, t)$, the original experimental data without PEPC treatment. By implementing this method, we successfully extracted the solvent response from our experimental data. The procedure can be summarized as follows. The PEPC-treated experimental data, $\Delta S_{\mathrm{exp}}(q, t)^{\perp}$, can be represented as a linear combination of $A_{i,\mathrm{exp}}(q)^{\perp}$s as follows:

$$\Delta S_{\mathrm{exp}}(q,\ t)^{\perp} = \sum_i A_{i,\mathrm{exp}}(q)^{\perp} \cdot c_i(t) \simeq \sum_i A_{i,\mathrm{theo}}(q)^{\perp\prime} \cdot c_i(t) \tag{S26}$$

Note that Equation S26 is simply Equation S5 for the experimental data. When Equation S20 is substituted into Equation S26, the result is as follows:

$$\begin{aligned}\Delta S_{\mathrm{exp}}(q,\ t)^{\perp} &\simeq \sum_i \{A_{i,\mathrm{theo}}(q) - \gamma_{i,\mathrm{T}}(\frac{\partial S}{\partial \mathrm{T}})_{\rho} - \gamma_{i,\rho}(\frac{\partial S}{\partial \rho})_{\mathrm{T}} - \gamma_{i,\tau}\tau(q) + a_i + \frac{b_i}{q}\} \cdot c_i(t) \\ &= \sum_i \{A_{i,\mathrm{theo}}(q) + a_i + \frac{b_i}{q}\} \cdot c_i(t) + \varepsilon_{\mathrm{T}}(t)(\frac{\partial S}{\partial \mathrm{T}})_{\rho} + \varepsilon_{\rho}(t)(\frac{\partial S}{\partial \rho})_{\mathrm{T}} + \varepsilon_{\tau}(t)\tau(q)\end{aligned} \tag{S27}$$

, where $\varepsilon_{\mathrm{T}}(t)$, $\varepsilon_{\rho}(t)$, and $\varepsilon_{\tau}(t)$ refer to the contributions of $(\partial S/\partial T)_{\rho}$, $(\partial S/\partial \rho)_{T}$, and $\tau(q)$ in $\Delta S_{\mathrm{exp}}(q, t)^{\perp}$. After substituting Equation S27 into the first line of Equation S17, $\Delta S_{\mathrm{exp}}(q, t)$ can be expressed as follows:

$$
\begin{aligned}
\Delta S_{\text{exp}}(q,t) &= \Delta S_{\text{exp}}(q,t)^{\perp} + d_{\text{T}}(t)\left(\frac{\partial S}{\partial \text{T}}\right)_{\rho} + d_{\rho}(t)\left(\frac{\partial S}{\partial \rho}\right)_{\text{T}} + d_{\tau}(t)\tau(q) \\
&= \left[\sum_i \{\text{A}_{i,\text{theo}}(q) + a_i + \frac{b_i}{q}\}\cdot c_i(t) + \varepsilon_{\text{T}}(t)\left(\frac{\partial S}{\partial \text{T}}\right)_{\rho} + \varepsilon_{\rho}(t)\left(\frac{\partial S}{\partial \rho}\right)_{\text{T}} + \varepsilon_{\tau}(t)\tau(q)\right] \\
&\quad + d_{\text{T}}(t)\left(\frac{\partial S}{\partial \text{T}}\right)_{\rho} + d_{\rho}(t)\left(\frac{\partial S}{\partial \rho}\right)_{\text{T}} + d_{\tau}(t)\tau(q) \\
&= \sum_i \{\text{A}_{i,\text{theo}}(q) + a_i + \frac{b_i}{q}\}\cdot c_i(t) + \Delta\text{T}(t)\left(\frac{\partial S}{\partial \text{T}}\right)_{\rho} + \Delta\rho(t)\left(\frac{\partial S}{\partial \rho}\right)_{\text{T}} + \Delta\tau(t)\tau(q)
\end{aligned}
\tag{S28}
$$

, where $\Delta\text{T}(t)$, $\Delta\rho(t)$, and $\Delta\tau(t)$ refers to the changes in temperature, density, and contribution of the artifact over time. It is important to note that in the last line of Equation S28, the first term with the sigma symbol represents the solute-related term (with the baseline, $a_i + b_i/q$) without the PEPC treatment, and the last three terms represent the solvent term (with the artifact, $\tau(q)$). The ΔT and $\Delta\rho$ terms in the equation represent the solvent response, the change in the temperature and density of the solvent, respectively. In our analysis, we assumed that the time-profile of the artifact, $\Delta\tau$, follows that of the $\Delta\rho$, and thus $\Delta\tau = \Delta\rho$. Considering this, Equation S28 can be expressed as follows:

$$
\Delta S_{\text{exp}}(q,t) = \sum_i \{\text{A}_{i,\text{theo}}(q) + a_i + \frac{b_i}{q}\}\cdot c_i(t) + \Delta\text{T}(t)\left(\frac{\partial S}{\partial \text{T}}\right)_{\rho} + \Delta\rho(t)\left\{\left(\frac{\partial S}{\partial \rho}\right)_{\text{T}} + \tau(q)\right\} \tag{S29}
$$

Based on the above equation, we extracted $\Delta\text{T}(t)$ and $\Delta\rho(t)$ from $\Delta S_{\text{exp}}(q, t)$. Since we determined $\text{A}_{i,\text{theo}}(q)$, a_i, b_i, and $c_i(t)$ through the previous structural and kinetic analyses, and we know the solvent differentials, $(\partial S/\partial T)_{\rho}$ and $((\partial S/\partial \rho)_{T} + \tau(q))$, obtaining the remaining $\Delta\text{T}(t)$ and $\Delta\rho(t)$ is straightforward. The resulting $\Delta\text{T}(t)$ and $\Delta\rho(t)$ are shown in Figure S14 together with the shape of the solvent differentials in q-space.

Density functional theory (DFT) and time-dependent DFT (TD-DFT) calculations

The geometry optimization of the photocatalyst in both $[Ce(III)Cl_6]^{3-}$(GS) and $[Ce(IV)Cl_6]^{2-}$ was carried out using the DFT calculations. Various functional types were employed to examine the impact of different functionals used in DFT and TD-DFT calculations on the optimized geometry. The functionals used for the calculations include hybrid functionals (B3LYP and PBE0),[10-12] a range-separated functional (ωB97X),[13] and a long-range-corrected functional (CAM-B3LYP).[14] The Ce and Cl atoms were described using the Karlsruhe def2-TZVPP basis sets.[15] The unrestricted formalism was used for the structure optimization of $[Ce(III)Cl_6]^{3-}$(GS). In the TRXL experiment, a solute molecule such as $[Ce(III)Cl_6]^{3-}$ is surrounded by solvent molecules, which can affect the molecular structure of the solute. To assess the influence of the solvent (acetonitrile) on the photocatalyst's structure, we employed a Conductor-like Polarizable Continuum Model (C-PCM) and checked the difference from the structure obtained from gas phase calculations.[16-17] Additionally, we conducted scalar relativistic all-electron calculations using the zeroth order regular approximation (ZORA) to investigate the influence of relativistic effects on the photocatalyst's structures.[18-20] For the relativistic calculations, the Ce atom was described using the SARC-def2-TZVPP basis set, while the Cl atoms employed the ZORA-def2-TZVPP basis set.[21] The resolution of identity (RI) approximation with the SARC/J decontracted auxiliary basis set was used to speed up the calculations.[22-23]

For the geometry optimization of $[Ce(III)Cl_6]^{3-}$(ES), TD-DFT calculations were employed. We verified that the D_7, D_8, and D_9 states were triply degenerate states (Figure S9) and focused on optimizing the structure of the D_7 state. The same functionals used in the calculations for $[Ce(III)Cl_6]^{3-}$(GS) and $[Ce(IV)Cl_6]^{2-}$ were used. The Ce and Cl atoms were described using the def2-TZVPP basis sets, employing the RI approximation with the def2/J basis set for non-relativistic calculations.[24] We also considered the influence of the solvent on the structure of the excited state by using C-PCM and checked the difference of the structures in gas and solution phases. For the relativistic calculations, we used the SARC-def2-TZVPP basis set for the Ce atom and the ZORA-def2-TZVPP basis set for the Cl atom with RI approximation with the SARC/J decontracted auxiliary basis set.

In addition to the photocatalyst, we obtained optimized structures of 1-fluoro-4-iodobenzene (FC_6H_4I) and fluorobenzene (C_6H_5F) using DFT calculations, which were utilized for the structure refinement of the $[Ce(III)Cl_6]^{3-}$/substrate sample. We used the PBE0 hybrid functional and def2-TZVPP basis set for the calculations. To consider the effect of solvent, C-PCM solvation model was used.

The ORCA 5.0.3 software was used for the excited state calculations and relativistic calculations.[25-26] Other calculations were performed using the GAUSSIAN 16 software.[27] The optimized structures of $[Ce(III)Cl_6]^{3-}$(GS), $[Ce(IV)Cl_6]^{2-}$, and $[Ce(III)Cl_6]^{3-}$(ES) calculated under various conditions are listed in Tables S1–S3.

Molecular dynamics (MD) simulations

To calculate the scattering contribution from solvent cages nearby solute molecules, MD simulations were performed using the MOLDY 2.16e software with periodic boundary conditions.[28] The force field parameters and charges for the solvent were adapted from previous studies,[29] while the Universal force field (UFF) parameters were used for the solute molecules.[30] The natural bonding orbital (NBO) atomic charges obtained from DFT calculations employing the PBE0 hybrid functional were used for solute molecules. All simulations were performed at 298 K and a density of 0.786 g/cm^3. A solute molecule was embedded in a box containing 512 solvent molecules. The simulation box was equilibrated using a Nose-Hoover thermostat, and the trajectories were followed for up to 1 ns with a 0.5 fs time step. The pair distribution functions were calculated from the simulated trajectories and used to calculate the scattering intensity of the solvent cages according to a well-established procedure.[7]

Mechanism of substrate reduction by the photoexcited catalyst, $[Ce(III)Cl_6]^{3-}$(ES)

We would like to emphasize that the observed time constant (500 ± 20 ps) for the oxidation of oxidation of $[Ce(III)Cl_6]^{3-}$(ES), or the reduction of the substrate, in the $[Ce(III)Cl_6]^{3-}$/substrate sample is notably small. To underscore this point, we can compare this time constant to the theoretical upper limit of the rate constant for a diffusion-limited reaction occurring in acetonitrile solvent. The theoretical value can be calculated using the following equation:

$$k_{\text{diff}} = \frac{4\pi N_A}{1000}(R_A + R_B)(D_A + D_B) \quad \text{(S30)}$$

, where N_A is Avogadro's number, R_X is the molecular radius of X, and D_X is the diffusion coefficient of X. The radii of $[Ce(III)Cl_6]^{3-}$ and 1-fluoro-4-iodobenzene used for the calculation were estimated to be 4.5 and 3.4 Å,

based on the crystal structure and the longest axis of the molecular structure, respectively.[3] The diffusion coefficients for $[Ce(III)Cl_6]^{3-}$ and 1-fluoro-4-iodobenzene were estimated to be 1.43×10^{-5} and 1.89×10^{-5} cm^2s^{-1}, respectively, using Stokes-Einstein equation using a viscosity of 0.34 cP.[3] It is worth noting that the diffusion rate constant is influenced by the viscosity of the solvent, which, in turn, is greatly affected by the temperature at which the experiments are conducted. At room temperature (25 °C), the viscosity of acetonitrile is approximately 0.34 cP, whereas at around 15 °C, it can increase by approximately 10% to reach 0.38 cP. With the given diffusion rate constant, the time constant for the reaction between $[Ce(III)Cl_6]^{3-}$ and F–Ph–I is estimated to be 330 ps, assuming a pseudo-first-order reaction and a concentration of 150 mM for F–Ph–I. This time constant aligns well with our experimental results. According to a study, the overall reaction rate constant (k_{obs}) for outer-sphere electron transfer can be expressed as follows:[31]

$$k_{obs} = \frac{k_{diff} \cdot k_{et}}{k_{-d} + k_{et}} \quad \text{(S31)}$$

Here, k_{diff} is the rate constant for forming an encounter complex through the diffusion of reactants, k_{-d} is that of dissociation of the complex, and k_{et} is that of electron transfer within the complex. When k_{-d} is much smaller than k_{et}, indicating that dissociation is significantly slower than the electron transfer, k_{obs} can be simplified to k_{diff}. This outcome suggests that if the electron transfer between $[Ce(III)Cl_6]^{3-}$ and 1-fluoro-4-iodobenzene occurs in an outer-sphere manner and electron transfer occurs much faster than the dissociation of the complex, the overall time constant for the reaction can be similar to the one obtained by assuming a diffusion limited reaction. Furthermore, it is worth noting that the effective radii of molecules (R) in solution are often larger than those obtained from crystal structures.[32] An increase in the radii of the molecules leads to an increase in the calculated reaction rate and a decrease in the calculated time constant, falling below 300 ps. Considering these factors, the observed time constant of 500 ps in this study is considered a reasonable value.

In addition, we investigated the possibility of the formation of a pre-associated complex between the photocatalyst and substrate, which can lead to the fast electron transfer. Firstly, the UV-visible spectrum of the reaction mixture (photocatalyst and substrate) was compared with that of photocatalyst. In cases where a pre-associated complex is formed due to some interactions between the photocatalyst and substrate, such interaction can influence the electronic structure of the photocatalyst. Consequently, the absorption spectrum of the photocatalyst with and without the presence of the substrate would exhibit different features. The effect of the presence of substrate on the absorption around 330 nm, in which the absorption of the photocatalyst is maximum, was small (Figure S4A). This observation lends some support to the exclusion of the possibility of a pre-associated complex formation between the catalyst and the substrate.

Furthermore, if a pre-associated complex had already existed in the solution before the photoexcitation of the catalyst took place, it should have left a distinctive signature in the TRXL data. Specifically, since the substrate contains a heavy I atom, the formation of a pre-associated complex should result in a clear oscillatory feature corresponding to the distance distribution between the Ce atom of the catalyst and the I atom of the substrate. For instance, we calculated theoretical difference scattering curves of the solute-only signals for two scenarios. In the first scenario, it was assumed that the photocatalyst and substrate undergoing the C–I bond activation are separate in the ground state ($[Ce(III)Cl_6]^{3-}$(GS) + (F–Ph–I) → $[Ce(IV)Cl_6]^{2-}$ + F–Ph + I^-). The other scenario involves a pre-associated complex where structural changes occur as follows: $[Ce(III)Cl_6]^{3-}$(GS)···(F–

Ph–I) → $[Ce(IV)Cl_6]^{2-}$ + F–Ph + I^-, with the Ce–I distance of 6 Å. Here, "···" indicates the presence of a weak interaction, indicative of the formation of a pre-associated species. Then, the PEPC-treated difference scattering curves obtained by assuming two scenarios were compared with $qA_2(q)^{\perp}$ as shown in Figure S15. It is evident that the theoretical curves generated considering the formation of pre-associated species before photoexcitation do not align with the experimental data compared to that generated assuming the absence of the pre-associated species. Nevertheless, this does not exclude a transient complex formation occurring after photoexcitation, which is expected to happen.

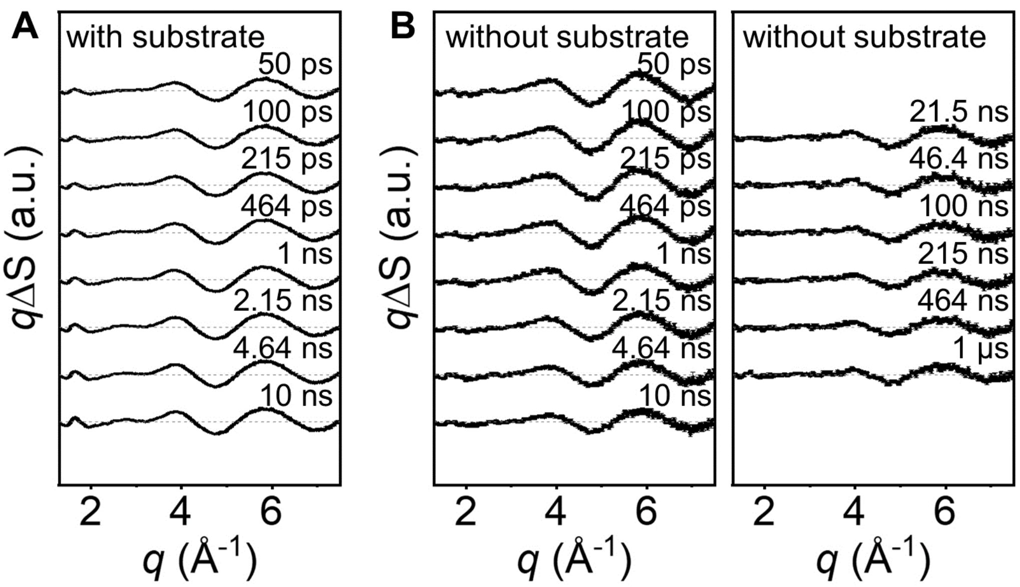

Figure S1. TRXL data after the PEPC treatment. (A) PEPC-treated TRXL data for the $[Ce(III)Cl_6]^{3-}$/substrate sample. (B) PEPC-treated TRXL data for the $[Ce(III)Cl_6]^{3-}$-only sample. The data are presented as mean values ± standard errors of the mean (SEM).

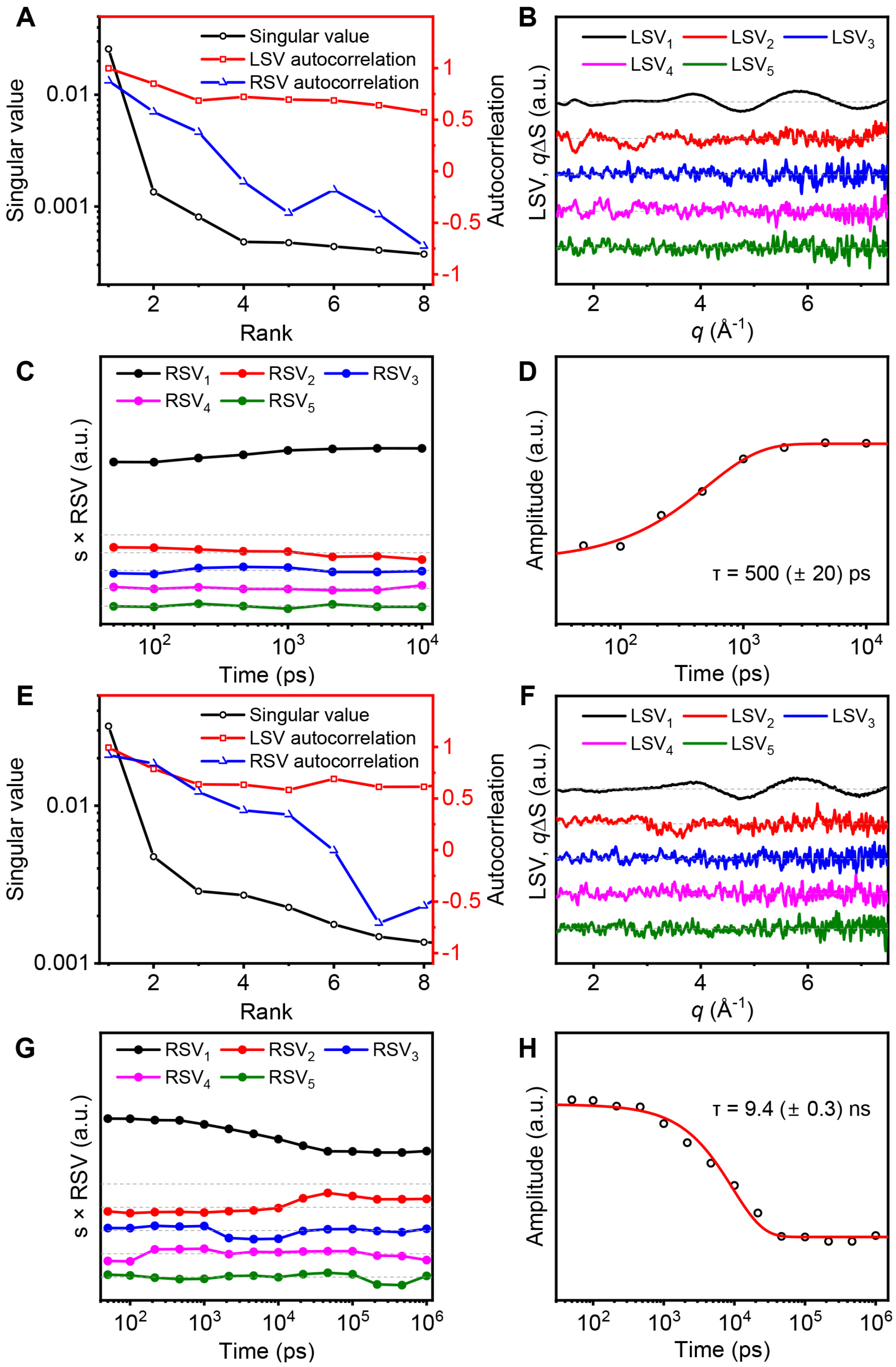

Figure S2. SVD analysis of the PEPC-treated TRXL data of $[Ce(III)Cl_6]^{3-}$. (A, E) Singular values (black circle), autocorrelation values of LSVs (red square), and autocorrelation values of RSVs (blue triangle) for the $[Ce(III)Cl_6]^{3-}$/substrate (A) and $[Ce(III)Cl_6]^{3-}$-only (E) samples. (B, F) The first five LSVs obtained from TRXL data of the $[Ce(III)Cl_6]^{3-}$/substrate (B) and $[Ce(III)Cl_6]^{3-}$-only (F) samples. (C, G) The first five RSVs obtained from TRXL data of the $[Ce(III)Cl_6]^{3-}$/substrate (C) and $[Ce(III)Cl_6]^{3-}$-only (G) samples. The RSVs are weighted by their singular values to visualize their contributions to the TRXL data. (D, H) The fit (red solid line) of the 1st RSV (black circle) with a sum of a constant and an exponential function for the $[Ce(III)Cl_6]^{3-}$/substrate (D) and

$[Ce(III)Cl_6]^{3-}$-only (H) samples. The fitted time constant is shown in each panel. The singular values, autocorrelation values, and overall features of the LSVs and RSVs indicate that only one singular vector significantly contributes to the TRXL data for both samples.

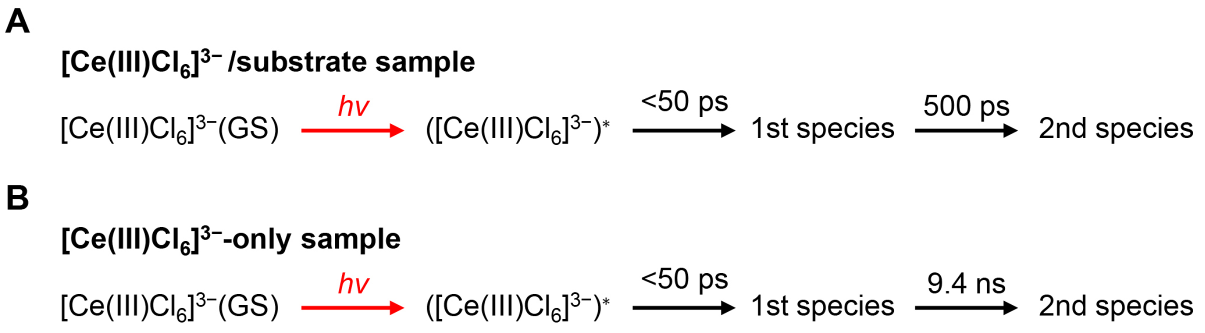

Figure S3. Schematics of the kinetic models employed to describe the TRXL data. The kinetic models used for the data obtained from two samples are depicted: $[Ce(III)Cl_6]^{3-}$/substrate sample (A) and $[Ce(III)Cl_6]^{3-}$-only sample (B).

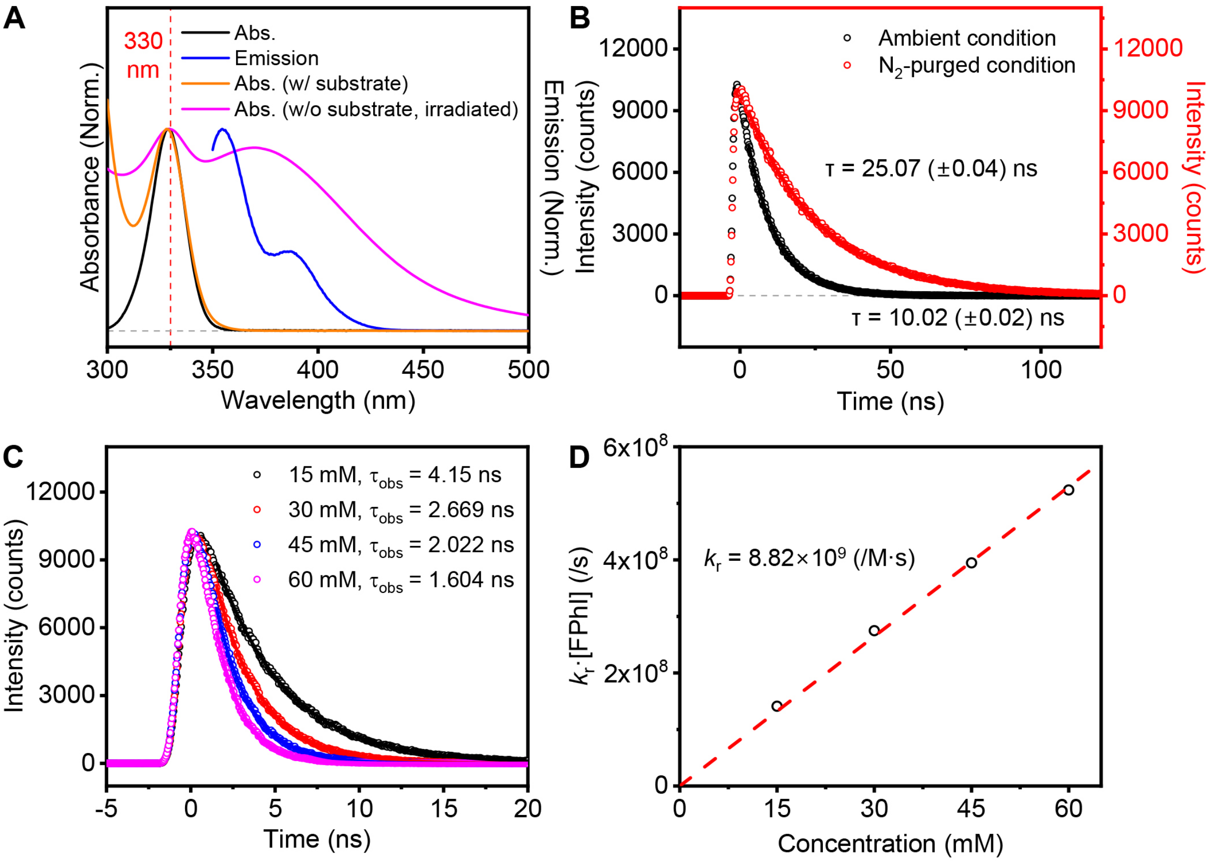

Figure S4. Spectroscopic characterization of $[Ce(III)Cl_6]^{3-}$ in acetonitrile. (A) Normalized UV-visible absorption spectra and emission spectrum of $[Ce(III)Cl_6]^{3-}$. UV-visible absorption spectra and emission spectrum of $[Ce(III)Cl_6]^{3-}$ before LED irradiation are shown as black and blue solid lines, respectively. The UV-visible absorption spectrum of the photocatalyst with the substrate is shown in orange, while that of the photocatalyst without the substrate after LED irradiation (340 nm) for 1 hour under ambient conditions with oxygen is shown in magenta. The UV-visible spectra other than that of the photocatalyst with the substrate were normalized so that the maximum absorptions were set to be 1. The UV-visible absorption spectrum of the photocatalyst with the substrate was normalized so that the absorption peak around ~330 nm is set to be 1. The excitation wavelength used for the TRXL experiment is marked with a red dashed line. (B) TCSPC profiles of $[Ce(III)Cl_6]^{3-}$ under the ambient condition (black dots) and the N_2-purged condition (red dots). The fits for the experimental data are shown as black and red solid lines for the ambient condition and the N_2-purged condition, respectively. The lifetimes of the emission were 10.02 and 25.07 ns for ambient and N_2-purged conditions, respectively. (C) TCSPC profiles of $[Ce(III)Cl_6]^{3-}$ under ambient conditions with varying substrate concentrations, along with their corresponding theoretical fits. The TCSPC profiles for substrate concentrations of 15, 30, 45, and 60 mM are shown with black, red, blue, and magenta dots, respectively, while the solid lines indicate the fitted curves. (D) Pseudo-first order rate constants for the reaction between the photocatalyst and substrate ($k_r \cdot$ [FPhI]), as determined across different substrate concentrations, and the bimolecular rate constant (k_r) obtained through fitting. The pseudo-first order rate constants were derived from the emission lifetimes measured for samples with various substrate

concentrations. The bimolecular rate constant was determined by fitting the pseudo-first order rate constants using a linear function.

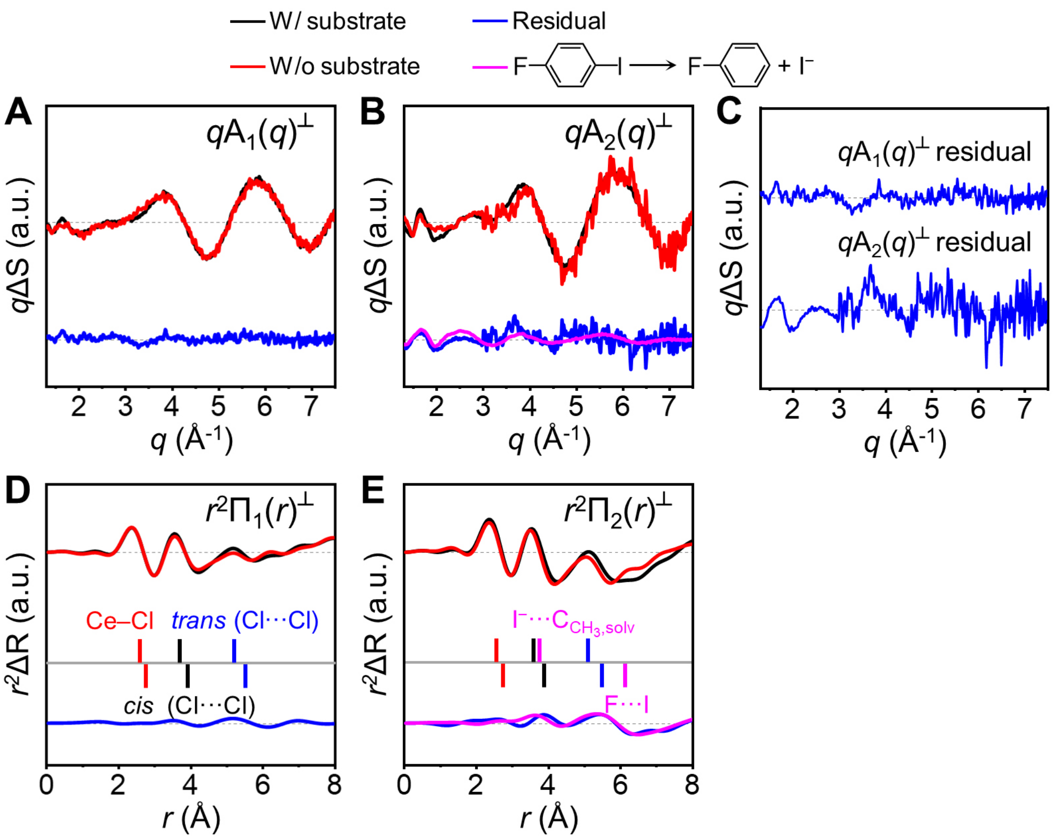

Figure S5. Contribution of the reaction of the substrate, 1-fluoro-4-iodobenzene (F–Ph–I), to the PEPC-treated SADSs of the $[Ce(III)Cl_6]^{3-}$/substrate data. This figure is the PEPC-treated counterpart of Figure 4. (A) The $qA_1(q)^{\perp}$s of $[Ce(III)Cl_6]^{3-}$/substrate and $[Ce(III)Cl_6]^{3-}$-only data. (B) The $qA_2(q)^{\perp}$s of $[Ce(III)Cl_6]^{3-}$/substrate and $[Ce(III)Cl_6]^{3-}$-only data. (C) Comparison of the residuals of $qA_1(q)^{\perp}$s and $qA_2(q)^{\perp}$s. (D) The $r^2\Pi_1(r)^{\perp}$s of $[Ce(III)Cl_6]^{3-}$/substrate and $[Ce(III)Cl_6]^{3-}$-only data. (E) The $r^2\Pi_2(r)^{\perp}$s of $[Ce(III)Cl_6]^{3-}$/substrate and $[Ce(III)Cl_6]^{3-}$-only data. The $r^2\Pi_1(r)^{\perp}$ and $r^2\Pi_2(r)^{\perp}$ curves were obtained by performing Fourier sine transforms on $qA_1(q)^{\perp}$ and $qA_2(q)^{\perp}$, respectively. Since the distortion due to PEPC is present in $qA_1(q)^{\perp}$ and $qA_2(q)^{\perp}$, $r^2\Pi_1(r)^{\perp}$ and $r^2\Pi_2(r)^{\perp}$ are less exact than $r^2\Pi_1(r)$ and $r^2\Pi_2(r)$ presented in Figure 4. This is the reason why $qA_1(q)$ and $qA_2(q)$ were obtained from $qA_1(q)^{\perp}$ and $qA_2(q)^{\perp}$ after correcting the distortion due to the PEPC treatment and used to obtain $r^2\Pi_1(r)$ and $r^2\Pi_2(r)$ by performing Fourier sine transform. In all panels, the curves for $[Ce(III)Cl_6]^{3-}$/substrate are shown in black, and those of $[Ce(III)Cl_6]^{3-}$-only are shown in red. For comparison, the scales of the curves for $[Ce(III)Cl_6]^{3-}$-only and $[Ce(III)Cl_6]^{3-}$/substrate were adjusted. In all panels, the residual obtained by subtracting the curve of $[Ce(III)Cl_6]^{3-}$-only from that of $[Ce(III)Cl_6]^{3-}$/substrate is shown in blue. The residuals for $qA_1(q)^{\perp}$ in (A) and $r^2\Pi_1(r)^{\perp}$ in (D) are negligible, indicating that the SADSs of $[Ce(III)Cl_6]^{3-}$/substrate and $[Ce(III)Cl_6]^{3-}$-only are identical in both q- and r-spaces. In contrast, the residuals for $qA_2(q)^{\perp}$ in (B) and $r^2\Pi_2(r)^{\perp}$ in (E) are not negligible and exhibit a distinct feature, indicating that the SADS of $[Ce(III)Cl_6]^{3-}$/substrate in q- and r-spaces are different from those of $[Ce(III)Cl_6]^{3-}$-only. For a clear comparison, the residuals in (A) and (B) are compared in (C). The residual for $qA_1(q)^{\perp}$ is negligible, indicating that the $qA_1(q)^{\perp}$ for both the $[Ce(III)Cl_6]^{3-}$-only and $[Ce(III)Cl_6]^{3-}$/substrate samples accounts for the same process. In contrast, the residual for $qA_2(q)^{\perp}$ is not negligible, indicating that the $qA_2(q)^{\perp}$ for the $[Ce(III)Cl_6]^{3-}$/substrate sample is different from that for the $[Ce(III)Cl_6]^{3-}$-only sample. In fact, these residuals can be explained by the theoretical $q\Delta S(q)^{\perp}$

due to the reaction of the substrate (shown in magenta) in (B) and its $r^2\Delta R(r)^{\perp}$ (shown in magenta) in (E). The theoretical difference scattering curve of the reaction of the substrate was calculated considering the dissociation of F–Ph–I to fluorobenzene (F–Ph) and iodide ion (I^-). In (D) and (E), the vertical bars indicate the distances between the Ce and Cl atoms (red), those between the Cl atoms in *cis* position (black), and those between the Cl atoms in *trans* position (blue) of the photocatalyst. The magenta vertical bars indicate the distances between the atoms related to the reaction of the substrate. F···I indicates the distance between F and I atoms in the substrate, and $I^-\cdots C_{CH_3,solv}$ indicates that between the iodide ion and C atom of the methyl group in an acetonitrile molecule. The vertical bars above the gray solid line indicate the distances in the products ($[Ce(III)Cl_6]^{3-}$(ES), $[Ce(IV)Cl_6]^{2-}$, F–Ph, and I^-), whereas those below the gray solid line indicate the distances in the reactants ($[Ce(III)Cl_6]^{3-}$(GS) and F–Ph–I).

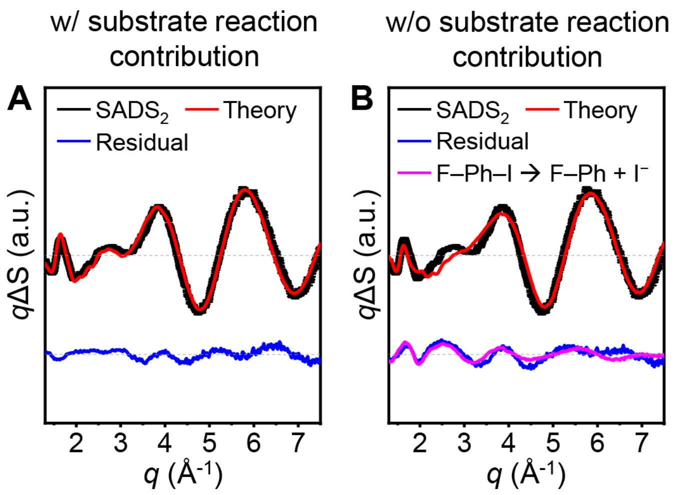

Figure S6. Effect of the contribution of the reaction of the substrate to the structure refinement of the $[Ce(III)Cl_6]^{3-}$/substrate data. The PEPC-treated experimental $SADS_2$ (black, $qA_{2,exp}(q)^{\perp}$) is superimposed with the theoretical PEPC-treated SADS (red, $qA_{2,theo}(q)^{\perp}$). The PEPC-treated SADSs are presented as mean values ± SEM. In (A), the contribution of the reaction of the substrate was included during the calculation of the $qA_{2,theo}(q)^{\perp}$, whereas it was neglected in (B). The residual, obtained by subtracting the $qA_{2,theo}(q)^{\perp}$ from the $qA_{2,exp}(q)^{\perp}$ is shown in blue in each panel. The residual is negligible in (A) whereas it is not negligible in (B). The residual in (B) matches the PEPC-treated theoretical difference scattering curve due to the reaction of the substrate shown in magenta. The theoretical difference scattering curve of the reaction of the substrate was calculated considering the dissociation of 1-fluoro-4-iodobenzene (F–Ph–I) to fluorobenzene (F–Ph) and iodide ion (I^-).

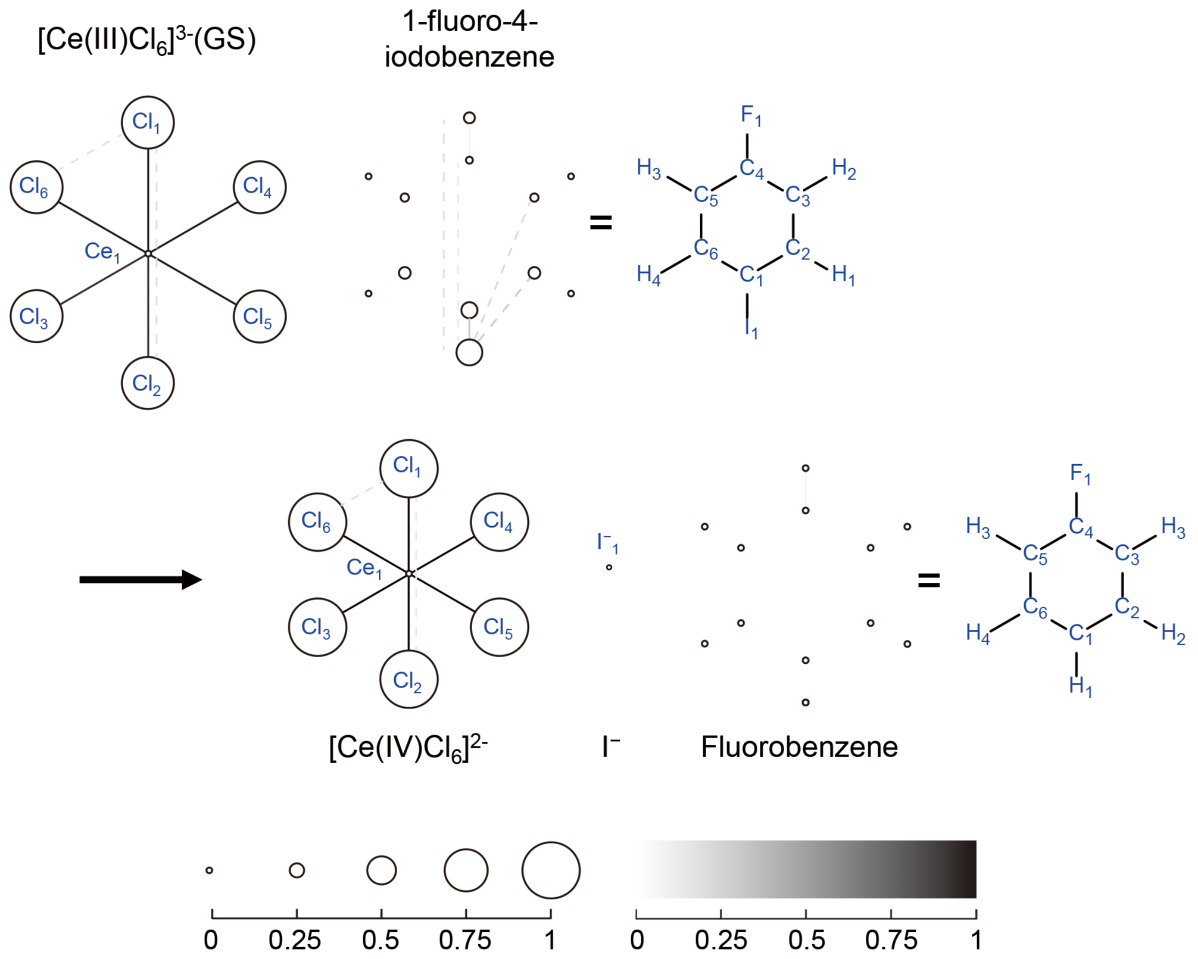

Figure S7. Sensitivity plot for the reaction of $[Ce(III)Cl_6]^{3-}$(GS) + 1-fluoro-4-iodobenzene → $[Ce(IV)Cl_6]^{2-}$ + I^- + fluoroiodobenzene. A nuclear position displacement of 0.1 Å was used to estimate the atomic position sensitivity, and the internuclear distance change of 0.05 Å was used to estimate the sensitivity of the internuclear distance. A larger radius of an atom indicates a greater sensitivity of the atomic position, and a darker color of an internuclear distance indicates a greater sensitivity of the internuclear distance. Internuclear distances between atoms having chemical bonds are shown with solid lines, while those between atoms without chemical bonds are shown with dashed lines. For simplicity, the internuclear distances with the sensitivities below a threshold are omitted. When multiple internuclear distances between atoms without chemical bonds have the same sensitivity (for example, Cl_1–Cl_6 and Cl_1–Cl_4 in $[Ce(III)Cl_6]^{3-}$(GS)), only one of them is shown for simplicity. The radii of atoms with sensitivities below a threshold are set to the minimum value for clear visualization. The q range from 1.3 to 7.5 $Å^{-1}$ was used to calculate the sensitivities of the atomic positions and internuclear distances.

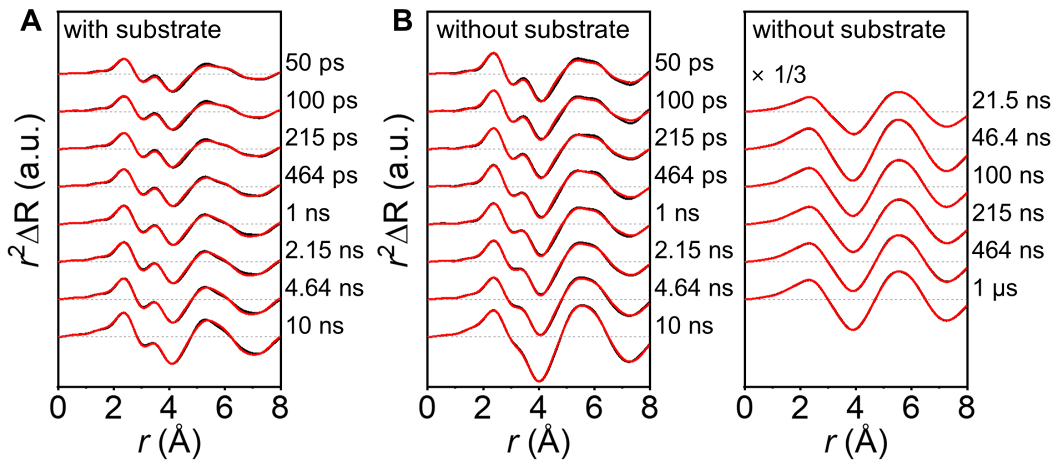

Figure S8. Difference radial distribution functions of the $[Ce(III)Cl_6]^{3-}$/substrate and $[Ce(III)Cl_6]^{3-}$-only samples. (A, B) The experimental (black) and theoretical (red) difference radial distribution functions ($r^2\Delta R(r)$s) of the $[Ce(III)Cl_6]^{3-}$/substrate (A) and $[Ce(III)Cl_6]^{3-}$-only (B) samples. The $r^2\Delta R(r)$s were obtained by performing the Fourier sine transform on the difference scattering curves ($q\Delta S(q)$s) shown in Figure 2. For clarity, the $r^2\Delta R(r)$s of the $[Ce(III)Cl_6]^{3-}$-only sample at late time delays are scaled down by three.

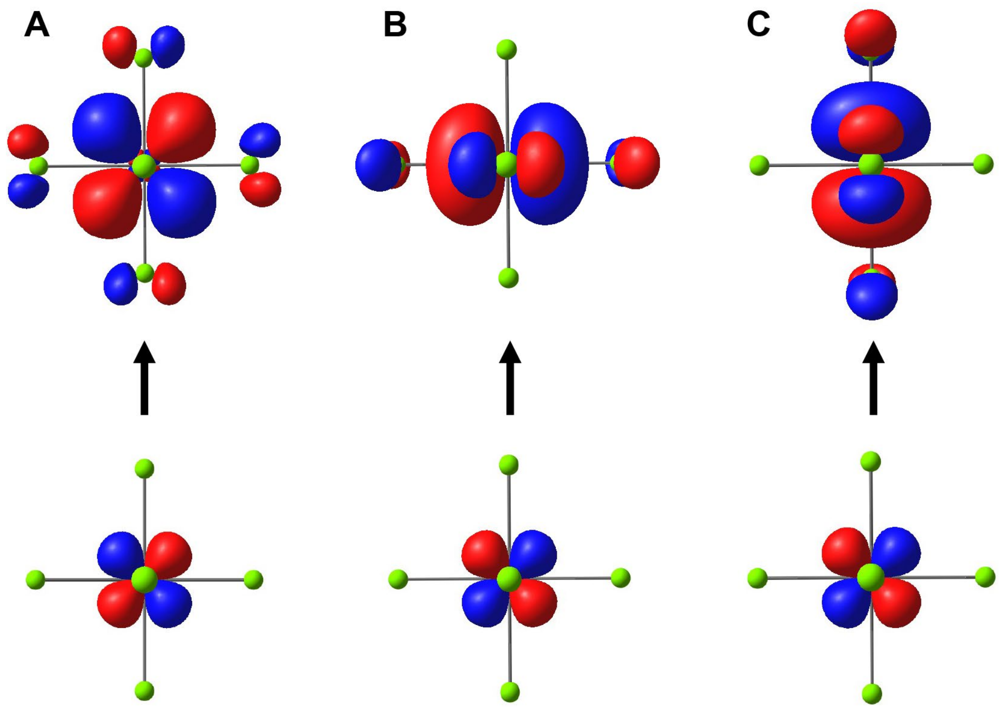

Figure S9. Natural transition orbitals (NTOs) of the triply degenerate T_{2g} states. NTOs for the transition from the ground state to the D_7, D_8, and D_9 states are shown in (A), (B), and (C), respectively. The cerium atom, which is obscured by a chlorine atom, and chlorine atoms are shown in yellow and green, respectively.

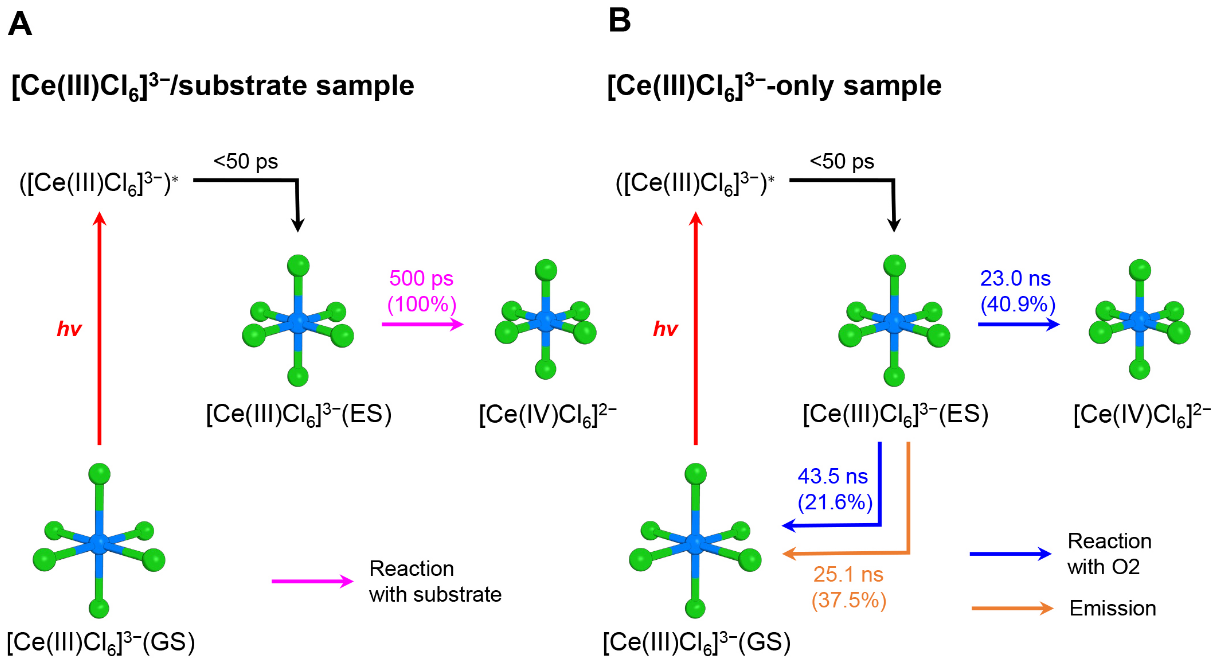

Figure S10. Schematics of the photoreaction pathways for $[Ce(III)Cl_6]^{3-}$/substrate and $[Ce(III)Cl_6]^{3-}$-only samples. The reaction pathways for $[Ce(III)Cl_6]^{3-}$/substrate (A) and $[Ce(III)Cl_6]^{3-}$-only (B) samples are shown, along with the time constants for each reaction step and the fraction of molecules participating in each reaction step. Upon photoexcitation, a population of the ground state, $[Ce(III)Cl_6]^{3-}$(GS), is excited in the Franck-Condon region, $([Ce(III)Cl_6]^{3-})^*$, which could not be captured due to our time resolution (~50 ps). The population at $([Ce(III)Cl_6]^{3-})^*$ transforms into the stable structure of the excited state, ($[Ce(III)Cl_6]^{3-}$(ES)), that is identified by TRXL. In the presence of the substrate, all the $[Ce(III)Cl_6]^{3-}$(ES) generated at 50 ps undergo a reaction with the substrate, forming the oxidized state, $[Ce(IV)Cl_6]^{2-}$. In the absence of the substrate, $[Ce(III)Cl_6]^{3-}$(ES) undergoes transformation via multiple reaction pathways. Only a fraction of $[Ce(III)Cl_6]^{3-}$(ES) generated at 50 ps undergoes electron transfer to oxygen, leading to the formation of $[Ce(IV)Cl_6]^{2-}$. The remaining fraction decays to the ground state through either emission or energy transfer to oxygen. In the schematics, the photoexcitation process, the reaction with the substrate, the reactions with oxygen (both electron transfer and energy transfer), and the emissive decay are depicted in red, magenta, blue, and orange arrows, respectively. The parentheses next to the arrows indicate the fraction of the molecules participating in each reaction relative to the population of $[Ce(III)Cl_6]^{3-}$(ES) at 50 ps. In the schematic, the cerium and chloride atoms are shown in blue and green, respectively.

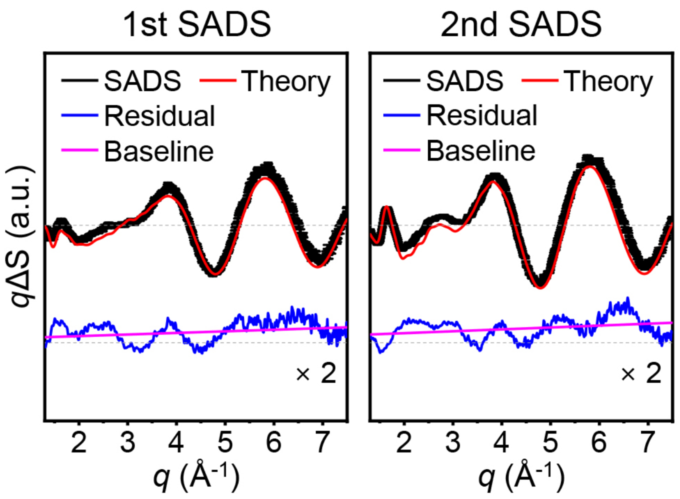

Figure S11. Structure refinement of the SADSs from the $[Ce(III)Cl_6]^{3-}$/substrate data without considering the contribution of the baseline. The experimental PEPC-treated SADSs and theoretical PEPC-treated SADSs without considering the contribution of the baselines are shown in black and red, respectively. The PEPC-treated SADSs are presented as mean values ± SEM. The residuals, obtained by subtracting the theoretical PEPC-treated SADSs from the experimental PEPC-treated SADS, are shown in blue. The magenta lines indicate the baselines with a linear shape. For clarity, the residuals and the baselines are scaled up by two.

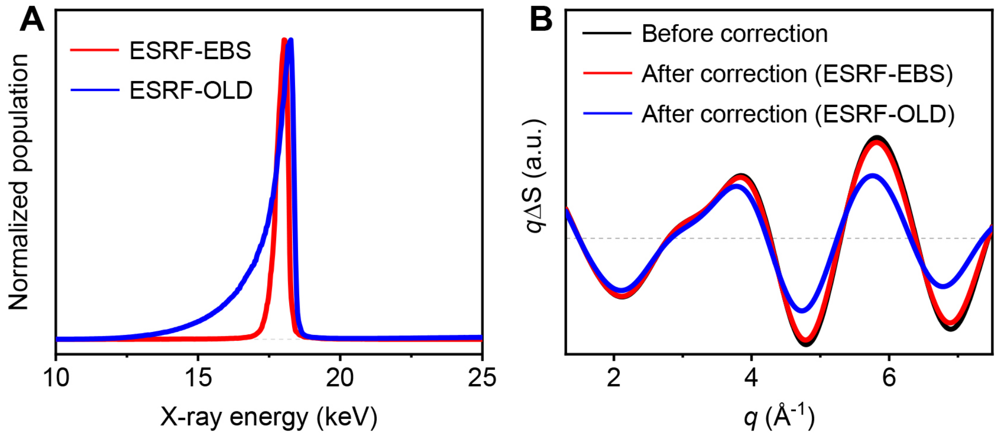

Figure S12. Effect of X-ray beam polychromaticity on the broadening of difference scattering curves. (A) The energy spectra of pink beams, corresponding to ESRF-EBS (red) and ESRF-OLD (blue), are compared. (B) Comparison of difference scattering curves for $[Ce(III)Cl_6]^{3-}$(ES) under three conditions: monochromatic X-ray beam (black), polychromatic pink beam from ESRF-EBS (red), and pink beam from ESRF-OLD (blue).

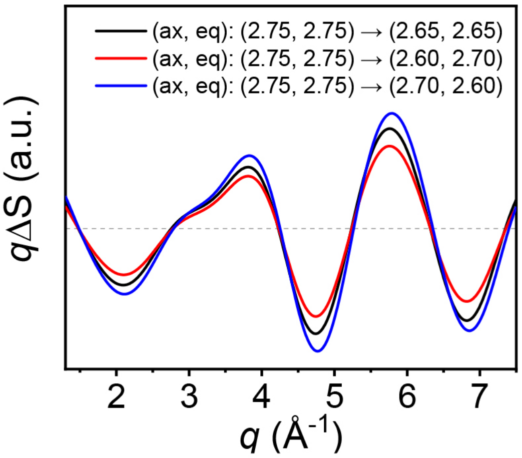

Figure S13. Sensitivity of TRXL signal to different types of structural changes in $[Ce(III)Cl_6]^{3-}$. To showcase the capabilities of TRXL in discriminating different types of structural changes, we simulated the TRXL signal corresponding to three different scenarios: (i) symmetric contraction (black), (ii) asymmetric contraction, with a larger contraction in the axial Ce–Cl bonds (red), and (iii) asymmetric contraction, with a larger contraction in the equatorial Ce–Cl bonds (blue). For (i), both axial and equatorial distances were reduced by 0.1 Å. For (ii) and (iii), axial and equatorial distances were reduced by 0.15 Å and 0.05 Å (ii) and 0.05 Å and 0.15 Å (iii), respectively. Each structural change yields a unique TRXL signal with characteristic shape and amplitude. The overall shapes of the signals may appear similar, but upon closer inspection, it is evident that the positions of the peaks vary slightly. This distinctive signal provides a robust means to analyze and differentiate different types of structural changes, such as symmetric or asymmetric contraction of the Ce–Cl bonds.

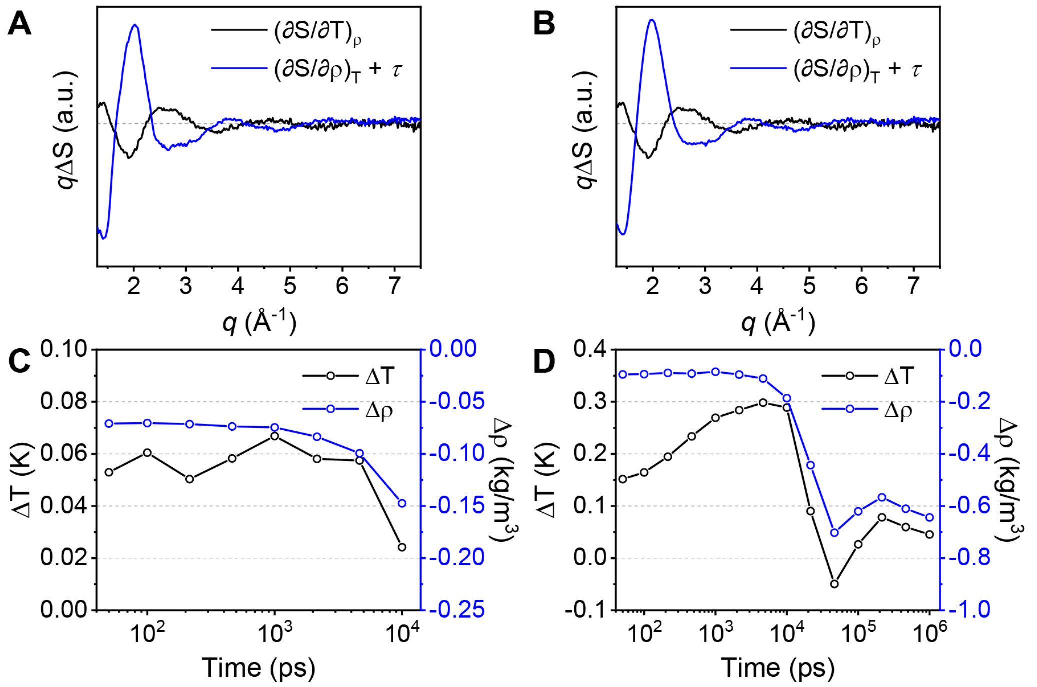

Figure S14. Solvent response. (A, B) The solvent differentials, $(\partial S/\partial T)_\rho$ (black) and the sum of $(\partial S/\partial \rho)_T$ and artifact (τ) (blue), for the $[Ce(III)Cl_6]^{3-}$/substrate (A) and the $[Ce(III)Cl_6]^{3-}$-only samples (B). (C, D) The changes in temperature (ΔT) and density (Δρ) for the $[Ce(III)Cl_6]^{3-}$/substrate (C) and the $[Ce(III)Cl_6]^{3-}$-only samples (D) obtained from the analysis of TRXL data. The analysis of solvent response is described in the "Solvent response" section in the SI.

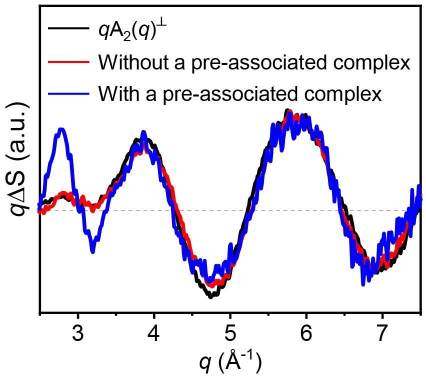

Figure S15. Comparison of $qA_2(q)^{\perp}$ of the $[Ce(III)Cl_6]^{3-}$/substrate sample with theoretical difference scattering curves, depicting scenarios with and without the presence of a pre-associated complex between the photocatalyst and substrate in the ground state. $qA_2(q)^{\perp}$ is shown in black, while the theoretical difference scattering curves assuming the absence and presence of the pre-associated complex are shown in red and blue, respectively. For the case where the pre-associated complex is present, it was assumed that the Ce–I distance in the pre-associated complex was 6 Å. The theoretical difference scattering curves were generated by performing PEPC on the solute-only signals.

Table S1. DFT optimized Ce–Cl bond lengths (Å) of $[Ce(III)Cl_6]^{3-}$(GS).

$[Ce(III)Cl_6]^{3-}$(GS) (Ground state)	Gas Phase	C-PCM	ZORA/C-PCM
B3LYP	2.878 Å	2.836 Å	2.815 Å
PBE0	2.844 Å	2.794 Å	2.783 Å
ωB97X	2.854 Å	2.815 Å	2.799 Å
CAM-B3LYP	2.858 Å	2.816 Å	2.799 Å

Table S2. DFT optimized Ce–Cl bond lengths (Å) of $[Ce(IV)Cl_6]^{2-}$.

$[Ce(IV)Cl_6]^{2-}$ (Oxidized state)	Gas Phase	C-PCM	ZORA/C-PCM
B3LYP	2.670 Å	2.661 Å	2.654 Å
PBE0	2.634 Å	2.623 Å	2.621 Å
ωB97X	2.645 Å	2.636 Å	2.632 Å
CAM-B3LYP	2.648 Å	2.638 Å	2.629 Å

Table S3. TD-DFT optimized Ce–Cl bond lengths (Å) of $[Ce(III)Cl_6]^{3-}$(ES).

$[Ce(III)Cl_6]^{3-}$(ES) (Excited state)	Gas Phase		C-PCM		ZORA/C-PCM	
	Axial Ce–Cl	Equatorial Ce–Cl	Axial Ce–Cl	Equatorial Ce–Cl	Axial Ce–Cl	Equatorial Ce–Cl
B3LYP[a]	-	-	2.692 Å	2.775 Å	2.697 Å	2.776 Å
PBE0	2.722 Å	2.794 Å	2.663 Å	2.735 Å	2.670 Å	2.736 Å
ωB97X	2.730 Å	2.837 Å	2.677 Å	2.770 Å	2.684 Å	2.776 Å
CAM-B3LYP	2.735 Å	2.830 Å	2.678 Å	2.763 Å	2.683 Å	2.766 Å

[a]The structure of $[Ce(III)Cl_6]^{3-}$(ES) with the B3LYP functional in the gas phase could not be obtained due to the root flipping problem during the geometry optimization step.

Table S4. Effect of using inaccurate experimental parameters for analysis to the structural parameters.

Methods	$[Ce(III)Cl_6]^{3-}$(GS) (Ground state)		$[Ce(III)Cl_6]^{3-}$(ES) (Excited state)		$[Ce(IV)Cl_6]^{2-}$ (Oxidized state)	
	Axial Ce–Cl (Å)	Equatorial Ce–Cl (Å)	Axial Ce–Cl (Å)	Equatorial Ce–Cl (Å)	Axial Ce–Cl (Å)	Equatorial Ce–Cl (Å)
Reference	2.753	2.753	2.593	2.593	2.561	2.561
Sample-to-detector distance increase by 1 mm	2.798 (+1.63%)	2.798 (+1.63%)	2.630 (+1.43%)	2.630 (+1.43%)	2.600 (+1.52%)	2.600 (+1.52%)
Sample-to-detector distance increase by 0.5 mm	2.774 (+0.76%)	2.774 (+0.76%)	2.612 (+0.73%)	2.612 (+0.73%)	2.582 (+0.82%)	2.582 (+0.82%)
Sample-to-detector distance decrease by 0.5 mm	2.732 (-0.76%)	2.732 (-0.76%)	2.574 (-0.73%)	2.574 (-0.73%)	2.540 (-0.82%)	2.540 (-0.82%)
Sample-to-detector distance decrease by 1 mm	2.713 (-1.45%)	2.713 (-1.45%)	2.554 (-1.50%)	2.554 (-1.50%)	2.519 (-1.64%)	2.519 (-1.64%)
X-ray energy increase by 1%	2.725 (-1.02%)	2.725 (-1.02%)	2.567 (-1.00%)	2.567 (-1.00%)	2.533 (-1.09%)	2.533 (-1.09%)
X-ray energy increase by 0.5%	2.739 (-0.51%)	2.739 (-0.51%)	2.580 (-0.50%)	2.580 (-0.50%)	2.547 (-0.55%)	2.547 (-0.55%)
X-ray energy decrease by 0.5%	2.767 (+0.51%)	2.767 (+0.51%)	2.606 (+0.50%)	2.606 (+0.50%)	2.576 (+0.59%)	2.576 (+0.59%)
X-ray energy decrease by 1%	2.782 (+1.05%)	2.782 (+1.05%)	2.619 (+1.00%)	2.619 (+1.00%)	2.589 (+1.09%)	2.589 (+1.09%)

The values in the parentheses indicate the deviation from the reference values.

Table S5. Comparison of structural parameters for $[Ce(III)Cl_6]^{3-}$(GS), $[Ce(III)Cl_6]^{3-}$(ES), and $[Ce(IV)Cl_6]^{2-}$ obtained from the analysis without and with considering polychromatic correction.

Methods	$[Ce(III)Cl_6]^{3-}$(GS) (Ground state)		$[Ce(III)Cl_6]^{3-}$(ES) (Excited state)		$[Ce(IV)Cl_6]^{2-}$ (Oxidized state)	
	Axial Ce–Cl (Å)	Equatorial Ce–Cl (Å)	Axial Ce–Cl (Å)	Equatorial Ce–Cl (Å)	Axial Ce–Cl (Å)	Equatorial Ce–Cl (Å)
Without polychromatic correction	2.753	2.753	2.593	2.593	2.561	2.561
With polychromatic correction	2.745 (-0.29%)	2.745 (-0. 29%)	2.592 (-0.04%)	2.592 (-0.04%)	2.561 (0%)	2.561 (0%)

The values in the parentheses indicate the deviation from the bond distances obtained from the analysis without considering the polychromatic correction.

Table S6. Structural parameters for $[Ce(III)Cl_6]^{3-}$(GS), $[Ce(III)Cl_6]^{3-}$(ES), and $[Ce(IV)Cl_6]^{2-}$ obtained from the analysis using the projection to extract the perpendicular component (PEPC) and global fitting analysis (GFA).

Methods	$[Ce(III)Cl_6]^{3-}$(GS) (Ground state)		$[Ce(III)Cl_6]^{3-}$(ES) (Excited state)		$[Ce(IV)Cl_6]^{2-}$ (Oxidized state)	
	Axial Ce–Cl (Å)	Equatorial Ce–Cl (Å)	Axial Ce–Cl (Å)	Equatorial Ce–Cl (Å)	Axial Ce–Cl (Å)	Equatorial Ce–Cl (Å)
PEPC	2.753 ± 0.002	2.753 ± 0.001	2.593 ± 0.005	2.593 ± 0.003	2.561 ± 0.003	2.561 ± 0.001
GFA	2.757 ± 0.002	2.757 ± 0.001	2.593 ± 0.005	2.593 ± 0.003	2.560 ± 0.003	2.560 ± 0.001

Reference

1. Ki, H.; Gu, J.; Cha, Y.; Lee, K. W.; Ihee, H., Projection to extract the perpendicular component (PEPC) method for extracting kinetics from time-resolved data. *Struct. Dyn.* **2023,** *10* (3), 034103.
2. Costanzo, L. L.; Pistarà, S.; Condorelli, G., Photoreduction of cerium(IV) hexachloride in acetonitrile. *J. Photochem.* **1983,** *21* (1), 45-51.
3. Yin, H.; Jin, Y.; Hertzog, J. E.; Mullane, K. C.; Carroll, P. J.; Manor, B. C.; Anna, J. M.; Schelter, E. J., The Hexachlorocerate(III) Anion: A Potent, Benchtop Stable, and Readily Available Ultraviolet A Photosensitizer for Aryl Chlorides. *J. Am. Chem. Soc.* **2016,** *138* (50), 16266-16273.
4. Vasudevan, D.; Wendt, H., Electroreduction of oxygen in aprotic media. *J. Electroanal. Chem.* **1995,** *392* (1), 69-74.
5. Wilkinson, F.; Abdel-Shafi, A. A., Mechanism of Quenching of Triplet States by Oxygen: Biphenyl Derivatives in Acetonitrile. *J. Phys. Chem. A* **1997,** *101* (30), 5509-5516.
6. Ihee, H., Visualizing Solution-Phase Reaction Dynamics with Time-Resolved X-ray Liquidography. *Acc. Chem. Res.* **2009,** *42* (2), 356-366.
7. Kim, T. K.; Lee, J. H.; Wulff, M.; Kong, Q.; Ihee, H., Spatiotemporal Kinetics in Solution Studied by Time-Resolved X-Ray Liquidography (Solution Scattering). *ChemPhysChem* **2009,** *10* (12), 1958-1980.
8. James, F.; Roos, M., Minuit - a system for function minimization and analysis of the parameter errors and correlations. *Comput. Phys. Commun.* **1975,** *10* (6), 343-367.
9. Ihee, H.; Wulff, M.; Kim, J.; Adachi, S.-i., Ultrafast X-ray scattering: structural dynamics from diatomic to protein molecules. *Int. Rev. Phys. Chem.* **2010,** *29* (3), 453-520.
10. Lee, C.; Yang, W.; Parr, R. G., Development of the Colle-Salvetti correlation-energy formula into a functional of the electron density. *Phys. Rev. B* **1988,** *37* (2), 785-789.
11. Becke, A. D., Density-functional thermochemistry. III. The role of exact exchange. *J. Chem. Phys.* **1993,** *98* (7), 5648-5652.
12. Adamo, C.; Barone, V., Toward reliable density functional methods without adjustable parameters: The PBE0 model. *J. Chem. Phys.* **1999,** *110* (13), 6158-6170.
13. Chai, J.-D.; Head-Gordon, M., Long-range corrected hybrid density functionals with damped atom–atom dispersion corrections. *Phys. Chem. Chem. Phys.* **2008,** *10* (44), 6615-6620.
14. Yanai, T.; Tew, D. P.; Handy, N. C., A new hybrid exchange–correlation functional using the Coulomb-attenuating method (CAM-B3LYP). *Chem. Phys. Lett.* **2004,** *393* (1), 51-57.
15. Weigend, F.; Ahlrichs, R., Balanced basis sets of split valence, triple zeta valence and quadruple zeta valence quality for H to Rn: Design and assessment of accuracy. *Phys. Chem. Chem. Phys.* **2005,** *7* (18), 3297-3305.
16. Barone, V.; Cossi, M., Quantum Calculation of Molecular Energies and Energy Gradients in Solution by a Conductor Solvent Model. *J. Phys. Chem. A* **1998,** *102* (11), 1995-2001.
17. Cossi, M.; Rega, N.; Scalmani, G.; Barone, V., Energies, structures, and electronic properties of molecules in solution with the C-PCM solvation model. *J. Comput. Chem.* **2003,** *24* (6), 669-681.
18. van Lenthe, E.; Baerends, E. J.; Snijders, J. G., Relativistic regular two-component Hamiltonians. *J. Chem. Phys.* **1993,** *99* (6), 4597-4610.
19. van Lenthe, E.; Baerends, E. J.; Snijders, J. G., Relativistic total energy using regular approximations. *J. Chem. Phys.* **1994,** *101* (11), 9783-9792.
20. van Wüllen, C., Molecular density functional calculations in the regular relativistic approximation: Method, application to coinage metal diatomics, hydrides, fluorides and chlorides, and comparison with first-order relativistic calculations. *J. Chem. Phys.* **1998,** *109* (2), 392-399.
21. Pantazis, D. A.; Chen, X.-Y.; Landis, C. R.; Neese, F., All-Electron Scalar Relativistic Basis Sets for Third-Row Transition Metal Atoms. *J. Chem. Theory Comput.* **2008,** *4* (6), 908-919.
22. Neese, F.; Wennmohs, F.; Hansen, A.; Becker, U., Efficient, approximate and parallel Hartree–Fock and hybrid DFT calculations. A ‘chain-of-spheres’ algorithm for the Hartree–Fock exchange. *Chem. Phys.* **2009,** *356* (1), 98-109.
23. Izsák, R.; Neese, F., An overlap fitted chain of spheres exchange method. *J. Chem. Phys.* **2011,** *135* (14), 144105.
24. Weigend, F., Accurate Coulomb-fitting basis sets for H to Rn. *Phys. Chem. Chem. Phys.* **2006,** *8* (9), 1057-1065.
25. Neese, F.; Wennmohs, F.; Becker, U.; Riplinger, C., The ORCA quantum chemistry program package. *J. Chem. Phys.* **2020,** *152* (22), 224108.
26. Neese, F., Software update: The ORCA program system—Version 5.0. *WIREs Computational Molecular Science* **2022,** *12* (5), e1606.
27. Frisch, M. J.; Trucks, G. W.; Schlegel, H. B.; Scuseria, G. E.; Robb, M. A.; Cheeseman, J. R.; Scalmani,

G.; Barone, V.; Petersson, G. A.; Nakatsuji, H.; Li, X.; Caricato, M.; Marenich, A. V.; Bloino, J.; Janesko, B. G.; Gomperts, R.; Mennucci, B.; Hratchian, H. P.; Ortiz, J. V.; Izmaylov, A. F.; Sonnenberg, J. L.; Williams; Ding, F.; Lipparini, F.; Egidi, F.; Goings, J.; Peng, B.; Petrone, A.; Henderson, T.; Ranasinghe, D.; Zakrzewski, V. G.; Gao, J.; Rega, N.; Zheng, G.; Liang, W.; Hada, M.; Ehara, M.; Toyota, K.; Fukuda, R.; Hasegawa, J.; Ishida, M.; Nakajima, T.; Honda, Y.; Kitao, O.; Nakai, H.; Vreven, T.; Throssell, K.; Montgomery Jr., J. A.; Peralta, J. E.; Ogliaro, F.; Bearpark, M. J.; Heyd, J. J.; Brothers, E. N.; Kudin, K. N.; Staroverov, V. N.; Keith, T. A.; Kobayashi, R.; Normand, J.; Raghavachari, K.; Rendell, A. P.; Burant, J. C.; Iyengar, S. S.; Tomasi, J.; Cossi, M.; Millam, J. M.; Klene, M.; Adamo, C.; Cammi, R.; Ochterski, J. W.; Martin, R. L.; Morokuma, K.; Farkas, O.; Foresman, J. B.; Fox, D. J. *Gaussian 16 Rev. C.01*, Wallingford, CT, 2016.

28. Refson, K., Moldy: a portable molecular dynamics simulation program for serial and parallel computers. *Comput. Phys. Commun.* **2000,** *126* (3), 310-329.

29. Bernardi, E.; Stassen, H., Molecular dynamics simulations of acetonitrile/dimethyl sulfoxide liquid mixtures. *J. Chem. Phys.* **2004,** *120* (10), 4860-4867.

30. Rappe, A. K.; Casewit, C. J.; Colwell, K. S.; Goddard, W. A., III; Skiff, W. M., UFF, a full periodic table force field for molecular mechanics and molecular dynamics simulations. *J. Am. Chem. Soc.* **1992,** *114* (25), 10024-10035.

31. Villa, I.; Sanchez, F.; Lopes, T.; Lopez-Cornejo, P.; Perez-Tejeda, P., Photoinduced Electron-Transfer Reactions: A Study of the Diffusion-Controlled and Activation-Diffusion-Controlled Processes. *J. Phys. Chem. A* **2010,** *114* (30), 7912-7917.

32. Rice, S. A., *Diffusion-limited reactions*. Elsevier: 1985;
